# Atlas of expression of acyl CoA binding protein/diazepam binding inhibitor (ACBP/DBI) in human and mouse

**DOI:** 10.1038/s41419-025-07447-w

**Published:** 2025-02-26

**Authors:** Sijing Li, Silvia Mingoia, Léa Montégut, Flavia Lambertucci, Hui Chen, Yanbing Dong, Fatima Domenica Elisa De Palma, Sarah Adriana Scuderi, Yan Rong, Vincent Carbonnier, Isabelle Martins, Maria Chiara Maiuri, Guido Kroemer

**Affiliations:** 1 Centre de Recherche des Cordeliers, Équipe Labellisée par la Ligue Contre le Cancer, Université Paris Cité, Sorbonne Université, Inserm U1138, Institut Universitaire de France, Paris, France; 2 Metabolomics and Cell Biology Platforms, Institut Gustave Roussy, Villejuif, France; 3 Department of Pharmacological Sciences, University of Piemonte Orientale, Novara, Italy; 4 Faculté de Médecine, Université de Paris Saclay, Kremlin Bicêtre, Paris, France; 5 Department of Molecular Medicine and Medical Biotechnologies, University of Napoli Federico II, Napoli, Italy; 6 Department of Chemical, Biological, Pharmaceutical and Environmental Sciences, University of Messina, Messina, Italy; 7 Institut du Cancer Paris CARPEM, Department of Biology, Hôpital Européen Georges Pompidou, AP-HP, Paris, France

**Keywords:** Macroautophagy, Molecular biology

## Abstract

Acyl CoA binding protein encoded by diazepam binding inhibitor (ACBP/DBI) is a tissue hormone that stimulates lipo-anabolic responses and inhibits autophagy, thus contributing to aging and age-related diseases. Protein expression profiling of ACBP/DBI was performed on mouse tissues to identify organs in which this major tissue hormone is expressed. Transcriptomic and proteomic data bases corroborated a high level of human-mouse interspecies conservation of ACBP/DBI expression in different organs. Single-cell RNA-seq data confirmed that ACBP/DBI was strongly expressed by parenchymatous cells from specific human and mouse organs (e.g., kidney, large intestine, liver, lung) as well as by myeloid or glial cells from other organs (e.g., adipose tissue, brain, eye) following a pattern that was conserved among the two species. We identified a panel of 44 mRNAs that are strongly co-expressed with ACBP/DBI mRNA in normal and malignant human and normal mouse tissues. Of note, 22 (50%) of these co-expressed mRNAs encode proteins localized at mitochondria, and mRNAs with metabolism-related functions are strongly overrepresented (66%). Systematic data mining was performed to identify transcription factors that regulate ACBP/DBI expression in human and mouse. Several transcription factors, including growth response 1 (EGR1), E2F Transcription Factor 1 (E2F1, which interacts with retinoblastoma, RB) and transformation-related protein 53 (TRP53, best known as p53), which are endowed with oncosuppressive effects, consistently repress ACBP/DBI expression as well as its co-expressed mRNAs across multiple datasets, suggesting a mechanistic basis for a coregulation network. Furthermore, we identified multiple transcription factors that transactivate ACBP/DBI gene expression together with its coregulation network. Altogether, this study indicates the existence of conserved mechanisms determining the expression of ACBP/DBI in specific cell types of the mammalian organism.

## Background

Acyl CoA binding protein encoded by the diazepam binding inhibitor gene (ACBP/DBI) is a phylogenetically conserved tissue hormone [1–4]. As its dual name indicates, ACBP/DBI is usually found as an intracellular protein in the cytoplasm where it binds to activated fatty acids and other lipids to participate in their metabolism and to stimulate fatty acid oxidation [5–8]. However, this small protein (13 kDa for the dominant isoform 1) [9] can also be found in the extracellular space including in the plasma where it acts as a ligand of a specific isoform (gamma-2) of the gamma-amino butyric acid (GABA) receptor type A (GABA_A_R) complex, abbreviated GABRG2, to modulate chloride fluxes [10, 11]. Through its effects on GABRG2, extracellular ACBP/DBI then acts on cells to limit autophagy [1, 12, 13] and also elicits organism-wide metabolic responses consisting in enhanced food intake and lipoanabolism [1, 14]. Of note, ACBP/DBI is a leaderless protein, meaning that it cannot undergo conventional protein secretion [15, 16]. Rather, it is either passively released from dying cells or actively secreted through an unconventional, autophagy-dependent pathway [14, 17].

Much of the literature on ACBP/DBI has been concentrating on its role as a precursor of neuropeptides, including octadecaneuropeptide (ODN) secreted by astrocytes in the central nervous system, where it then acts on GABRG2 as well as on a yet-to-be-characterized G protein coupled receptor expressed by hypothalamic neurons to inhibit appetite [2, 18–21]. In sharp contrast, peripherally (i.e., intravenously or intraperitoneally) administered recombinant ACBP/DBI protein, which does not cross the blood-brain barrier, exclusively stimulates appetite through an action on GABRG2 [1, 14]. ACBP/DBI concentrations increase in the plasma upon short-term fasting, as well as in obesity, both in mice and in humans [1, 22]. These observations led to the suggestion that ACBP/DBI might be both involved in the ‘hunger reflex’ (which links starvation to food-seeking behavior) [23] and contribute to the pathogenesis of obesity and its associated metabolic syndrome [24]. Indeed, inhibition of extracellular ACBP/DBI with monoclonal antibodies can reduce food intake and prevent high-fat diet-induced dyslipidemia and insulin resistance [1, 12].

Importantly, antibody-mediated neutralization of extracellular ACBP/DBI results in the inhibition of its function as an autophagy checkpoint and hence stimulates autophagy to protect the heart, liver and lung against physical or chemical damage [1, 12, 13, 25]. This effect is mediated by the capacity of autophagy to reduce unwarranted cell death and to suppress inflammation as well as fibrosis [26–30]. ACBP/DBI plasma concentrations increase with human aging [22, 31, 32], suggesting that ACBP/DBI might act as a gerogene and hence contribute to the aging process. Accordingly, knockout of the genes coding for ACBP/DBI orthologs induces autophagy and enhances lifespan in yeast and nematodes [32–35]. Moreover, neutralization of ACBP/DBI prevents cardiac aging [32] and improves cancer immunosurveillance in mice [36], suggesting that ACBP/DBI inhibition can combat age-associated diseases.

However, the exact mechanisms of these effects are not known. For example, even though ACBP/DBI neutralization protects the heart against damage by ischemia or anthracyclines [12, 32], it is not clear which cell types produce/secrete ACBP/DBI in physiological and pathological conditions and on which cell types ACBP/DBI acts. Similarly, it is not known which cells in the immune system produce ACBP/DBI to explain its immunosuppressive and pro-inflammatory effects [12, 32]. As a first step to elucidate this question, we decided to establish an atlas of ACBP/DBI expression.

## Results

### Human-mouse conservation of ACBP/DBI expression at the organ level

**Fig. 1:**
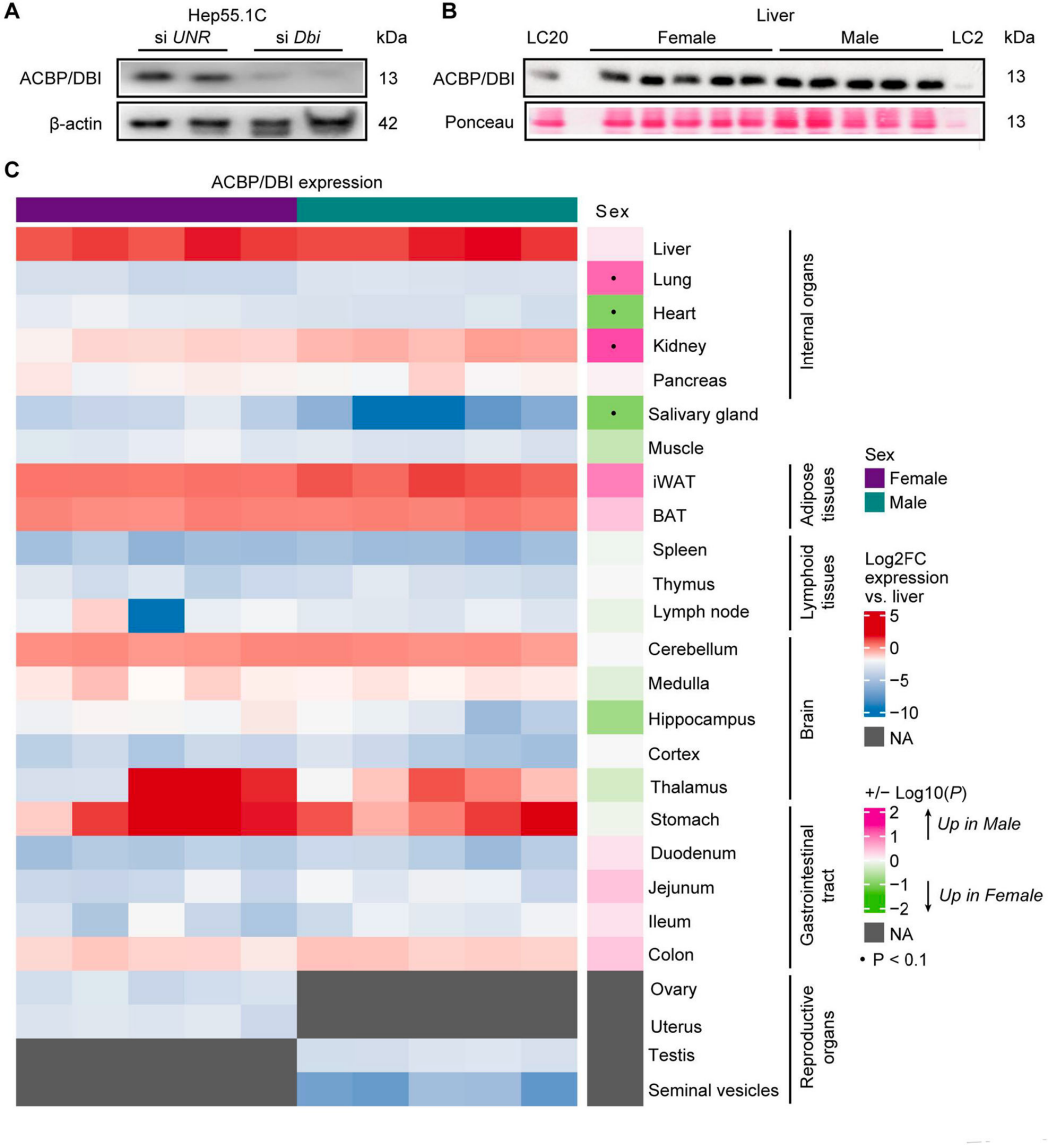
Immunoblot detection of ACBP/DBI in mouse tissues. **A** The 13 kDa band corresponding to ACBP/DBI is detected in Hep55.1C cells transiently transfected with control unrelated small interfering RNAs (siUNR) and vanished when knocking down *Dbi* expression with *Dbi*-specific small interfering RNAs (si*Dbi*). Beta-actin is showed as loading control. The images are from 1 experiment and representative of 2 experiments. **B** Immunoblots of ACBP/DBI in liver tissues from female and male mice (*N* = 5 per group). A protein loading control (LC) was loaded at 2 µg (LC2) or 20 µg (LC20) for relative normalization between membranes. **C** Heatmap of the log2-scaled normalized ACBP/DBI protein expression across female and male mouse tissues. Unpaired two-sided Student *t*-test with Benjamini–Hochberg correction for multiple comparison was used to test for significance. ^.^*P* < 0.1.

A polyclonal ACBP/DBI-specific antibody detected one major ~13 kDa band in protein extracts from mouse hepatocellular cancer Hep55.1C cells. This signal was strongly attenuated upon knockdown of the mouse *Dbi* gene using a small interfering RNA (Fig. 1A), supporting the specificity of the ACBP/DBI immunoblot detection. This antibody also recognized a ~ 13 kDa band in livers from female and male C57Bl/6 mice without any sex-specific differences (Fig. 1B). We performed immunoblots in a relative quantification on multiple distinct tissues. Bands corresponding to ACBP/DBI were detected in all tissues, though with a rather strong inter-organ variability (Supplemental Fig. S1). Expression was highest in liver, kidney, white adipose tissue (WAT), brown adipose tissue (BAT), various portions of the central nervous system (cerebellum, medulla, thalamus), and portions of the gastrointestinal tract (stomach and colon). Some sexual dimorphism may occur in the sense that ACBP/DBI expression is slightly augmented in the female heart, female salivary gland, male kidney and male lung than in organs from the opposed sex (Fig. 1C and Supplemental Fig. S1).

**Fig. 2:**
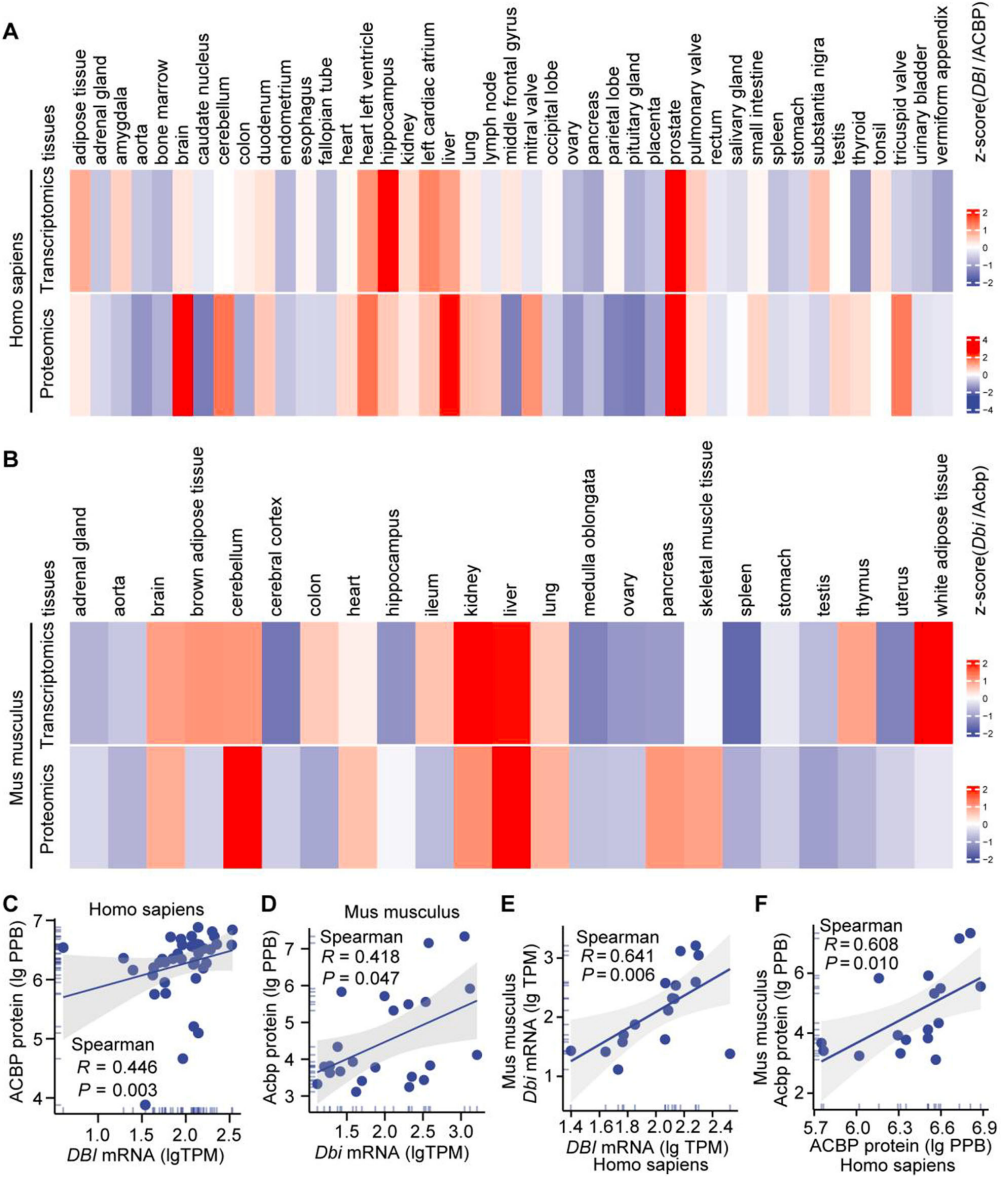
Organ-specific expression profile of ACBP/*DBI* in human and mouse. Relative mRNA and protein expression levels of ACBP/DBI across different human (**A**) and mouse tissues (**B**). Data were obtained from the Human Protein Atlas (HPA, https://www.proteinatlas.org/), Genotype-Tissue Expression (GTEx, https://www.gtexportal.org/home/), Expression Atlas (https://www.ebi.ac.uk/gxa/home), and ArrayExpress (https://www.ebi.ac.uk/biostudies/arrayexpress) datasets. **C**–**F** Correlation analysis. Correlations of ACBP/*DBI* or Acbp/*Dbi* between mRNA and protein levels in human (**C**) and mouse tissue samples (**D**). Correlations of *DBI/Dbi* or ACBP/Acbp between human and mouse tissue samples at the mRNA (**E**) and protein levels (**F**). Correlation analysis was performed using Spearman’s correlation test. TPM transcripts per kilobase million, PPB parts per billion.

We retrieved data on ACBP/DBI mRNA and protein expression from publicly available databases dealing with human (Fig. 2A) and mouse tissues (Fig. 2B). These data largely confirm our immunoblot analyses shown (Fig. 1 and Supplemental Fig. S1). Both in human and mouse, the levels of ACBP/DBI mRNA and protein correlated to some extent, with Spearman correlation coefficients >0.4 (Fig. 2C,D). Of note, the interspecies cross-correlation between human and mouse was even higher, with Spearman correlation coefficients >0.6, both for mRNA (Fig. 2E) and protein (Fig. 2F).

These findings indicate a significant level of human-mouse conservation of ACBP/DBI expression at the organ level.

### Human-mouse conservation of ACBP/DBI expression at the single-cell level

**Fig. 3:**
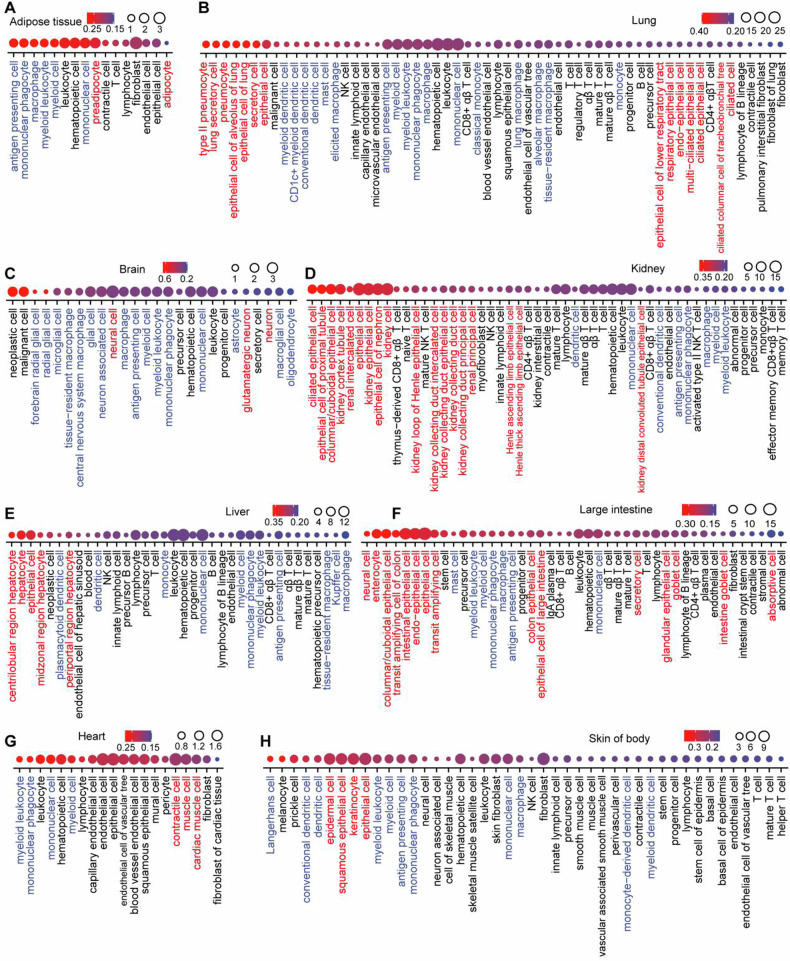
Gene expression profile of ACBP/*DBI* at the single-cell level across distinct human tissues. The single-cell expression of ACBP/DBI in human tissues, including adipose tissue (**A**), lung (**B**), brain (**C**), kidney (**D**), liver (**E**), large intestine (**F**), heart (**G**), and skin (**H**), is shown in dot plots. The dot color indicates scaled ACBP/DBI expression (ln(CPTT + 1). The dot size represents ACBP/DBI expressed in cells (%, tissue composition). The single-cell RNA data were obtained from CZ CELLxGENE database (https://cellxgene.cziscience.com/). CPTT: scaled pseudocounts. Note that parenchymatous cell types are marked in red, while myeloid and glial cell types are marked in blue.

**Fig. 4:**
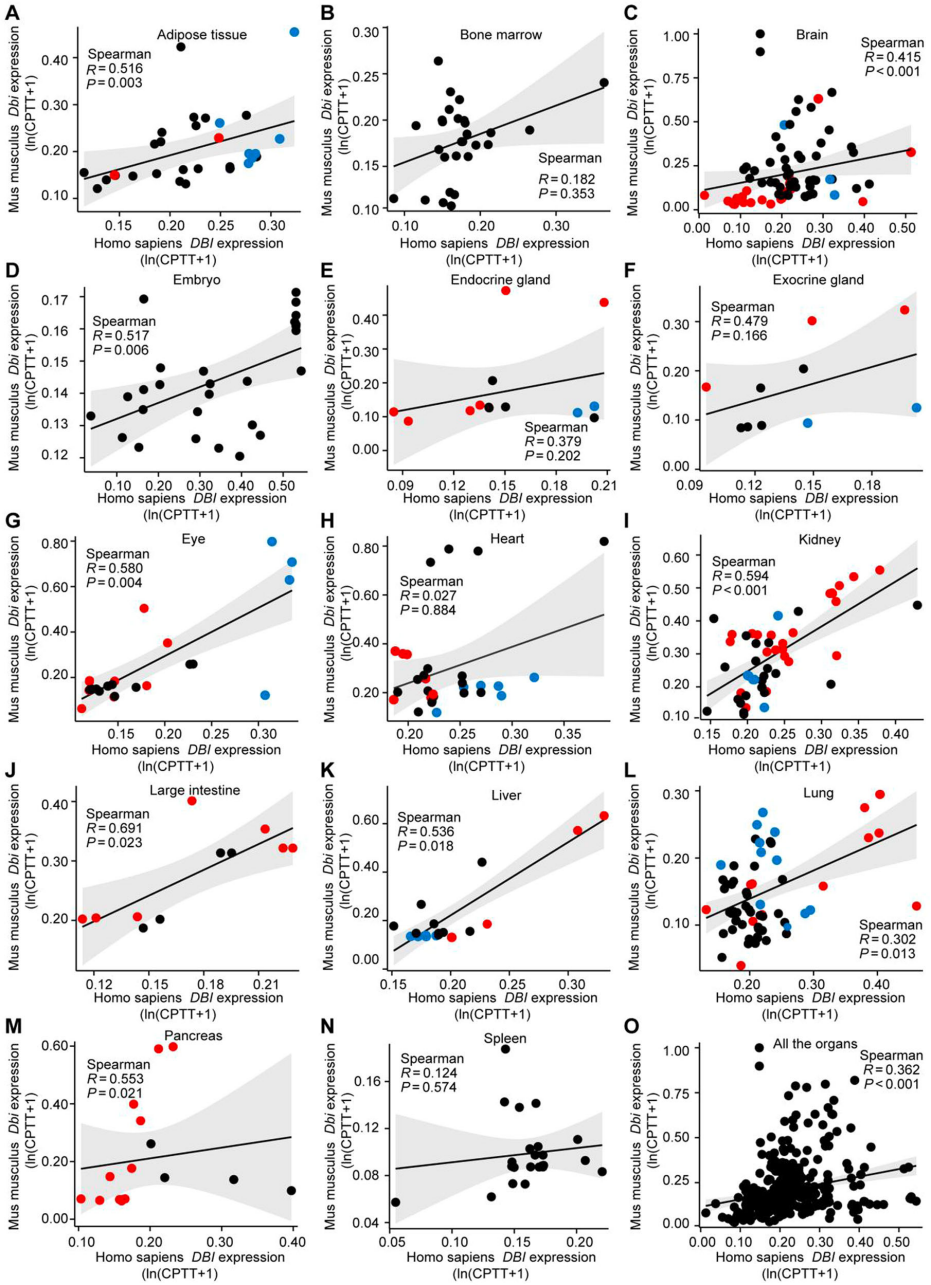
*C*orrelations between mouse *Dbi* and human *DBI* at single cell levels in different tissues. Single-cell RNA-seq data (referred to Figs. 3–5 and Figs. S2–S4) were used to assess the *DBI/Dbi* correlations between mouse and human in tissue samples, including adipose tissue (**A**), bone marrow (**B**), brain (**C**), embryo (**D**), endocrine gland (**E**), exocrine gland (**F**), eye (**G**), heart (**H**), kidney (**I**, **J**), liver (**K**), lung (**L**), pancreas (**M**), spleen (**N**), and across all the tissues (**O**). Correlation analysis was performed using Spearman’s correlation test. Note that parenchymatous cell types are marked in red, while myeloid and glial cell types are marked in blue.

In the next step, we retrieved single-cell RNA sequencing (scRNAseq) data from a large array of human and mouse organs. Where appropriate, we annotated parenchymatous cell types (e.g., adipocytes and preadipocytes in adipose tissue; cardiomyocytes in the heart; epithelial cells in the intestine, lung, kidney and skin; hepatocytes in the liver, neurons in the brain, photoreceptors in the eye etc.) in red, while myeloid and glial cell types were marked in blue (Fig. 3 and Supplemental Figs. S2–S7). Human-mouse comparisons were performed for all tissues for which data were available, revealing again evidence for interspecies conservation of ACBP/DBI mRNA expression at the single-cell level (Fig. 4). Of note, in some human tissues, parenchymatous cell types are strongly positive for ACBP/DBI expression as this is the case for kidney (Fig. 4I), large intestine (Fig. 4J), liver (Fig. 4K) and lung (Fig. 4L), in which epithelial cells express the highest level of ACBP/DBI mRNA. In contrast, in other human organs, myeloid or glial cells tend to express higher levels of ACBP/DBI mRNA than parenchymatous cell types, as this applies to adipose tissue (where only preadipocytes, but not mature adipocytes, express ACBP/DBI, Fig. 4A), as well as to brain and eye (where ACBP/DBI is highly expressed by multiple glial cell types but mostly low in neurons and photoreceptors or pigment cells, Fig. 4C and G). The aggregate of all cell types across all organs exhibits significant human-mouse conservation (Spearman correlation coefficient = 0.362, Fig. 4O), and this applies as well to specific organs including adipose tissue (Fig. 4A), brain (Fig. 4C), eye (Fig. 4G), kidney (Fig. 4I), large intestine (Fig. 4J), liver (Fig. 4K), lung (Fig. 4L) and pancreas (Fig. 4M), but not to others such as heart (Fig. 4H). Thus, in kidney, large intestine, liver and lung from mice, epithelial cells express high levels of ACBP/DBI, while in brain and eye from mice glial cells express higher levels of ACBP/DBI than parenchymatous elements such as neurons and photoreceptors, respectively.

We conclude that ACBP/DBI expression is high in parenchymatous cells of specific organs (such as kidney, large intestine, liver and lung) as well as in myeloid or glial cells from other organs (such as adipose tissue, brain and eye) and that this pattern of cell type-specific expression is conserved between human and mouse.

### Human-mouse conservation of an ACBP/DBI co-expression network

**Fig. 5:**
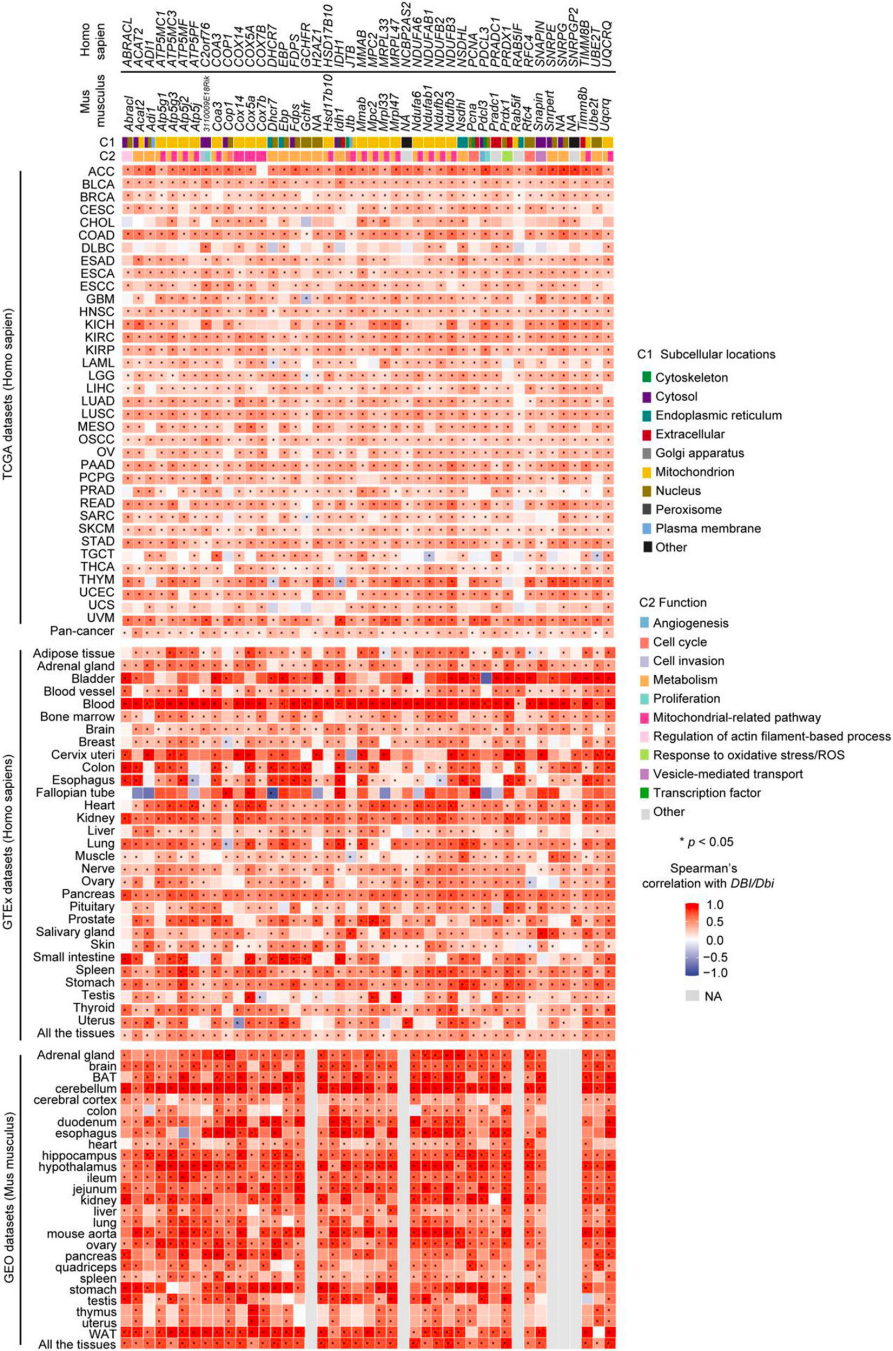
Correlations of ACBP/*DBI* and its co-expressed genes across different cancers and normal tissues. The RNA-seq expression data from the TCGA (https://www.cancer.gov/ccg/research/genome-sequencing/tcga), GTEx (https://www.gtexportal.org/home/), GEO (https://www.ncbi.nlm.nih.gov/geo/), and ArrayExpress (https://www.ebi.ac.uk/biostudies/arrayexpress) datasets were used to analyze the correlations between ACBP/DBI and the previously established list of its co-expressed genes in both human and mouse tissues. Correlation analysis was performed using Spearman’s correlation test.

The co-expressed genes of ACBP/DBI are a panel of genes that show similar expression pattern with ACBP/DBI across samples. We identified a panel of 44 transcripts that are co-expressed with ACBP/DBI through the Spearman’s correlation analysis in combination with ACBP/DBI-based differential expression analyses across 36 human cancer types listed in The Cancer Genome Atlas (TCGA). This positive correlation between ACBP/DBI and its co-expressed genes was further validated in all normal human tissues available in the GTEx database. For 38 of these genes, gene expression information was available for normal mouse tissues from the GEO dataset, and all these 38 mRNA species exhibited significant positive correlation with mouse *Dbi* as well (Fig. 5). These results plead in favor of the existence of an ACBP/DBI co-expression network that is conserved between human and mouse.

Of note, half of the 44 genes the expression of which correlates with human or mouse *DBI* code for proteins localized at mitochondria, as this applies to acetyl-CoA acetyltransferase 2 (ACAT2), several subunits of the mitochondrial ATP synthase (ATP5MC1, ATP5MC3, ATP5MF, ATP5PF), a subunit of the cytochrome c oxidase assembly factor (COA3), several subunits of cytochrome c oxidase (COX13, COX5A, COX7B), hydroxysteroid (17β) dehydrogenase 10 (HSD17B10, which is involved in the oxidation of isoleucine, branched fatty acids and neuroactive steroids), jumping translocation breakpoint (JTB), metabolism of cobalamin associated B (MMAB, which catalyzes the final step in the conversion of vitamin B12 into adenosylcobalamin, a coenzyme for methylmalonyl-CoA mutase), mitochondria pyruvate carrier 2 (MPC2), mitochondrial ribosomal proteins L33 and L47 (MRPL33 and MRLP47), several NADH dehydrogenase subunits (NDUFA6, NDUFAB1, NDUFB2, NDUFB3), RAB5 interacting factor (RAB5IF, an assembly factor for mitochondrial respiratory complexes), translocase of inner mitochondrial membrane 8 homolog B (TIMM8B, which participates to the import and insertion of some multi-pass transmembrane proteins into the mitochondrial inner membrane), and ubiquinol-cytochrome c reductase complex III subunit VII (UQCRQ) (Fig. 5). Hence, multiple genes coding for protein subunits of respiratory chain complexes I, III, IV, and V, the import or assembly of such respiratory chain complexes, as well as genes required for funneling metabolites of glucose, fatty acids, protein, and amino acids towards the tricarboxylic acid cycle are co-expressed with *DBI*. Other metabolism-relevant genes that are co-expressed with *DBI* include a heterogeneous collection of genes coding for acireductone dioxygenase 1 (ADI1), COP1 E3 ubiquitin ligase (COP1), 7-dehydrocholesterol reductase (DHCR7), EBP cholestenol delta-isomerase (EBP), farnesyl diphosphate synthase (FDPS), GTP cyclohydrolase I feedback regulator (GCHFR), H2A.Z variant histone 1 (H2AZ1), isocitrate dehydrogenase 1 (IDH1), NAD(P) dependent steroid dehydrogenase-like (NSDHL), small nuclear ribonucleoprotein polypeptides E and G (SNRPE and SNRPG), and ubiquitin conjugating enzyme E2 F (UBE2F) (Fig. 5).

In sum, it appears that mitochondrial and metabolism-relevant genes are selectively enriched within the co-expression network of *DBI*.

### Transcription factors regulating ACBP/DBI and its co-expression network

Since ACBP/DBI itself has been described as a transcription factor [37–39], we explored several datasets in which the liver transcriptome of constitutive or conditional ACBP/DBI knockout mice was compared to that of control mice or—alternatively—the inhibition of ACBP/DBI by antibodies was assessed. However, ACBP/DBI knockout or neutralization did not lead to the coherent downregulation of any of the ACBP/DBI co-expressed mRNAs (Supplemental Fig. S8), suggesting that ACBP/DBI itself was not responsible for the transactivation of the co-expressed genes.

**Fig. 6:**
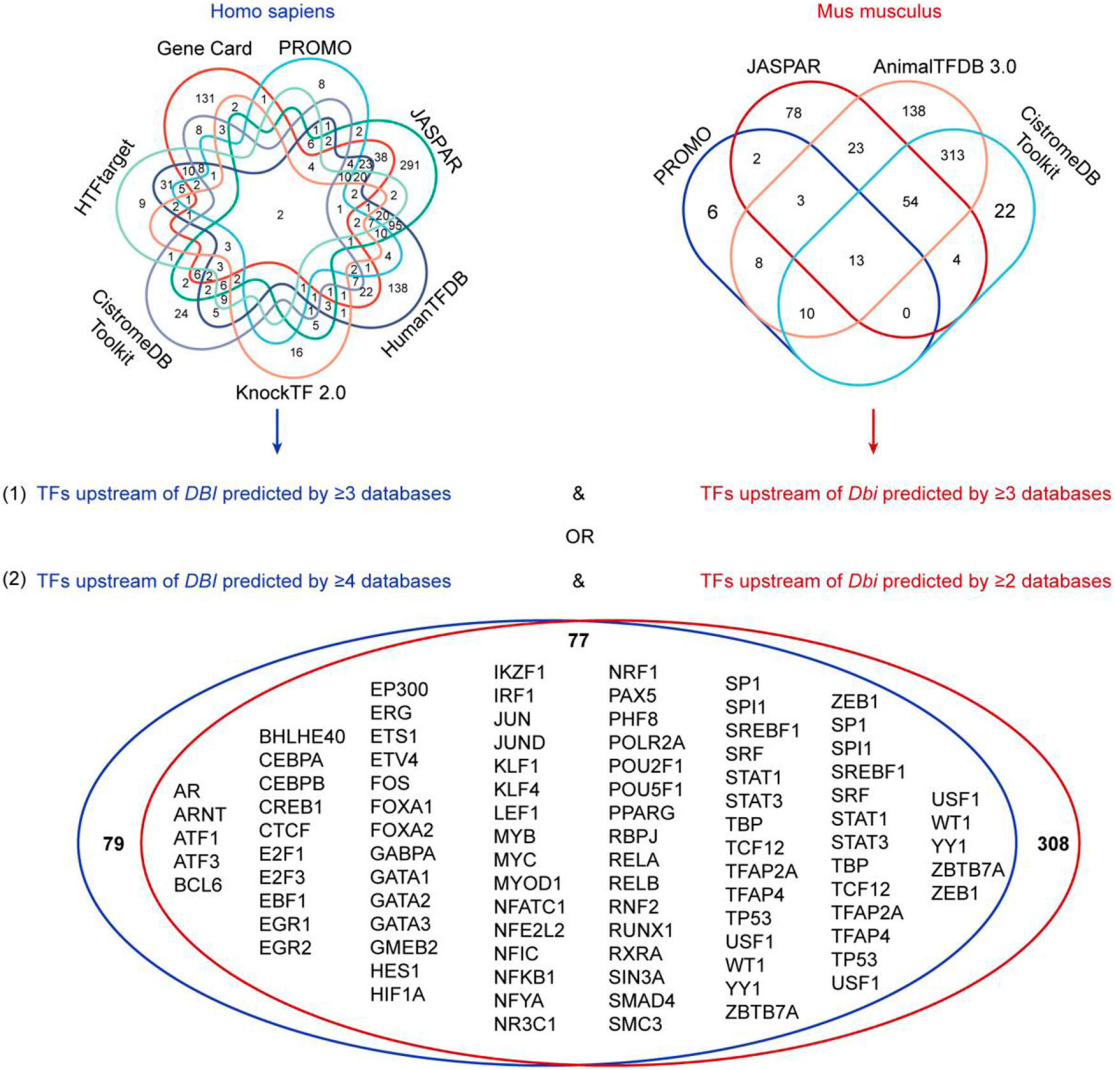
Common transcription factors upstream of ACBP/DBI in human and mouse. The potential transcription factors with binding sites at the promoter region of ACBP/DBI were predicted by multiple online tools (described in the Material & Methods section) in both human and mouse. The common transcription factors predicted by ≥ 3 datasets in both human and mouse, or by ≥ 4 datasets in human & ≥ 2 datasets in mouse were further selected and shown in the Venn diagram.

**Fig. 7:**
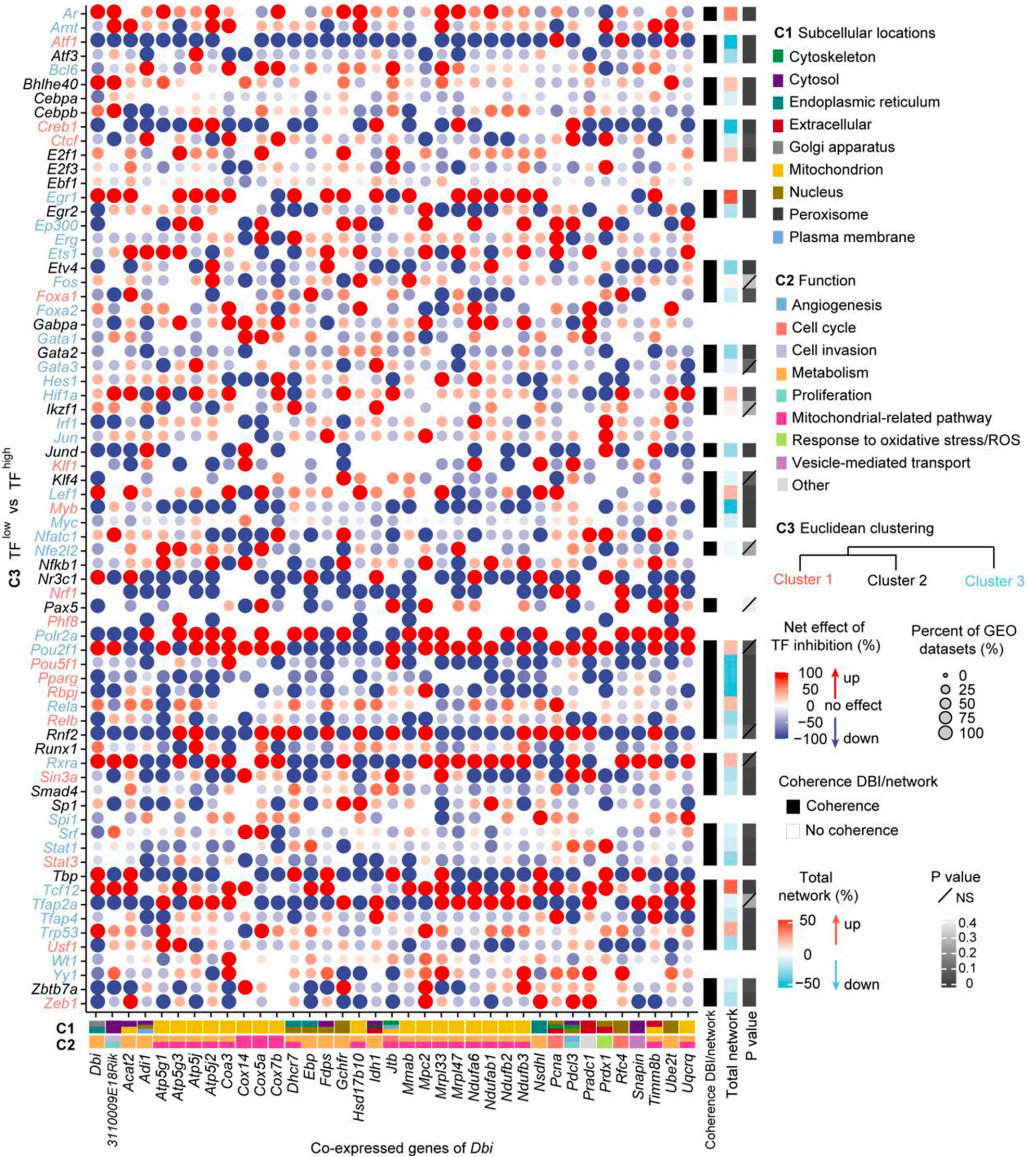
Co-regulation of ACBP/DBI and its co-expressed genes by potential transcription factors upstream of ACBP/DBI. The dot plot displays the alterations (net effect) of ACBP/DBI and its co-expressed genes upon the inhibition of each 71 transcription factors. Total network indicates the comprehensive effect on *DBI* and its co-expressed genes attributed to the inhibition of specific transcription factors. Effects of transcription factors on the total network were only calculated when there was a coherent effect on *DBI* mRNA expression and the entire network. The RNA-seq data was obtained from GEO datasets (https://www.ncbi.nlm.nih.gov/geo/). *T*-test, Welch’s *t* test, or Wilcoxon rank sum test was used to test for significance tailored to the peculiar situation of each transcription factor. NS represents non-significance.

Driven by these considerations, we explored 7 human and 4 mouse-relevant databases listing the effects of transcription factors on ACBP/DBI expression. We identified 77 high-confidence transcription factors that were annotated as (positive or negative) regulators of ACBP/DBI in ≥3 databases for both species or in ≥4 databases for human and ≥2 databases for mouse (Fig. 6). Of note, the mRNAs encoding for 71 transcription factors correlated with most of the 44 mRNAs listed in the ACBP/DBI co-expression network (Supplemental Fig. S9; for 6 transcription factors the data were not available). We then determined which among these 71 transcription factors would be able to coherently affect *DBI-*co-expressed genes in a causal fashion in the 11 aforementioned data bases annotating transcription factor effects on gene transcription. Several transcription factors stood out in this analysis revealing shared effects on *DBI* and a large portion of its co-expressed genes (Fig. 7). Thus, cyclic AMP-dependent transcription factors (ATF) ATF1 and ATF3, cyclic AMP responsive element binding protein 1 (CREB1), early growth response 2 (EGR2), ETS translocation variant 4 (ETV4), GATA binding protein 2 (GATA2), JunD (JUND), c-myc (MYC), peroxisome proliferator-activated receptor gamma (PPARG), recombination signal binding protein for immunoglobulin kappa J region (RBPJ), the NF-kB subunit RelB (RELB), POU class 5 homeobox 1 (POU5F1, best known as OCT4), paired amphipathic helix protein Sin3a (SIN3A), serum response factor (SRF), signal transducer and activator of transcription 1 (STAT1), upstream transcription factor 1 (USF1), zinc finger and BTB domain containing 7A (ZBTB7A) and zinc finger E-box binding homeobox 1 (ZEB1) apparently transactivate *DBI* and the majority among the 44 *DBI*-co-expressed genes. Conversely, androgen receptor (AR), class E basic helix-loop-helix protein 40 (BHLHE40), early growth response 1 (EGR1), E2F Transcription Factor 1 (E2F1), hypoxia inducible factor 1 subunit alpha (HIF1A), lymphoid enhancer binding factor 1 (LEF1), retinoid X receptor alpha (RXRA), transcription factor 12 (TCF12), transformation-related protein 53 (TRP53, best known as p53) consistently repress ACBP/DBI expression and the majority of the 44 co-expressed mRNAs across multiple datasets, suggesting a mechanistic basis for a coregulation network (Fig. 7).

In conclusion, it appears that *DBI*, as well as many of its co-expressed genes, are transactivated and transrepressed by specific transcription factors.

### Post-transcriptional and post-translational regulation of ACBP/DBI

We identified 10 miRNAs that may affect ACBP/DBI across two species (human and mouse). However, none of these miRNAs exhibited a coherent up- or downregulation in human cancers listed in TCGA, and all of them correlated with ACBP/DBI expression in different cancers in a heterogeneous fashion. Pan-cancer analyses suggest that 8 of the 10 candidate miRNAs negatively and significantly (*p* < 0.01) correlated with ACBP/DBI expression, suggesting that they might be involved in ACBP/DBI regulation. We also identified 9 post-transcriptional modification commonly affecting the mRNA coding for human and mouse ACBP/DBI, as well as 10 post-translational modifications affecting exactly the same amino acid residues in human and mouse ACBP/DBI protein (Supplemental Fig. S10).

This degree of phylogenetic conservation of post-transcriptional and post-translational modification strongly suggests the physiological relevance of such regulatory mechanisms.

## Discussion

In this paper, we provide an atlas of ACBP/DBI expression, as well as detailed information on its co-expression network.

We observed that specific peripheral (extra-CNS) organs are characterized by high ACBP/DBI expression at the protein level, as this was documented for adipose tissue, colon, kidney, liver and stomach from mice. Exploration of public mRNA and protein expression databanks largely confirmed these results and revealed a significant correlation of the expression levels measured in different organs from human and murine origin. Of note, these interspecies correlations (Spearman *R* values > 0.6 for both *DBI* mRNA and ACBP/DBI protein) were higher than the correlations between mRNA and protein expression within each species (Spearman *R* values < 0.5 for both human and mouse). This may indicate a significant level of organ-specific postranscriptional or posttranslational regulation of ACBP/DBI protein expression.

At the single-cell level, it turned out that, in humans, *DBI* mRNA was highly expressed in parenchymatous cells from some organs (such as subsets of epithelial cells in the colon, gall bladder, endocrine and exocrine glands, esophagus, kidney, liver, lung, kidney, nose, prostate and skin; as well as striated and smooth muscle cells in skeleton musculature and the uterus, respectively), but not in others (such as adipocytes in adipose tissue, cardiomyocytes in the heart or endothelial cells and pericytes in vasculature). In several organs, *DBI* mRNA was mostly expressed by stromal elements of the myeloid and glial type (such as adipose tissue, bladder, brain, eye, heart, pleura and vasculature). In general terms, *DBI* mRNA expression was higher in myeloid than in lymphoid immunocytes. This single-cell pattern of *DBI* mRNA expression was largely conserved between human and mouse except for the heart muscle, for which no significant correlation between the two species was observed. It remains to be determined whether this discrepancy reflects differences in data quality, for example due to postmortem recovery of tissues from humans, or methodological issues affecting cellular extraction protocols, knowing that the isolation of intact cardiomyocytes for scRNAseq is notoriously difficult [40]. This issue requires urgent clarification because inhibition of extracellular ACBP/DBI with neutralizing antibodies protects the myocardium against ischemia-induced necrosis [12, 13] as well as against anthracycline-induced senescence [25] in mice, suggesting that ACBP/DBI neutralization might constitute a promising strategy for cardioprotection in patients as well.

High abundance of ACBP/DBI correlates with the known positive effects of its antibody-mediated neutralization. Thus, ACBP/DBI inhibition has been shown to mediate cardio-, hepato- and pneumoprotective effects, correlating with the fact that heart, liver and lung expresses high levels of ACBP/DBI [12, 13, 25]. Based on the high abundance of ACBP/DBI in the kidney [41, 42], it might be expected that ACBP/DBI also affects renal function. Indeed, plasma ACBP/DBI concentrations negatively correlate with glomerular filtration rate [31, 43]. However, it has not been determined whether this reflects reduced renal elimination of ACBP/DBI or negative effects of ACBP/DBI on kidney function. Future research must clarify this issue. Similarly, it remains to be determined whether ACBP/DBI inhibition might affect the function of the gastrointestinal tract including esophagus, stomach and colon, where this protein is highly expressed [44, 45]. In this context, it will be important as well to understand which cell types express the ACBP/DBI receptor GABRG2 in these organs.

*DBI* mRNA expression correlates with that of 44 other mRNAs both in normal tissues from mouse and human, as well as in human cancers. Among these mRNAs, we observed a striking overrepresentation of genes coding for metabolically relevant proteins, in particular mitochondrion-localized proteins. Thus, the co-expression network of ACBP/DBI comprises multiple genes involved in mitochondrial biogenesis, oxidative phosphorylation and anaplerotic reactions, in accord with the major role of ACBP/DBI in intermediate metabolism. *DBI* knockout did not consistently affect the expression of these co-expressed genes, hence excluding a direct causal implication of ACBP/DBI in the regulation of gene expression.

However, we could identify several transcription factors that may explain (part of this) co-regulation network. For example, PPARG, which has been previously identified as a master transcription factor positively regulating ACBP/DBI expression in cell lines and mice [46–48] apparently also upregulates the majority (i.e., 24 out of 44) of the *DBI*-co-expressed genes. Other transcription factors that consistently upregulate *DBI* and its co-expressed mRNAs are ATF1, ATF3, CREB1, EGR2, ETV4, JUND, MYC, RBPJ, RELB, POU5F1/OCT4, SIN3A, SRF, STAT1, USF1, ZBTB7A, and ZEB1. Among these, several transcription factors stand out for their close connection to cyclic AMP signaling (ATF1, ATF3, and CREB1), although their relationship to metabolic syndrome and aging is less investigated. Other transcription factors stand out for their strong relation to inflammation, as this applies to RELB, a subunit of NF-kB that has been causally involved in metabolic syndrome and aging [49–51], MYC, which may drive inflammation in specific organ sites [52–54], as well as to STAT1, which operates in the interferon response pathway and hence contributes to aging [55, 56]. These findings suggest a causal connection between chronic inflammation and ACBP/DBI that may contribute to ‘inflammaging’ [57, 58].

Conversely, several transcription factors act as negative regulators of *DBI* mRNA expression and simultaneously inhibit most of the *DBI*-co-expressed genes, as we found for AR, BHLHE40, EGR1, E2F1, HIF1A, LEF1, RXRA, TCF12, and TRP53. Although AR was identified as a modulator of *DBI* and its co-expressed genes based on our bioinformatics analysis, we did not detect any sexual dimorphism in ACBP/DBI plasma concentrations [1, 22]. However, it is tempting to speculate that loss-of-function mutations in AR leading to complete androgen insensitivity syndrome (CAIS) cause obesity and metabolic syndrome due to the upregulation of ACBP/DBI [59, 60]. EGFR1 inhibition consistently upregulates the expression of *DBI* and 18 out of the 44 *DBI*- co-expressed genes. Knockout of EGFR1 in mice impairs liver regeneration and hepatocellular mitotic progression [61, 62] and enhances the susceptibility to acute CCl_4_ and acetaminophen‑induced hepatotoxicity [63, 64], as well as to chronic acetaminophen-induced liver fibrosis [65]. Moreover, EGFR1 is downregulated in hepatocellular carcinoma compared to normal hepatocytes, and EGFR1 plays an oncosuppressive role in multiple cancer types [66]. At this level, our data suggest that at least part of these EGFR1 effects are mediated by effects on ACBP/DBI and its co-expression network. In addition, it appears fascinating that two major transcription factors involved in oncosuppression, E2F1 (which preferentially interacts with retinoblastoma, RB) and TRP53, repress *DBI* and its co-expressed network of mRNAs, especially in view of the fact that DBI has procarcinogenic effects as demonstrated in models of glioblastoma [67], breast cancer, non-small cell lung cancer and sarcoma [67]. The hypothesis that loss-of-function mutations of RB and TRP53 favour oncogenesis through the derepression of *DBI* warrants further scrutiny. Of note, in accord with the reported capacity of TRP53 to repress ACBP/DBI mRNA expression in various databanks (GSE147709, GSE34760, GSE41601, GSE69038, GSE9144), we recently observed that patients with LiFraumeni syndrome (who bear a loss-of-function mutation of *TRP53* in their germline) have elevated plasma concentrations of ACBP/DBI [36]. In addition, we found that mice subjected to the knockout of *Trp53* exhibit elevated ACBP/DBI plasma levels [36]. These results validate this bioinformatic approach.

## Conclusions

In conclusion, the present study suggests that ACBP/DBI expression is regulated across different tissues and cell types in a phylogenetically conserved fashion, indicating that exploration of the ACBP/DBI system in mice is relevant to its pathophysiological functions in humans. Knowledge on the cell types expressing ACBP/DBI, as well as on the mechanisms explaining this expression, might yield insights into which disease(s) could be targeted by ACBP/DBI neutralization.

## Methods and materials

### Cell culture and ACBP/DBI-targeting siRNA transfection

#### Cell culture

Hep55.1C cells (Cytion, #400201) were cultured in Dulbecco’s Modified Eagle Medium (DMEM, ThermoFisher Scientific, Ref. 41966-029), supplemented with 10% Fetal Bovine Serum (FBS, Sigma-Aldrich, Ref. F7524), 10mM N-2-hydroxyethylpiperazine-N-2-ethane sulfonic acid (HEPES, ThermoFischer Scientific, Ref. 15630056) and 1 mM Sodium Pyruvate (ThermoFisher Scientific, Ref. 11360039). Cell lines were negative for mycoplasma contamination and were passaged < 10 times after the initial revival from frozen stocks.

#### Reverse siRNA transfection

Transient gene silencing was performed in 6-well cell culture plates (Costar, Ref. 3516) following the manufacturer’s protocol for Lipofectamine RNAiMAX Reagent (Invitrogen, Ref. 13778-150). Hep55.1C cells were seeded at a density of 2 × 105 cells per well in antibiotic-free medium and transfected with the siRNA complexes (30 pmol + 2.5 µL Lipofectamine RNAiMAX in 1 mL). After overnight incubation at 37 °C, fresh DMEM was added to the wells. siRNA sequences specifically targeting ACBP/DBI (Qiagen, Mm_Dbi_5 FlexiTube siRNA, NM_001037999, NM_007830), or an unrelated sequence (siUNR) control (Sigma, Ref. SIC001) were used. Cells were collected at 72 h post-transfection for analysis.

### Mouse experiment

Six- to eight-week-old C57Bl/6J mice were purchased from Envigo (France). Mice were bred and maintained according to the Federation of European Laboratory Animal Science Associations (FELASA) guidelines, as approved by the local Animal Experimental Ethics Committee (project number #25355-2020050715057113v4). The size of mice groups (*n* = 10; 5 females and 5 males) was estimated based on previous investigations [11]. Randomization was performed using the software RandoMice. Animals were allowed to acclimate for one week, provided with food ad libitum, and housed collectively in a temperature-controlled environment with 12-h light-dark cycles. Treatment-free mice were euthanized, and the following organs were collected: plasma, salivary glands, cerebellum, medulla, hippocampus, cortex, thalamus, liver, spleen, kidney, pancreas, stomach duodenum, jejunum, ileum, colon, uterus, seminal vesicles, ovaries, testicles, heart, lungs, thymus, lymph nodes, quadriceps, iWAT and BAT. Tissues were immediately snap-frozen in liquid nitrogen and stored at −80 °C. Mouse randomization and tissue harvest were performed in a blind manner.

### Protein extracts preparation

Tissues were homogenized by running 2 cycles for 20 s at 5500 rpm using a Precellys 24 tissue homogenator (Bertin Technologies, Montigny-le-Bretonneux, France) in 20 mM Tris buffer (pH 7.4) containing 150 mM NaCl, 1% Triton X-100, 10 mM EDTA and protease inhibitor (cOmplete Mini, EDTA-free, Roche, Ref. 1183617001) and phosphatase inhibitor (PhosSTOP, EASYpack, Roche, Ref. 04906837001) cocktail tablets. Protein homogenates were centrifuged at 12,000 × *g* at 4 °C for 15 min and supernatants were collected. Protein concentrations in supernatants were analyzed with a bicinchoninic acid protein kit (ThermoFisher Scientific, Ref. 23225) according to the manufacturer’s instructions.

To allow for future comparison of band intensities across organs, a protein loading control sample was generated by pooling protein lysates from all liver samples, aliquoted and stored at −20 °C. One fresh aliquot from the same stock of loading control was thawed for each run of all immunoblots presented in Figs. 1 and S1 to ensure optimal conservation.

### Immunoblot

Twenty micrograms of protein lysates were loaded and separated via SDS-PAGE for 1.5 h at a constant voltage of 120 V, within a temperature range of 18–25 °C, using MOPS SDS Running Buffer (Invitrogen, ThermoFisher Scientific, Ref. NP0001-02). Following electrophoretic separation, proteins were electrotransferred to 0.2 μm nitrocellulose membranes (Bio-Rad Laboratories, Ref. #1620112) for 1.5 h at a constant voltage of 100 V at 4 °C in Tris-Glycine (TG) buffer containing 20% ethanol. Immediately after transfer, membranes were incubated in in Ponceau S solution (Sigma-Aldrich, Ref. P7170-1L) for 5 min, rinsed, and imaged for total protein quantification. The samples were prepared in a blind manner.

Non-specific binding sites were blocked by incubating the membranes in TBST (Tris-Buffer Saline 2% + Tween 20 1%) supplemented with 5% non-fat powdered milk for 1 h at 18–25 °C, followed by three washes with TBST for 5 min each. The membranes were subsequently incubated overnight at 4 °C with mouse ACBP/DBI-specific antibody (Abcam, Ref. 231910), prepared in 3% BSA in TBST at a 1:1000 dilution or with mouse beta-actin- antibody (Abcam, Ref. 8226), prepared in 5% BSA in TBST at a 1:1000 dilution. The blots were revealed using appropriate horseradish peroxidase (HRP)-labelled secondary antibody (Southern Biotech, AL, USA) and Amersham ECL Prime substrate (Cytiva, Ref. RPN2236).

Intensities of the ACBP/DBI chemiluminescent band and of Ponceau staining were quantified on each membrane using ImageJ version 2.14.0 (National Institutes of Health, Bethesda, MD, USA). The normalization was made against Ponceau intensity (total protein) for each lane, then the loading control 2 μg (LC2) was used to obtain “relative ACBP/DBI protein expression” among the different tissues collected. Differences between male and female mice were assessed by two-sided, unpaired, Student’s *t*-test, and the resulting p values were corrected for multiple comparisons. Heatmap representation of the log2-normalized fold change of DBI normalized protein expression was plotted with the *ComplexHeatmap* package (version 2023.12.0 + 369) [68].

### ACBP/DBI expression atlas in human and mouse tissues

The human *DBI* RNA expression profile was generated from RNA consensus tissue gene data obtained from the Human Protein Atlas (HPA, https://www.proteinatlas.org/) [69] datasets. Based on the description of the HPA datasets, the RNA consensus tissue gene data is calculated as the maximum nTPM (normalized Transcripts Per Kilobase Million) value for each gene in the two data sources of the HPA and Genotype-Tissue Expression (GTEx, https://www.gtexportal.org/home/). The data source of mouse Acbp/Dbi gene expression profile is Expression Atlas (https://www.ebi.ac.uk/gxa/home). The protein tissue atlas of human and mouse ACBP/DBI (Acbp/Dbi) is generated with protein expression data derived from datasets including Expression Atlas (https://www.ebi.ac.uk/gxa/home) and ArrayExpress (https://www.ebi.ac.uk/biostudies/arrayexpress). All datasets analyzed here with accession numbers are included in Additional file 1.

### ACBP/DBI expression profile at the single-cell level

The single-cell RNA data was obtained from CZ CELLxGENE [70] database (https://cellxgene.cziscience.com/). ACBP/DBI expression across cell types in human and mouse tissues is displayed as dot plots, which visualize values in two dimensions including color and size. The color of the dot represents average ACBP/DBI gene expression. Its size indicates the percentage of cells within each tissue type that expresses ACBP/DBI gene. Correlations between human ACBP/DBI and mouse Acbp/Dbi are calculated using Spearman’s correlation test. Dot plots and correlation scatter plots were generated using the ggplot2 [3.3.6] package in the R (4.2.1) software.

### Co-expressed genes of ACBP/DBI

ACBP/DBI-based differential gene expression (DGE) was performed by using DESeq2 [version 1.26.0] package in R (4.2.1). In brief, the expression of 56493 genes in tumor samples stratified by ACBP/DBI expression (High: 50–100%, Low: 0–50%) was determined by using the RNA-seq data from TCGA datasets for 36 cancer types (https://www.cancer.gov/ccg/research/genome-sequencing/tcga). The results are shown as Log2 Fold Change (Log2 FC), ACBP/DBI: High vs Low. Next, the Spearman’s correlation coefficient (r) between ACBP/DBI (Log2 (TMP + 1)) and all the 56493 genes (Log2 (TMP + 1)) was assessed by using “stats” package in *R* (4.2.1) for 36 cancer types. Co-expressed genes of ACBP/DBI in each cancer type are identified by two conditions: (i) genes upregulated in ACBP/DBI^high^ *vs* ACBP/DBI^low^ tumor groups (log2(FC) > 0 and *P* < 0.05); (ii) genes positively associated with ACBP/DBI expression (*r* > 0.3 and *P* < 0.05). The common co-expressed genes of ACBP/DBI in ≥ 20 cancer types were identified by VennDiagram and used for further analyses. The correlations between human ACBP/DBI and its aforementioned co-expressed genes are evaluated with RNA-seq data of human normal tissues obtained from GTEx datasets (GTEx, https://www.gtexportal.org/home/). The correlations between mouse Acbp/Dbi and its co-expressed genes are determined by analyzing RNA-seq data of mouse normal tissues derived from GEO datasets (https://www.ncbi.nlm.nih.gov/geo/), as listed in additional file 1, and ArrayExpress (https://www.ebi.ac.uk/biostudies/arrayexpress). The pairwise Spearman correlation between ACBP/DBI and its co-expressed genes is visualized in correlation heatmaps. *R* (version 4.2.1) and *R* packages (GEOquery [2.64.2], limma [3.52.2], and ggplot2 [3.3.6]), are used for statistical analysis and visualization.

### ACBP/DBI and its co-expressed genes

To explore the potential effect of ACBP/DBI on its co-expressed genes, the corresponding GEO datasets of ACBP/DBI inhibition were extracted from the GEO database (https://www.ncbi.nlm.nih.gov/geo/). The differential expression of ACBP/DBI and its co-expressed genes was then analyzed and visualized using DESeq2 [version 1.26.0] and “asggplot2[3.3.6]” packages in *R* (4.2.1).

### Prediction of upstream transcription factors of ACBP/DBI

To identify the potential transcription factors (TFs) upstream of ACBP/DBI, the promoter sequence of ACBP/DBI was determined by e!Ensembl genome browser (http://www.ensembl.org/index.html). The TFs upstream of human ACBP/DBI and mouse Acbp/Dbi are then predicted by several datasets, including the HumanTFDB3.0 (http://bioinfo.life.hust.edu.cn/HumanTFDB#!/), JASPAR (https://jaspar.genereg.net/), hTFtarget (http://bioinfo.life.hust.edu.cn/hTFtarget#!/), CistromeDB Toolkit (http://dbtoolkit.cistrome.org/), PROMO (https://alggen.lsi.upc.es/recerca/frame-recerca.html), Genecard (https://www.genecards.org/), and KnockTF2.0 (http://www.licpathway.net/KnockTF/index.php). A panel of conserved TFs upstream of human and mouse ACBP/DBI with high confidence (predicted by ≥ 3 datasets in both human and mouse or by ≥ 4 datasets in human & ≥ 2 datasets in mouse) were then constructed with VennDiagram.

### Effects of transcription factors on the *DBI* co-expression network

To investigate the effect of the predicted transcription factors on *DBI* co-expression network as well as the aforementioned transcription factors, RNA-seq data associated with knockdown/knockout of these transcription factors were extracted from GEO database (referred to the additional file 2: GEO accession numbers) and analyzed with DESeq2 [version 1.26.0] package in *R* (4.2.1). Dot plots were used to display the net effect on the DBI co-expression network and these transcription factors caused by the inhibition of the transcription factors. Heatmaps present the total network and the *P* value, respectively (referred to Figs.7 and S9). *R* packages (limma [3.52.2], ggplot2 [3.3.6] and ComplexHeatmap[2.13.1]), were used for statistical analysis and visualization.

### Prediction of microRNAs targeting ACBP/DBI

The potential microRNAs (miRNAs) targeting human ACBP/DBI and mouse Acbp/Dbi were predicted by several databases, including DIANA-MicroT-CDS (https://dianalab.e-ce.uth.gr/html/dianauniverse/index.php?r=microT_CDS), TarBase v7.0 (http://diana.imis.athena-innovation.gr/DianaTools/index.php?r=tarbase/index), miRDB (https://mirdb.org/), miRWalk (http://mirwalk.umm.uni-heidelberg.de/), TargetScan v8.0 (https://www.targetscan.org/vert_80/), Starbase v2.0 (https://rnasysu.com/encori/), PITA (https://tools4mirs.org/software/target_prediction/pita/), miRmap (https://mirmap.ezlab.org/), miRanda (https://tools4mirs.org/software/target_prediction/miranda/) and TarBase v.9.0 (https://dianalab.e-ce.uth.gr/tarbasev9). The potential conserved miRNAs targeting ACBP/DBI both in human and mouse were identified by a Venn diagram. To perform the differential expression of miRNAs between tumors and non-tumor tissues, the miRNA-seq data (when available) were downloaded from TCGA (https://portal.gdc.cancer.gov) database and normalised in reads per million mapped reads (RPM) format. The associations between ACBP/DBI and miRNAs were assessed by Spearman’s correlation Rho values. The results are displayed in heatmaps generated with ggplot2[3.4.4] in *R* (4.2.1) software.

## Supplementary information

Supplemental figure and figure legends

Additional file 1

Additional file 2

Original Data

## Data Availability

The datasets used in the current study for in silico analyses were published previously. Bulk-RNAseq data associated with this study are available in the GEO repository with the accession number shown in additional file 1 and additional file 2. All other datasets and/or code generated and/or analyzed during the current study are available from the corresponding author on reasonable request.
