# Supplementary material for: Atlas of expression of acyl CoA binding protein/diazepam binding inhibitor (ACBP/DBI) in human and mouse
Source: Cell Death Dis. 2025 Feb 26;16(1):134. doi: 10.1038/s41419-025-07447-w (PMC11865319; doi:10.1038/s41419-025-07447-w)
Supplement: Supplementary file 1 — Supplemental figure and figure legends [file 41419_2025_7447_MOESM1_ESM.pdf]

Atlas of expression of acyl CoA binding protein/diazepam binding inhibitor (ACBP/DBI) in human and mouse

Sijing Li et al.

Legends to Supplemental Figures

Fig. S1. Immunoblotting of ACBP/*DBI* in mouse tissues. The differential expression of ACBP/*DBI* was assessed by immunoblotting analysis of tissue samples from female and male mice (Quantification of these results referred to Fig. 2). LC samples represent loading controls (LC2: 2µg; LC20: 20µg). n=5 per condition.

Fig. S2. Gene expression profile of ACBP/*DBI* at the single-cell level across distinct human tissues. Dot plots illustrating the single-cell expression of ACBP/DBI in human tissues, including adrenal gland (A), axilla (B), bladder organ (C), gallbladder (D), blood (E), bone marrow (F), breast (G), embryo (H), endocrine gland (I), esophagogastric junction (J), eye (K), esophagus (L), exocrine gland (M), and Fallopian tube (N). The dot color indicates scaled ACBP/DBI expression (ln(CPTT+1). The dot size represents ACBP/DBI expressed in cells (%, tissue Composition). The single-cell RNA data were obtained from CZ CELLxGENE database (https://cellxgene.cziscience.com/).

Fig. S3. Gene expression profile of ACBP/*DBI* at the single-cell level across distinct human tissues. Dot plots showing the single-cell expression of ACBP/*DBI* in human tissues, including intestine (A), lamina propria (B), omentum (C), lymph node (D), mucosa (E), musculature (F), nose (G), ovary (H), pancreas (I), placenta (J), pleura (K), pleural fluid (L), and prostate gland (M). The dot color indicates scaled ACBP/DBI expression (ln(CPTT+1). The dot size represents ACBP/DBI expressed in cells (%, tissue Composition). The single-cell RNA data were obtained from CZ CELLxGENE database (https://cellxgene.cziscience.com/). Note that parenchymatous cell types are marked in red, while myeloid and glial cell types are marked in blue.

Fig. S4. Gene expression profile of ACBP/*DBI* at the single-cell level across distinct human tissues. Dot plots showing the single-cell expression of ACBP/*DBI* in human tissues, including scalp (A), saliva (B), stomach (C), spleen (D), spinal cord (E), small intestine (F), testis (G), tongue (H), uterus (I), vasculature (J), and yolk sac (K). The dot color indicates scaled ACBP/DBI expression (ln(CPTT+1). The dot size represents ACBP/DBI expressed in cells (%, tissue Composition). The single-cell RNA data were

obtained from CZ CELLxGENE database (https://cellxgene.cziscience.com/). Note that parenchymatous cell types are marked in red, while myeloid and glial cell types are marked in blue.

Fig. S5. Gene expression profile of Acbp/*Dbi* at the single-cell level across distinct mouse tissues. Dot plots show the single-cell expression of ACBP/DBI in mouse tissues, including adipose tissue (A), bone marrow (B), brain (C), embryo (D), endocrine gland (E), exocrine gland (F), and eye (G). The dot color indicates scaled ACBP/DBI expression (ln(CPTT+1). The dot size represents ACBP/DBI expressed in cells (%, tissue Composition). The single-cell RNA data was obtained from CZ CELLxGENE database (https://cellxgene.cziscience.com/). Note that parenchymatous cell types are marked in red, while myeloid and glial cell types are marked in blue.

Fig. S6. Gene expression profile of Acbp/*Dbi* at the single-cell level across distinct mouse tissues. Dot plots display the single-cell expression of Acbp/Dbi in heart (A), large intestine (B), kidney (C), liver (D), musculature (E), ovary (F), and lung (G). The dot color indicates scaled ACBP/DBI expression (ln(CPTT+1). The dot size represents ACBP/DBI expressed in cells (%, tissue Composition). The single-cell RNA data were obtained from CZ CELLxGENE database (https://cellxgene.cziscience.com/). Note that parenchymatous cell types are marked in red, while myeloid and glial cell types are marked in blue.

Fig. S7. Gene expression profile of Acbp/*Dbi* at the single-cell level across distinct mouse tissues. Dot plots display the single-cell expression of Acbp/Dbi in pancreas (A), prostate gland (B), skin of body (C), spleen (D), testis (E), tongue (F), urethra (G), vasculature (H), and urinary bladder (I). The dot color indicates scaled ACBP/DBI expression (ln(CPTT+1). The dot size represents ACBP/DBI expressed in cells (%, tissue Composition). The single-cell RNA data were obtained from CZ CELLxGENE database (https://cellxgene.cziscience.com/). Note that parenchymatous cell types are marked in red, while myeloid and glial cell types are marked in blue.

Fig. S8. Co-regulation of Acbp/*Dbi* and its co-expressed genes by Acbp/*Dbi* inhibition. The heatmap displays the alterations of ACBP/DBI and its co-expressed genes upon the inhibition of ACBP/DBI (ACBP/DBI knockout, anti-ACBP, and KLH-ACBP) in mouse liver tissues. The RNA-seq data were obtained from GEO datasets (https://www.ncbi.nlm.nih.gov/geo/). *P* values were calculated using R package "DEseq2".

Fig. S9. Correlations of the transcription factors upstream of ACBP/*DBI* and ACBP/DBI co-expression network. The heatmap displays the correlations between the predicted 71 high-coincidence ACBP/DBI-regulating transcription factors and ACBP/DBI co-expression network (ACBP/DBI as well as its co-expressed genes) in all the mouse tissues (referred to Fig.5). Correlation analyses were performed using Spearman's correlation test.

Fig. S10. Post-transcriptional and post-translational factors affecting ACBP/DBI. (A) The predicted microRNAs targeting ACBP/DBI by more than 2 datasets conserved in human and mouse. (B) Differential expression of ACBP/DBI-targeting microRNAs between tumors (T) and non-tumor (NT) tissues when available in TCGA datasets. (C) Spearman's correlation Rho values between ACBP/DBI and microRNAs targeting ACBP/DBI across cancers in TCGA. (D) Potential chemical modifications affecting ACBP/DBI mRNA conserved in human and mouse. The information was extracted from RM2Target database (http://rm2target.canceromics.org/#/home). (E) Post-translational modifications (PTM) of human and mouse ACBP/DBI protein. The sequence alignment view of ACBP/DBI indicates similarities and differences for PTMs (acetylation, phosphorylation, succinylation or ubiquitylation) in human and mouse. The PTM sites of ACBP/DBI residues are highlighted in red lower-case letters. This information was retrieved from the PhosphoSitePlus®v6.7.5 database (https://www.phosphosite.org/homeAction).

Supplemental figures

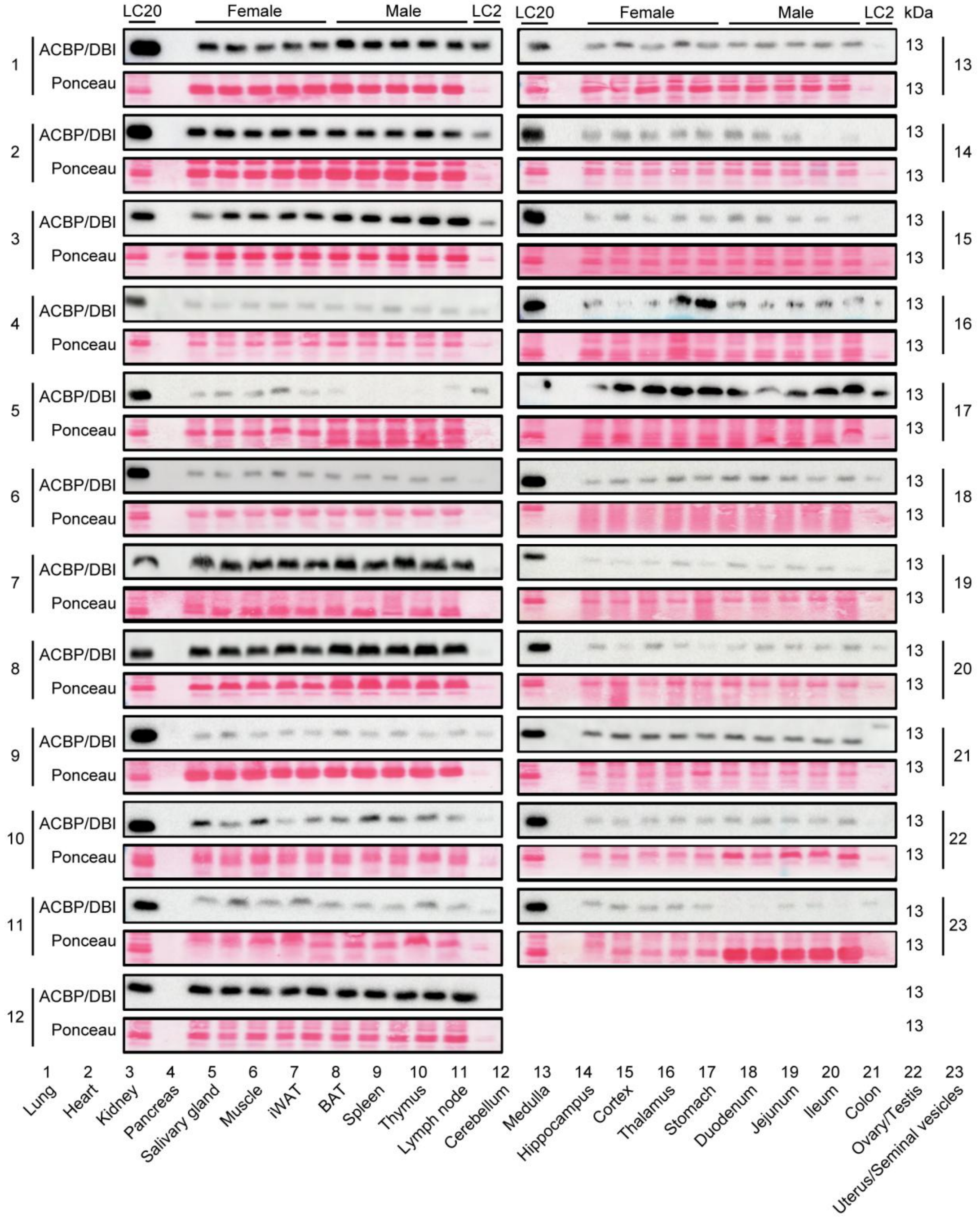

Fig. S1

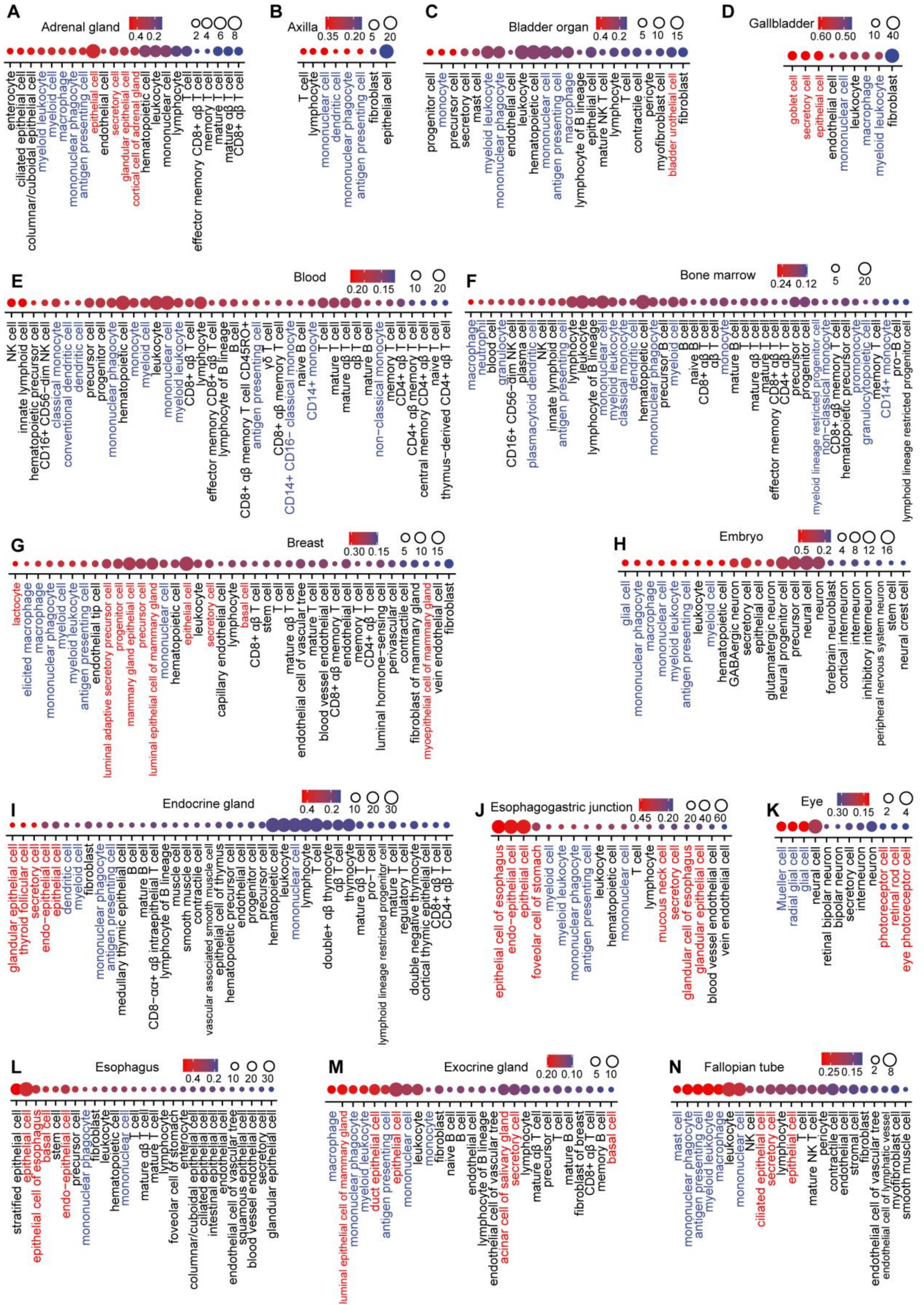
A
Adrenal gland
0.4 0.2
2 4 6 8
enterocyte
ciliated epithelial cell
columnar/cuboidal epithelial cell
myeloid leukocyte
myeloid cell
macrophage
mononuclear phagocyte
antigen presenting cell
epithelial cell
endothelial cell
secretory cell
glandular epithelial cell
cortical cell of adrenal gland
hematopoietic cell
leukocyte
mononuclear cell
lymphocyte
T cell
effector memory CD8+ αβ T cell
memory T cell
mature T cell
mature αβ T cell
CD8+ αβ T cell
B
Axilla
0.35 0.20
5 20
T cell
lymphocyte
mononuclear cell
dendritic cell
mononuclear phagocyte
antigen presenting cell
fibroblast
epithelial cell
C
Bladder organ
0.4 0.2
5 10 15
progenitor cell
monocyte
precursor cell
secretory cell
mast cell
myeloid leukocyte
mononuclear phagocyte
endothelial cell
leukocyte
hematopoietic cell
NK cell
mononuclear cell
antigen presenting cell
macrophage
lymphocyte of B lineage
epithelial cell
mature NK T cell
lymphocyte
T cell
contractile cell
pericyte
myofibroblast cell
bladder urothelial cell
fibroblast
D
Gallbladder
0.60 0.50
10 40
goblet cell
secretory cell
epithelial cell
endothelial cell
mononuclear cell
leukocyte
macrophage
myeloid leukocyte
fibroblast
E
Blood
0.20 0.15
10 20
NK cell
innate lymphoid cell
hematopoietic precursor cell
CD16+ CD56−dim NK cell
classical monocyte
conventional dendritic cell
dendritic cell
precursor cell
progenitor cell
mononuclear phagocyte
hematopoietic cell
monocyte
myeloid cell
leukocyte
mononuclear cell
myeloid leukocyte
CD8+ αβ T cell
lymphocyte
effector memory CD8+ αβ T cell
lymphocyte of B lineage
B cell
CD8+ αβ memory T cell CD45RO+
antigen presenting cell
γδ T cell
CD8+ αβ memory T cell
CD14+ CD16− classical monocyte
naive B cell
CD14+ monocyte
T cell
mature T cell
mature αβ T cell
αβ T cell
mature B cell
non-classical monocyte
memory T cell
CD4+ αβ T cell
CD4+ αβ memory T cell
central memory CD4+ αβ T cell
naive T cell
thymus-derived CD4+ αβ T cell
F
Bone marrow
0.24 0.12
5 20
macrophage
neutrophil
blood cell
granulocyte
CD16+ CD56−dim NK cell
plasma cell
plasmacytoid dendritic cell
NK cell
innate lymphoid cell
antigen presenting cell
leukocyte
lymphocyte
lymphocyte of B lineage
mononuclear cell
myeloid leukocyte
classical monocyte
dendritic cell
hematopoietic cell
mononuclear phagocyte
precursor B cell
myeloid cell
B cell
naive B cell
CD8+ αβ T cell
αβ T cell
monocyte
mature B cell
T cell
mature αβ T cell
mature T cell
effector memory CD8+ αβ T cell
CD4+ αβ T cell
precursor cell
progenitor cell
myeloid lineage restricted progenitor cell
non-classical monocyte
CD8+ αβ memory T cell
hematopoietic precursor cell
promonocyte
granulocytopoietic cell
CD14+ monocyte
memory T cell
pro-B cell
lymphoid lineage restricted progenitor cell
G
Breast
0.30 0.15
5 10 15
lactocyte
elicited macrophage
macrophage
mononuclear phagocyte
myeloid cell
myeloid leukocyte
antigen presenting cell
endothelial tip cell
luminal adaptive secretory precursor cell
progenitor cell
mammary gland epithelial cell
precursor cell
luminal epithelial cell of mammary gland
mononuclear cell
hematopoietic cell
epithelial cell
leukocyte
secretory cell
capillary endothelial cell
lymphocyte
basal cell
CD8+ αβ T cell
stem cell
T cell
mature αβ T cell
endothelial cell of vascular tree
mature T cell
blood vessel endothelial cell
CD8+ αβ memory T cell
endothelial cell
memory T cell
CD4+ αβ T cell
luminal hormone-sensing cell
perivascular cell
contractile cell
fibroblast of mammary gland
myoepithelial cell of mammary gland
vein endothelial cell
fibroblast
H
Embryo
0.5 0.2
4 8 12 16
glial cell
mononuclear phagocyte
macrophage
mononuclear cell
myeloid leukocyte
antigen presenting cell
leukocyte
myeloid cell
hematopoietic cell
GABAergic neuron
secretory cell
epithelial cell
glutamatergic neuron
neural progenitor cell
precursor cell
neural cell
neuron
forebrain neuroblast
cortical interneuron
interneuron
inhibitory interneuron
peripheral nervous system neuron
stem cell
neural crest cell
I
Endocrine gland
0.4 0.2
10 20 30
glandular epithelial cell
thyroid follicular cell
secretory cell
endo-epithelial cell
epithelial cell
dendritic cell
myeloid cell
fibroblast
mononuclear phagocyte
antigen presenting cell
medullary thymic epithelial cell
B cell
mature B cell
CD8-αα+ αβ intraepithelial T cell
lymphocyte of B lineage
muscle cell
smooth muscle cell
contractile cell
vascular associated smooth muscle cell
epithelial cell of thymus
hematopoietic precursor cell
endothelial cell
progenitor cell
precursor cell
hematopoietic cell
leukocyte
mononuclear cell
lymphocyte
T cell
double+ αβ thymocyte
αβ T cell
thymocyte
mature αβ T cell
pro-T cell
lymphoid lineage restricted progenitor cell
mature T cell
regulatory T cell
double negative thymocyte
cortical thymic epithelial cell
CD8+ αβ T cell
CD4+ αβ T cell
J
Esophagogastric junction
0.45 0.20
20 40 60
epithelial cell of esophagus
endo-epithelial cell
epithelial cell
foveolar cell of stomach
myeloid cell
myeloid leukocyte
mononuclear phagocyte
antigen presenting cell
leukocyte
hematopoietic cell
mononuclear cell
T cell
lymphocyte
mucous neck cell
secretory cell
glandular cell of esophagus
glandular epithelial cell
blood vessel endothelial cell
vein endothelial cell
K
Eye
0.30 0.15
2 4
Mueller cell
radial glial cell
glial cell
neural cell
retinal bipolar neuron
bipolar neuron
secretory cell
interneuron
neuron
photoreceptor cell
retinal rod cell
eye photoreceptor cell
L
Esophagus
0.4 0.2
10 20 30
stratified epithelial cell
epithelial cell
epithelial cell of esophagus
basal cell
stem cell
endo-epithelial cell
myeloid cell
precursor cell
mononuclear phagocyte
fibroblast
leukocyte
hematopoietic cell
mononuclear cell
T cell
mature αβ T cell
mature T cell
lymphocyte
foveolar cell of stomach
enterocyte
columnar/cuboidal epithelial cell
ciliated epithelial cell
intestinal epithelial cell
endothelial cell
endothelial cell of vascular tree
squamous epithelial cell
blood vessel endothelial cell
secretory cell
glandular epithelial cell
M
Exocrine gland
0.20 0.10
5 10
macrophage
luminal epithelial cell of mammary gland
mononuclear phagocyte
myeloid leukocyte
duct epithelial cell
antigen presenting cell
epithelial cell
mononuclear cell
leukocyte
monocyte
fibroblast
naive B cell
B cell
endothelial cell
lymphocyte of B lineage
endothelial cell of vascular tree
acinar cell of salivary gland
secretory cell
lymphocyte
mature αβ T cell
precursor cell
T cell
mature B cell
fibroblast of breast
CD8+ αβ T cell
memory B cell
basal cell
N
Fallopian tube
0.25 0.15
2 8
mast cell
mononuclear phagocyte
antigen presenting cell
myeloid leukocyte
macrophage
leukocyte
mononuclear cell
NK cell
ciliated epithelial cell
secretory cell
lymphocyte
epithelial cell
mature NK T cell
T cell
pericyte
contractile cell
endothelial cell
stromal cell
fibroblast
endothelial cell of vascular tree
endothelial cell of lymphatic vessel
myofibroblast cell
smooth muscle cell

Fig. S2

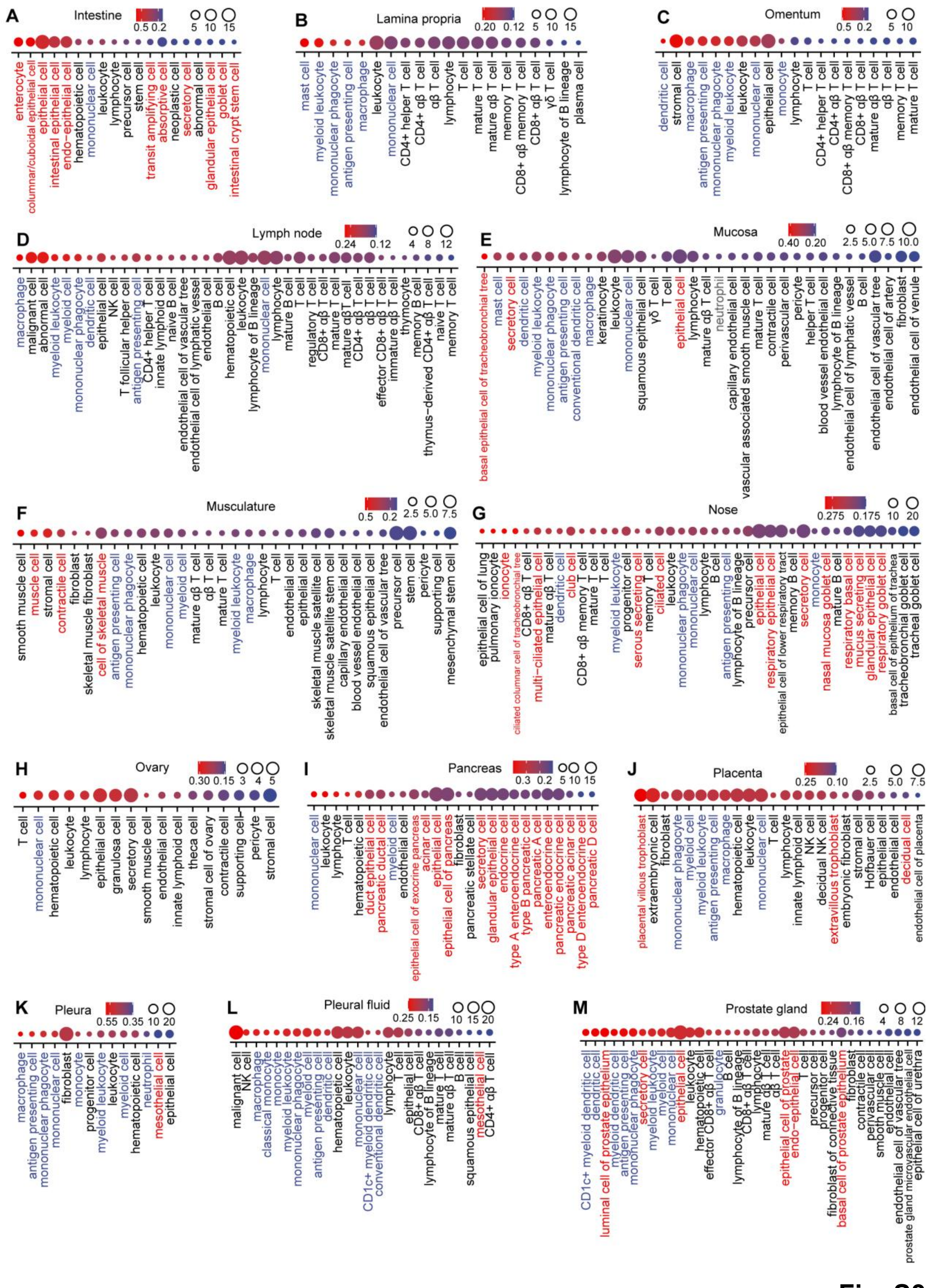

Fig. S3

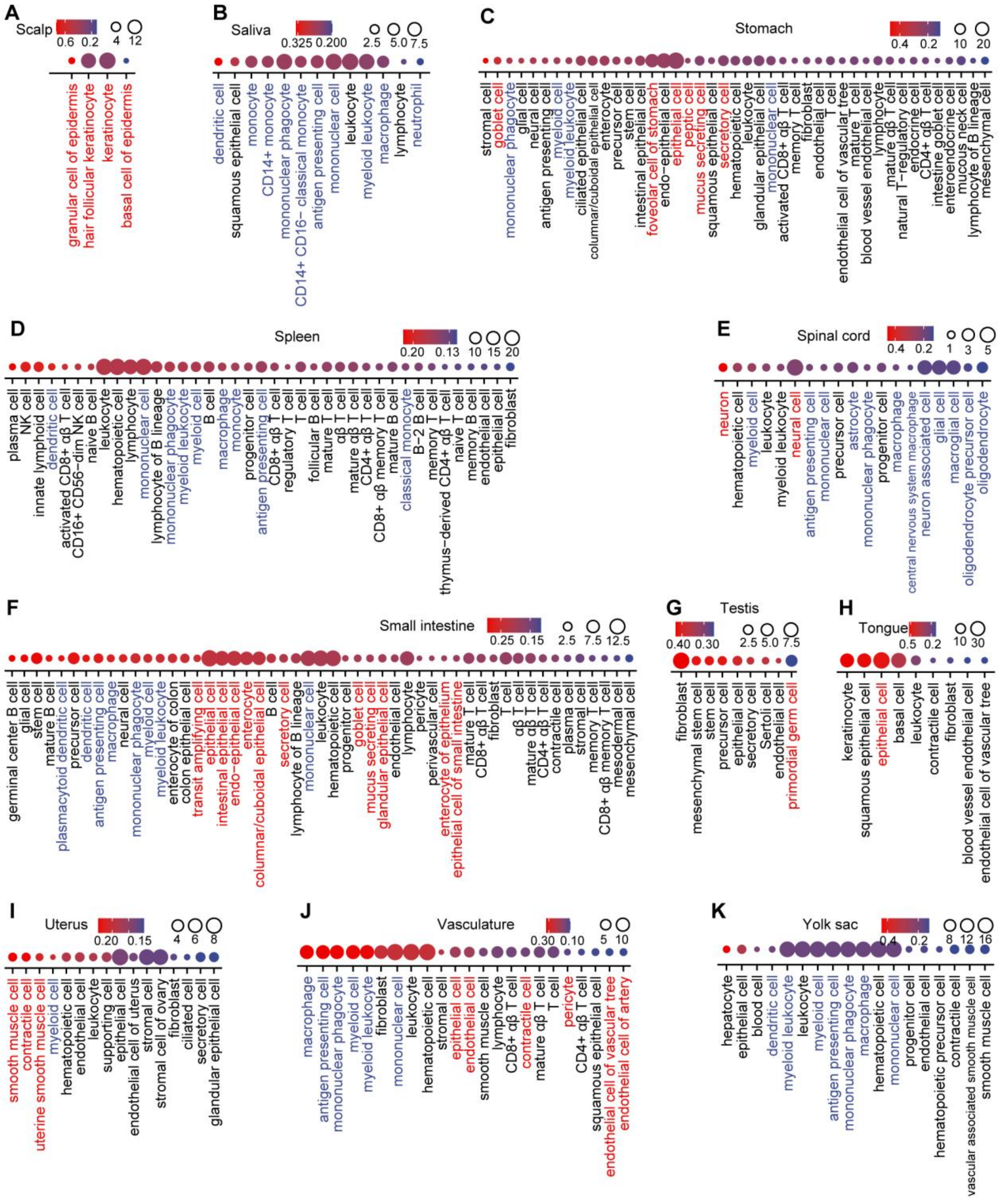
A
Scalp
0.6 0.2
4 12
granular cell of epidermis
hair follicular keratinocyte
keratinocyte
basal cell of epidermis
B
Saliva
0.325 0.200
2.5 5.0 7.5
dendritic cell
squamous epithelial cell
monocyte
CD14+ monocyte
mononuclear phagocyte
CD14+ CD16− classical monocyte
antigen presenting cell
mononuclear cell
leukocyte
myeloid leukocyte
macrophage
lymphocyte
neutrophil
C
Stomach
0.4 0.2
10 20
stromal cell
goblet cell
mononuclear phagocyte
glial cell
neural cell
antigen presenting cell
myeloid cell
myeloid leukocyte
ciliated epithelial cell
columnar/cuboidal epithelial cell
enterocyte
precursor cell
stem cell
intestinal epithelial cell
foveolar cell of stomach
endo−epithelial cell
epithelial cell
peptic cell
mucus secreting cell
secretory cell
squamous epithelial cell
hematopoietic cell
leukocyte
glandular epithelial cell
mononuclear cell
activated CD8+ αβ T cell
memory T cell
fibroblast
endothelial cell
T cell
endothelial cell of vascular tree
mature T cell
blood vessel endothelial cell
lymphocyte
mature αβ T cell
natural T−regulatory cell
endocrine cell
CD4+ αβ T cell
intestine goblet cell
enteroendocrine cell
mucous neck cell
lymphocyte of B lineage
mesenchymal cell
D
Spleen
0.20 0.13
10 15 20
plasma cell
NK cell
innate lymphoid cell
dendritic cell
activated CD8+ αβ T cell
CD16+ CD56−dim NK cell
naive B cell
leukocyte
hematopoietic cell
lymphocyte
mononuclear cell
lymphocyte of B lineage
mononuclear phagocyte
myeloid leukocyte
myeloid cell
B cell
macrophage
monocyte
progenitor cell
antigen presenting cell
CD8+ αβ T cell
regulatory T cell
follicular B cell
mature T cell
αβ T cell
mature αβ T cell
CD4+ αβ T cell
CD8+ αβ memory T cell
mature B cell
classical monocyte
B−2 B cell
memory T cell
thymus−derived CD4+ αβ T cell
naive T cell
memory B cell
endothelial cell
epithelial cell
fibroblast
E
Spinal cord
0.4 0.2
1 3 5
neuron
hematopoietic cell
myeloid cell
leukocyte
myeloid leukocyte
neural cell
antigen presenting cell
mononuclear cell
precursor cell
astrocyte
mononuclear phagocyte
progenitor cell
macrophage
central nervous system macrophage
neuron associated cell
glial cell
macroglial cell
oligodendrocyte precursor cell
oligodendrocyte
F
Small intestine
0.25 0.15
2.5 7.5 12.5
germinal center B cell
glial cell
stem cell
mature B cell
plasmacytoid dendritic cell
precursor cell
dendritic cell
antigen presenting cell
macrophage
neural cell
mononuclear phagocyte
myeloid cell
myeloid leukocyte
enterocyte of colon
colon epithelial cell
transit amplifying cell
epithelial cell
intestinal epithelial cell
endo−epithelial cell
enterocyte
columnar/cuboidal epithelial cell
B cell
secretory cell
lymphocyte of B lineage
mononuclear cell
leukocyte
hematopoietic cell
progenitor cell
goblet cell
mucus secreting cell
glandular epithelial cell
endothelial cell
lymphocyte
pericyte
perivascular cell
enterocyte of epithelium
epithelial cell of small intestine
mature T cell
CD8+ αβ T cell
fibroblast
T cell
αβ T cell
mature αβ T cell
CD4+ αβ T cell
contractile cell
plasma cell
stromal cell
memory T cell
CD8+ αβ memory T cell
mesodermal cell
mesenchymal cell
G
Testis
0.40 0.30
2.5 5.0 7.5
fibroblast
mesenchymal stem cell
stem cell
precursor cell
epithelial cell
secretory cell
Sertoli cell
endothelial cell
primordial germ cell
H
Tongue
0.5 0.2
10 30
keratinocyte
squamous epithelial cell
epithelial cell
basal cell
leukocyte
contractile cell
fibroblast
blood vessel endothelial cell
endothelial cell of vascular tree
I
Uterus
0.20 0.15
4 6 8
smooth muscle cell
contractile cell
uterine smooth muscle cell
myeloid cell
hematopoietic cell
endothelial cell
leukocyte
supporting cell
epithelial cell
endothelial cell of uterus
stromal cell
stromal cell of ovary
fibroblast
ciliated cell
secretory cell
glandular epithelial cell
J
Vasculature
0.30 0.10
5 10
macrophage
antigen presenting cell
mononuclear phagocyte
myeloid cell
myeloid leukocyte
fibroblast
mononuclear cell
leukocyte
hematopoietic cell
stromal cell
epithelial cell
endothelial cell
smooth muscle cell
lymphocyte
CD8+ αβ T cell
contractile cell
mature αβ T cell
T cell
pericyte
CD4+ αβ T cell
squamous epithelial cell
endothelial cell of vascular tree
endothelial cell of artery
K
Yolk sac
0.4 0.2
8 12 16
hepatocyte
epithelial cell
blood cell
dendritic cell
myeloid leukocyte
leukocyte
myeloid cell
antigen presenting cell
mononuclear phagocyte
macrophage
hematopoietic cell
mononuclear cell
progenitor cell
endothelial cell
hematopoietic precursor cell
contractile cell
vascular associated smooth muscle cell
smooth muscle cell

Fig. S4

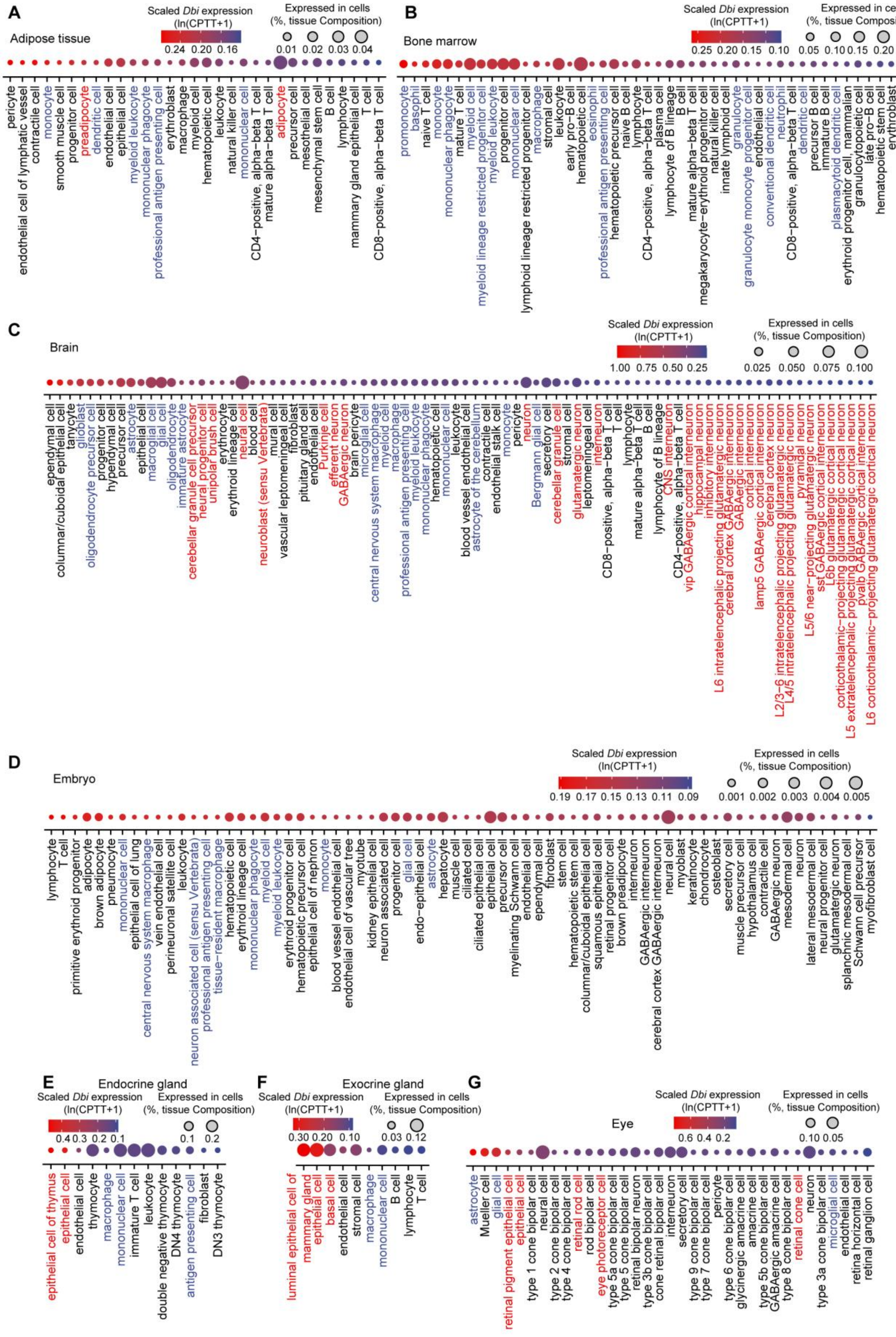
A
Adipose tissue
Scaled Dbi expression (ln(CPTT+1)
0.24 0.20 0.16
Expressed in cells (%, tissue Composition)
0.01 0.02 0.03 0.04
pericyte
endothelial cell of lymphatic vessel
contractile cell
monocyte
smooth muscle cell
progenitor cell
preadipocyte
dendritic cell
endothelial cell
epithelial cell
myeloid leukocyte
mononuclear phagocyte
professional antigen presenting cell
erythroblast
macrophage
myeloid cell
hematopoietic cell
leukocyte
natural killer cell
mononuclear cell
CD4−positive, alpha−beta T cell
mature alpha−beta T cell
adipocyte
precursor cell
mesothelial cell
mesenchymal stem cell
B cell
lymphocyte
mammary gland epithelial cell
T cell
CD8−positive, alpha−beta T cell
B
Bone marrow
Scaled Dbi expression (ln(CPTT+1)
0.25 0.20 0.15 0.10
Expressed in cells (%, tissue Composition)
0.05 0.10 0.15 0.20 0.25
promonocyte
basophil
naive T cell
monocyte
mononuclear phagocyte
mature T cell
myeloid cell
myeloid lineage restricted progenitor cell
myeloid leukocyte
progenitor cell
mononuclear cell
lymphoid lineage restricted progenitor cell
macrophage
stromal cell
leukocyte
early pro−B cell
hematopoietic cell
eosinophil
professional antigen presenting cell
hematopoietic precursor cell
naive B cell
lymphocyte
CD4−positive, alpha−beta T cell
plasma cell
lymphocyte of B lineage
B cell
mature alpha−beta T cell
megakaryocyte−erythroid progenitor cell
natural killer cell
innate lymphoid cell
granulocyte
granulocyte monocyte progenitor cell
endothelial cell
conventional dendritic cell
neutrophil
CD8−positive, alpha−beta T cell
dendritic cell
precursor B cell
immature B cell
plasmacytoid dendritic cell
erythroid progenitor cell, mammalian
granulocytopoietic cell
late pro−B cell
hematopoietic stem cell
erythroblast
erythroid lineage cell
proerythroblast
C
Brain
Scaled Dbi expression (ln(CPTT+1)
1.00 0.75 0.50 0.25
Expressed in cells (%, tissue Composition)
0.025 0.050 0.075 0.100
ependymal cell
columnar/cuboidal epithelial cell
tanycyte
glioblast
oligodendrocyte precursor cell
progenitor cell
hypendymal cell
precursor cell
astrocyte
epithelial cell
macroglial cell
glial cell
oligodendrocyte
immature astrocyte
cerebellar granule cell precursor
neural progenitor cell
unipolar brush cell
erythrocyte
erythroid lineage cell
neural cell
neuroblast (sensu Vertebrata)
blood cell
mural cell
vascular leptomeningeal cell
fibroblast
pituitary gland cell
endothelial cell
Purkinje cell
efferent neuron
GABAergic neuron
brain pericyte
microglial cell
central nervous system macrophage
myeloid cell
macrophage
professional antigen presenting cell
myeloid leukocyte
mononuclear phagocyte
hematopoietic cell
mononuclear cell
leukocyte
blood vessel endothelial cell
astrocyte of the cerebellum
contractile cell
endothelial stalk cell
monocyte
pericyte
neuron
Bergmann glial cell
secretory cell
cerebellar granule cell
stromal cell
glutamatergic neuron
leptomeningeal cell
interneuron
CD8−positive, alpha−beta T cell
T cell
lymphocyte
mature alpha−beta T cell
B cell
lymphocyte of B lineage
CNS interneuron
CD4−positive, alpha−beta T cell
vip GABAergic cortical interneuron
hippocampal neuron
inhibitory interneuron
L6 intratelencephalic projecting glutamatergic neuron
cerebral cortex GABAergic interneuron
GABAergic interneuron
cortical interneuron
lamp5 GABAergic cortical interneuron
cerebral cortex neuron
L2/3−6 intratelencephalic projecting glutamatergic neuron
L4/5 intratelencephalic projecting glutamatergic neuron
pyramidal neuron
L5/6 near−projecting glutamatergic neuron
sst GABAergic cortical interneuron
L6b glutamatergic cortical neuron
corticothalamic−projecting glutamatergic cortical neuron
L5 extratelencephalic projecting glutamatergic cortical neuron
pvalb GABAergic cortical interneuron
L6 corticothalamic−projecting glutamatergic cortical neuron
D
Embryo
Scaled Dbi expression (ln(CPTT+1)
0.19 0.17 0.15 0.13 0.11 0.09
Expressed in cells (%, tissue Composition)
0.001 0.002 0.003 0.004 0.005
lymphocyte
T cell
primitive erythroid progenitor
adipocyte
brown adipocyte
pneumocyte
mononuclear cell
epithelial cell of lung
central nervous system macrophage
vein endothelial cell
perineuronal satellite cell
leukocyte
neuron associated cell (sensu Vertebrata)
professional antigen presenting cell
tissue−resident macrophage
hematopoietic cell
erythroid lineage cell
mononuclear phagocyte
myeloid cell
myeloid leukocyte
erythroid progenitor cell
hematopoietic precursor cell
epithelial cell of nephron
monocyte
blood vessel endothelial cell
endothelial cell of vascular tree
myotube
kidney epithelial cell
neuron associated cell
progenitor cell
glial cell
endo−epithelial cell
astrocyte
hepatocyte
muscle cell
ciliated cell
ciliated epithelial cell
epithelial cell
precursor cell
myelinating Schwann cell
endothelial cell
ependymal cell
fibroblast
stem cell
hematopoietic stem cell
columnar/cuboidal epithelial cell
squamous epithelial cell
retinal progenitor cell
brown preadipocyte
interneuron
GABAergic interneuron
cerebral cortex GABAergic interneuron
neural cell
myoblast
keratinocyte
chondrocyte
osteoblast
secretory cell
muscle precursor cell
hypothalamus cell
contractile cell
GABAergic neuron
mesodermal cell
neuron
lateral mesodermal cell
neural progenitor cell
glutamatergic neuron
splanchnic mesodermal cell
Schwann cell precursor
myofibroblast
E
Endocrine gland
Scaled Dbi expression (ln(CPTT+1)
0.4 0.3 0.2 0.1
Expressed in cells (%, tissue Composition)
0.1 0.2
epithelial cell of thymus
epithelial cell
endothelial cell
thymocyte
macrophage
mononuclear cell
immature T cell
leukocyte
double negative thymocyte
DN4 thymocyte
antigen presenting cell
fibroblast
DN3 thymocyte
F
Exocrine gland
Scaled Dbi expression (ln(CPTT+1)
0.30 0.20 0.10
Expressed in cells (%, tissue Composition)
0.03 0.12
luminal epithelial cell of mammary gland
epithelial cell
basal cell
endothelial cell
stromal cell
macrophage
mononuclear cell
B cell
lymphocyte
T cell
G
Eye
Scaled Dbi expression (ln(CPTT+1)
0.6 0.4 0.2
Expressed in cells (%, tissue Composition)
0.10 0.05
astrocyte
Mueller cell
glial cell
retinal pigment epithelial cell
epithelial cell
type 1 cone bipolar cell
neural cell
type 2 cone bipolar cell
type 4 cone bipolar cell
retinal rod cell
rod bipolar cell
eye photoreceptor cell
type 5a cone bipolar cell
type 5 cone bipolar cell
retinal bipolar neuron
type 3b cone bipolar cell
cone retinal bipolar cell
interneuron
secretory cell
type 9 cone bipolar cell
type 7 cone bipolar cell
pericyte
type 6 cone bipolar cell
glycinergic amacrine cell
amacrine cell
type 5b cone bipolar cell
GABAergic amacrine cell
type 8 cone bipolar cell
retinal cone cell
neuron
type 3a cone bipolar cell
microglial cell
endothelial cell
retina horizontal cell
retinal ganglion cell

Fig. S5

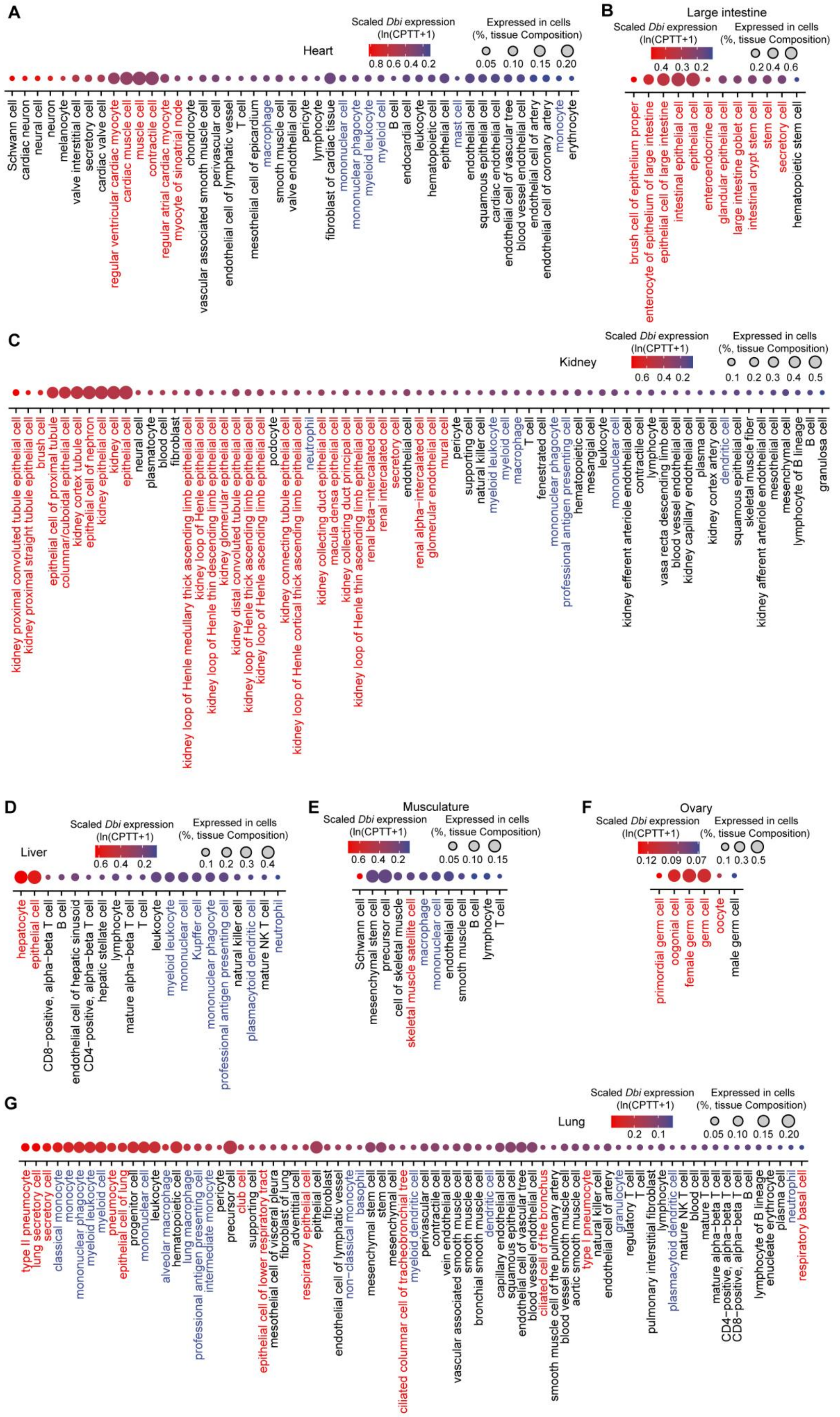
A
Scaled Dbi expression (ln(CPTT+1))
0.8 0.6 0.4 0.2
Expressed in cells (%, tissue Composition)
0.05 0.10 0.15 0.20
Heart
Schwann cell
cardiac neuron
neural cell
neuron
melanocyte
valve interstitial cell
secretory cell
cardiac valve cell
regular ventricular cardiac myocyte
cardiac muscle cell
muscle cell
contractile cell
regular atrial cardiac myocyte
myocyte of sinoatrial node
chondrocyte
vascular associated smooth muscle cell
perivascular cell
endothelial cell of lymphatic vessel
T cell
mesothelial cell of epicardium
macrophage
smooth muscle cell
valve endothelial cell
pericyte
lymphocyte
fibroblast of cardiac tissue
mononuclear cell
mononuclear phagocyte
myeloid leukocyte
myeloid cell
B cell
endocardial cell
leukocyte
hematopoietic cell
epithelial cell
mast cell
endothelial cell
squamous epithelial cell
cardiac endothelial cell
endothelial cell of vascular tree
blood vessel endothelial cell
endothelial cell of artery
endothelial cell of coronary artery
monocyte
erythrocyte
B
Large intestine
Scaled Dbi expression (ln(CPTT+1))
0.4 0.3 0.2
Expressed in cells (%, tissue Composition)
0.2 0.4 0.6
brush cell of epithelium proper
enterocyte of epithelium of large intestine
epithelial cell of large intestine
intestinal epithelial cell
epithelial cell
enteroendocrine cell
glandular epithelial cell
large intestine goblet cell
intestinal crypt stem cell
stem cell
secretory cell
hematopoietic stem cell
C
Scaled Dbi expression (ln(CPTT+1))
0.6 0.4 0.2
Expressed in cells (%, tissue Composition)
0.1 0.2 0.3 0.4 0.5
Kidney
kidney proximal convoluted tubule epithelial cell
kidney proximal straight tubule epithelial cell
brush cell
epithelial cell of proximal tubule
columnar/cuboidal epithelial cell
kidney cortex tubule cell
epithelial cell of nephron
kidney epithelial cell
kidney cell
epithelial cell
neural cell
plasmatocyte
blood cell
fibroblast
kidney loop of Henle medullary thick ascending limb epithelial cell
kidney loop of Henle epithelial cell
kidney loop of Henle thin descending limb epithelial cell
kidney glomerular epithelial cell
kidney distal convoluted tubule epithelial cell
kidney loop of Henle thick ascending limb epithelial cell
kidney loop of Henle ascending limb epithelial cell
podocyte
kidney connecting tubule epithelial cell
kidney loop of Henle cortical thick ascending limb epithelial cell
neutrophil
kidney collecting duct epithelial cell
macula densa epithelial cell
kidney collecting duct principal cell
kidney loop of Henle thin ascending limb epithelial cell
renal beta-intercalated cell
renal intercalated cell
secretory cell
endothelial cell
renal alpha-intercalated cell
glomerular endothelial cell
mural cell
pericyte
supporting cell
natural killer cell
myeloid leukocyte
myeloid cell
macrophage
T cell
fenestrated cell
mononuclear phagocyte
professional antigen presenting cell
hematopoietic cell
mesangial cell
leukocyte
mononuclear cell
kidney efferent arteriole endothelial cell
contractile cell
lymphocyte
vasa recta descending limb cell
blood vessel endothelial cell
kidney capillary endothelial cell
plasma cell
kidney cortex artery cell
dendritic cell
squamous epithelial cell
skeletal muscle fiber
kidney afferent arteriole endothelial cell
mesothelial cell
mesenchymal cell
lymphocyte of B lineage
B cell
granulosa cell
D
Scaled Dbi expression (ln(CPTT+1))
0.6 0.4 0.2
Expressed in cells (%, tissue Composition)
0.1 0.2 0.3 0.4
Liver
hepatocyte
epithelial cell
CD8−positive, alpha−beta T cell
B cell
endothelial cell of hepatic sinusoid
CD4−positive, alpha−beta T cell
hepatic stellate cell
lymphocyte
mature alpha−beta T cell
T cell
leukocyte
myeloid leukocyte
mononuclear cell
Kupffer cell
mononuclear phagocyte
professional antigen presenting cell
natural killer cell
plasmacytoid dendritic cell
mature NK T cell
neutrophil
E
Musculature
Scaled Dbi expression (ln(CPTT+1))
0.6 0.4 0.2
Expressed in cells (%, tissue Composition)
0.05 0.10 0.15
Schwann cell
mesenchymal stem cell
precursor cell
cell of skeletal muscle
skeletal muscle satellite cell
macrophage
mononuclear cell
endothelial cell
smooth muscle cell
B cell
lymphocyte
T cell
F
Ovary
Scaled Dbi expression (ln(CPTT+1))
0.12 0.09 0.07
Expressed in cells (%, tissue Composition)
0.1 0.3 0.5
primordial germ cell
oogonial cell
female germ cell
germ cell
oocyte
male germ cell
G
Scaled Dbi expression (ln(CPTT+1))
0.2 0.1
Expressed in cells (%, tissue Composition)
0.05 0.10 0.15 0.20
Lung
type II pneumocyte
lung secretory cell
secretory cell
classical monocyte
monocyte
mononuclear phagocyte
myeloid leukocyte
myeloid cell
pneumocyte
epithelial cell of lung
progenitor cell
mononuclear cell
leukocyte
alveolar macrophage
hematopoietic cell
lung macrophage
professional antigen presenting cell
intermediate monocyte
pericyte
precursor cell
club cell
supporting cell
epithelial cell of lower respiratory tract
mesothelial cell of visceral pleura
fibroblast of lung
adventitial cell
respiratory epithelial cell
epithelial cell
fibroblast
endothelial cell of lymphatic vessel
non−classical monocyte
basophil
mesenchymal stem cell
stem cell
mesenchymal cell
ciliated columnar cell of tracheobronchial tree
myeloid dendritic cell
perivascular cell
contractile cell
vein endothelial cell
vascular associated smooth muscle cell
smooth muscle cell
bronchial smooth muscle cell
dendritic cell
capillary endothelial cell
squamous epithelial cell
endothelial cell of vascular tree
blood vessel endothelial cell
ciliated cell of the bronchus
smooth muscle cell of the pulmonary artery
blood vessel smooth muscle cell
aortic smooth muscle cell
type I pneumocyte
natural killer cell
endothelial cell of artery
granulocyte
regulatory T cell
T cell
pulmonary interstitial fibroblast
lymphocyte
plasmacytoid dendritic cell
mature NK T cell
blood cell
mature T cell
mature alpha−beta T cell
CD4−positive, alpha−beta T cell
CD8−positive, alpha−beta T cell
B cell
lymphocyte of B lineage
enucleate erythrocyte
plasma cell
neutrophil
respiratory basal cell

Fig. S6

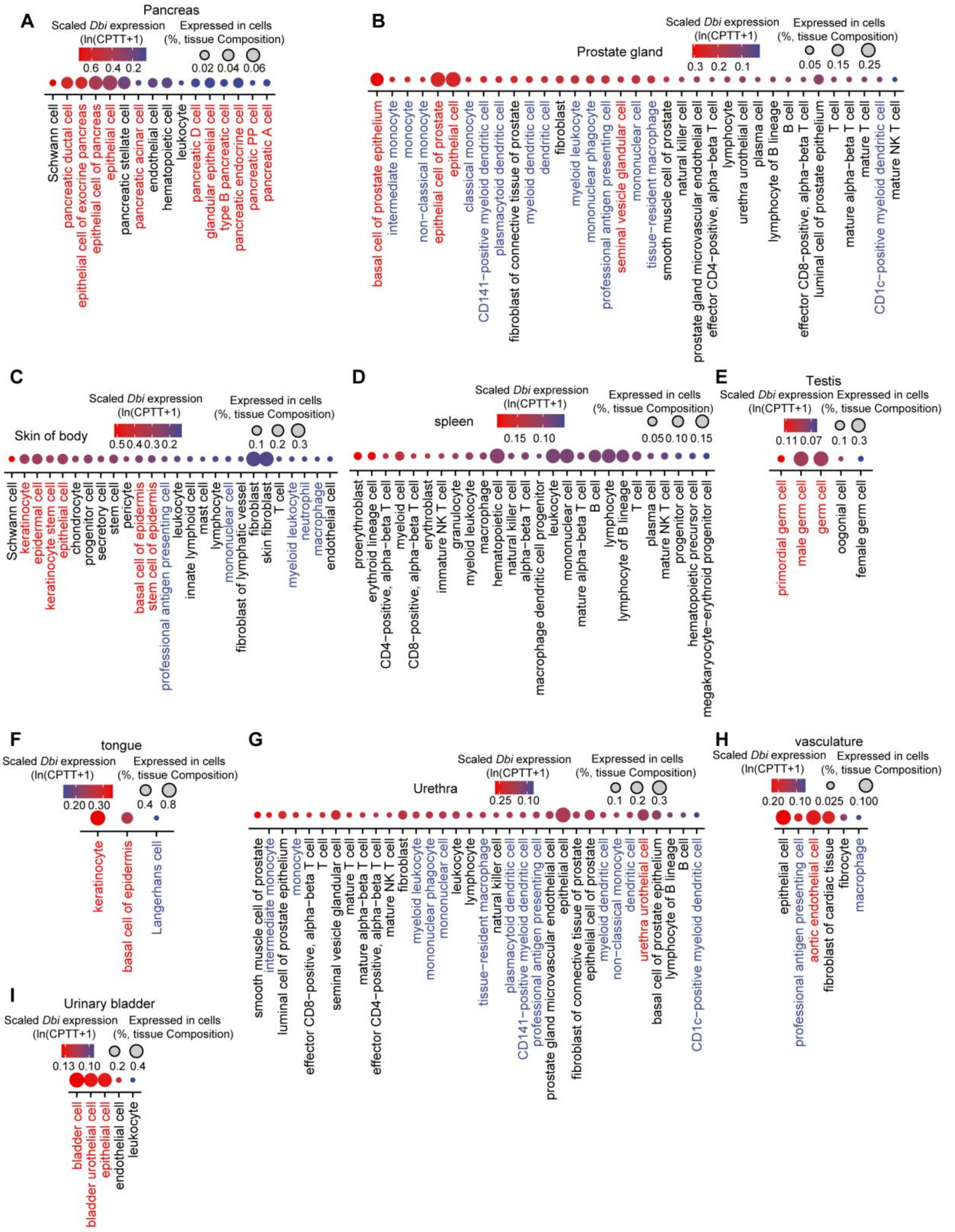
A
Pancreas
Scaled Dbi expression (ln(CPTT+1)
0.6 0.4 0.2
Expressed in cells (%, tissue Composition)
0.02 0.04 0.06
B
Prostate gland
C
Skin of body
D
spleen
E
Testis
F
tongue
G
Urethra
H
vasculature
I
Urinary bladder

Fig. S7

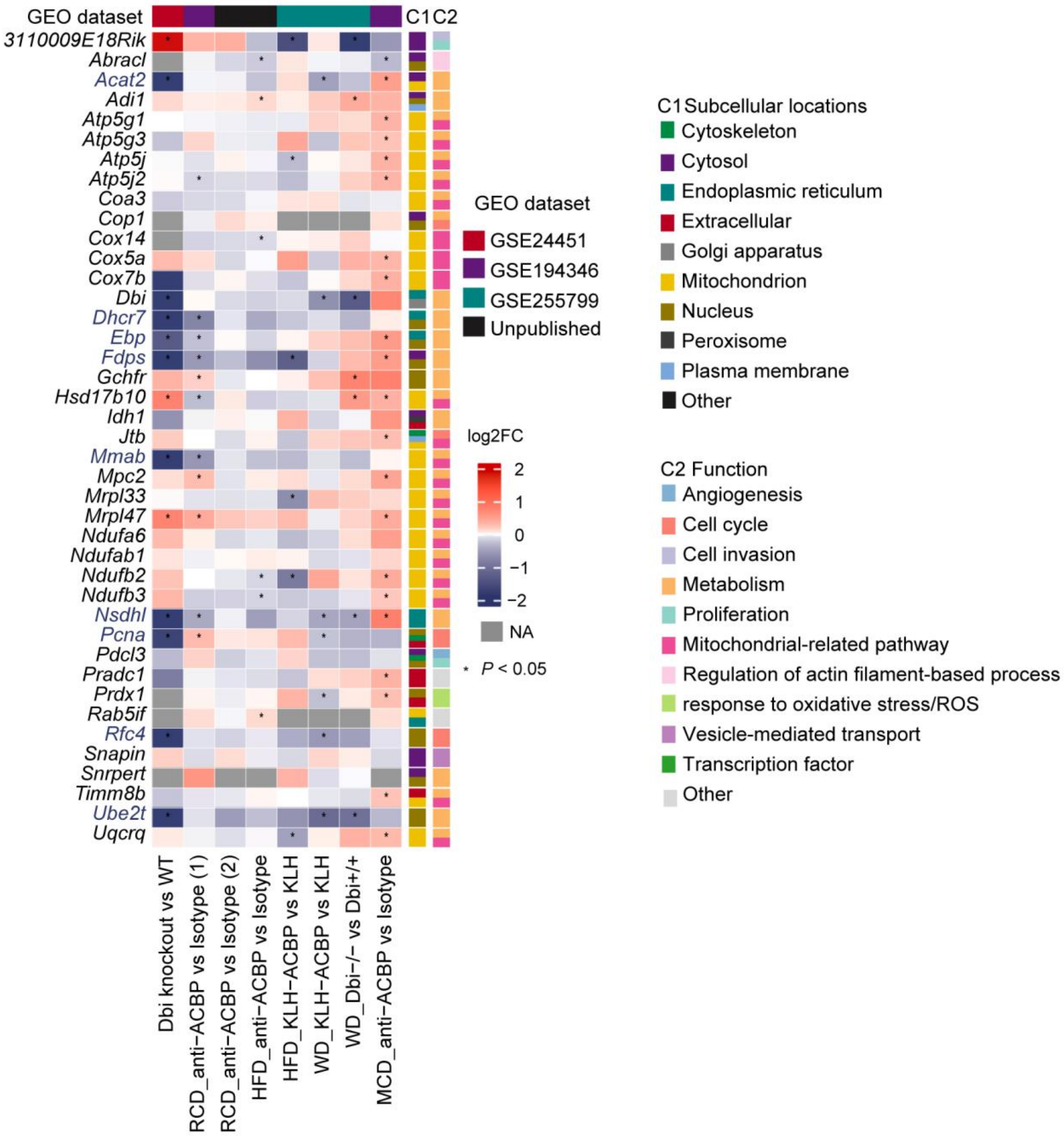
GEO dataset
C1
C2
3110009E18Rik
Abracl
Acat2
Adi1
Atp5g1
Atp5g3
Atp5j
Atp5j2
Coa3
Cop1
Cox14
Cox5a
Cox7b
Dbi
Dhcr7
Ebp
Fdps
Gchfr
Hsd17b10
Idh1
Jtb
Mmab
Mpc2
Mrpl33
Mrpl47
Ndufa6
Ndufab1
Ndufb2
Ndufb3
Nsdhl
Pcna
Pdcl3
Pradc1
Prdx1
Rab5if
Rfc4
Snapin
Snrpert
Timm8b
Ube2t
Uqcrq
Dbi knockout vs WT
RCD_anti−ACBP vs Isotype (1)
RCD_anti−ACBP vs Isotype (2)
HFD_anti−ACBP vs Isotype
HFD_KLH−ACBP vs KLH
WD_KLH−ACBP vs KLH
WD_Dbi−/− vs Dbi+/+
MCD_anti−ACBP vs Isotype
GEO dataset
GSE24451
GSE194346
GSE255799
Unpublished
log2FC
2
1
0
−1
−2
NA
* P < 0.05
C1 Subcellular locations
Cytoskeleton
Cytosol
Endoplasmic reticulum
Extracellular
Golgi apparatus
Mitochondrion
Nucleus
Peroxisome
Plasma membrane
Other
C2 Function
Angiogenesis
Cell cycle
Cell invasion
Metabolism
Proliferation
Mitochondrial-related pathway
Regulation of actin filament-based process
response to oxidative stress/ROS
Vesicle-mediated transport
Transcription factor
Other

Fig. S8

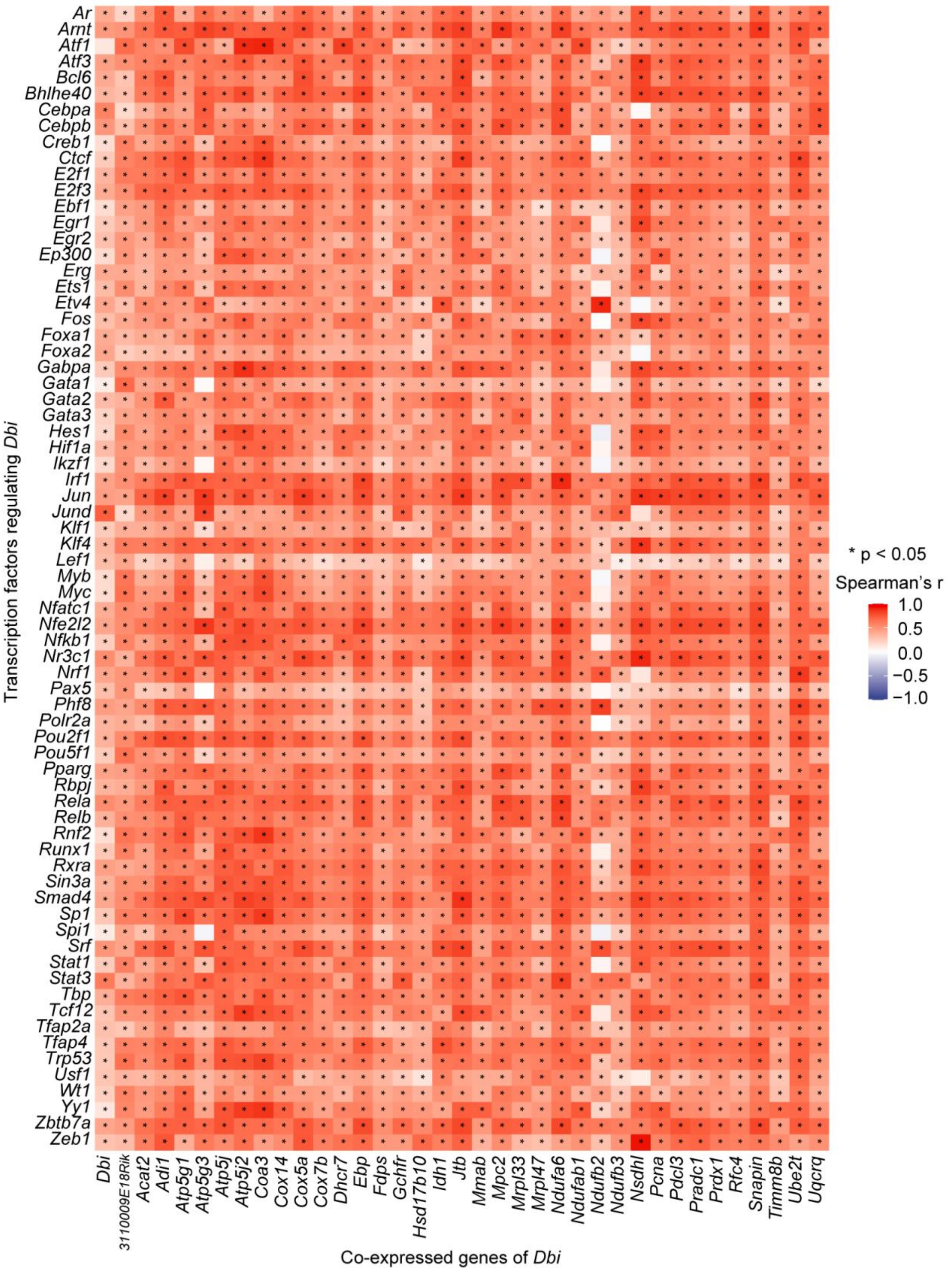
Transcription factors regulating Dbi
Ar
Arnt
Atf1
Atf3
Bcl6
Bhlhe40
Cebpa
Cebpb
Creb1
Ctcf
E2f1
E2f3
Ebf1
Egr1
Egr2
Ep300
Erg
Ets1
Etv4
Fos
Foxa1
Foxa2
Gabpa
Gata1
Gata2
Gata3
Hes1
Hif1a
Ikzf1
Irf1
Jun
Jund
Klf1
Klf4
Lef1
Myb
Myc
Nfatc1
Nfe2l2
Nfkb1
Nr3c1
Nrf1
Pax5
Phf8
Polr2a
Pou2f1
Pou5f1
Pparg
Rbpj
Rela
Relb
Rnf2
Runx1
Rxra
Sin3a
Smad4
Sp1
Spi1
Srf
Stat1
Stat3
Tbp
Tcf12
Tfap2a
Tfap4
Trp53
Usf1
Wt1
Yy1
Zbtb7a
Zeb1
Dbi
3110009E18Rik
Acat2
Adi1
Atp5g1
Atp5g3
Atp5j
Atp5j2
Coa3
Cox14
Cox5a
Cox7b
Dhcr7
Ebp
Fdps
Gchfr
Hsd17b10
Idh1
Jtb
Mmab
Mpc2
Mrpl33
Mrpl47
Ndufa6
Ndufab1
Ndufb2
Ndufb3
Nsdhl
Pcna
Pdcl3
Pradc1
Prdx1
Rfc4
Snapin
Timm8b
Ube2t
Uqcrq
Co-expressed genes of Dbi
* p < 0.05
Spearman's r
1.0
0.5
0.0
−0.5
−1.0

Fig. S9

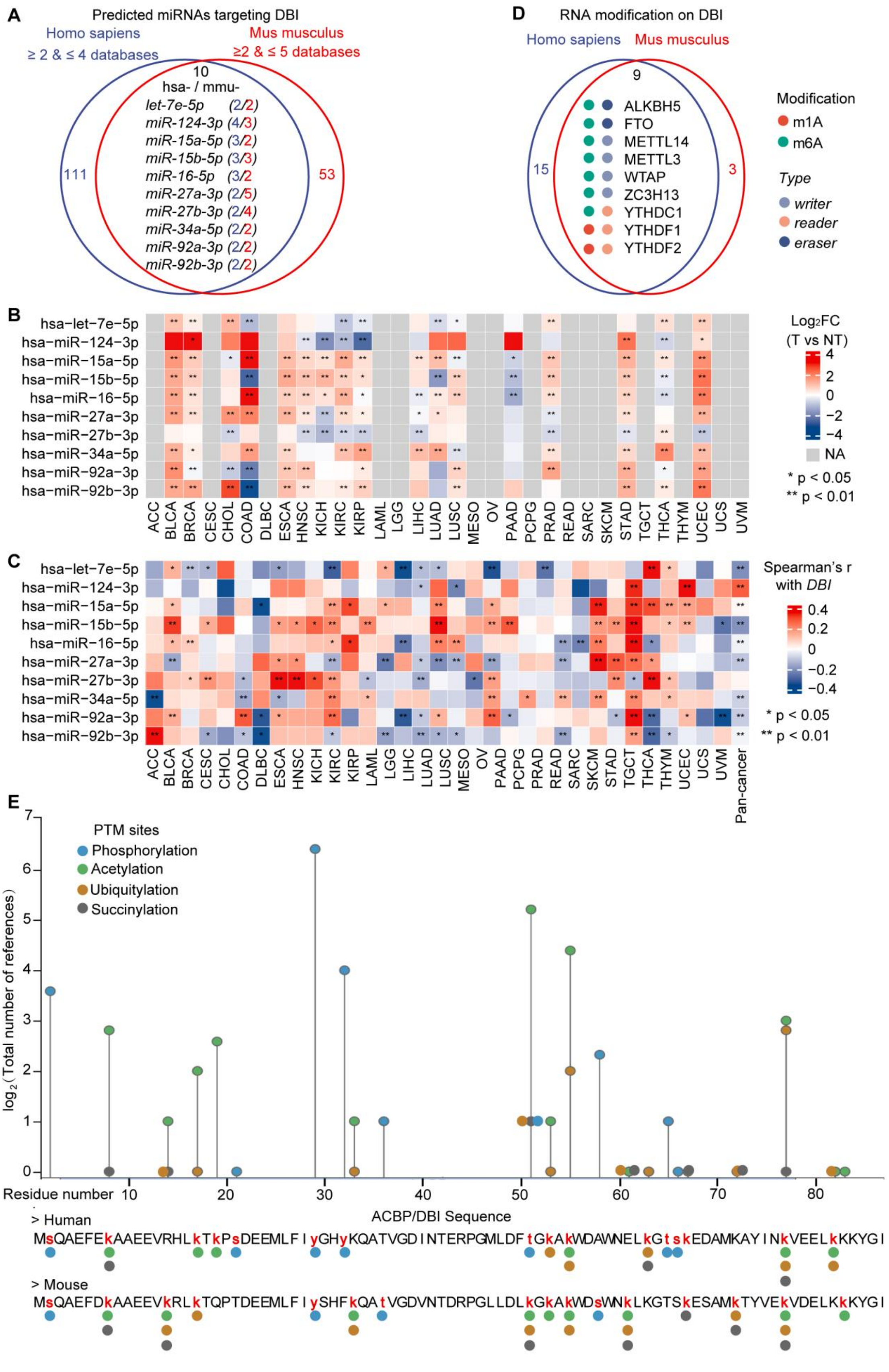
A
Predicted miRNAs targeting DBI
Homo sapiens
≥ 2 & ≤ 4 databases
Mus musculus
≥2 & ≤ 5 databases
10
hsa- / mmu-
let-7e-5p (2/2)
miR-124-3p (4/3)
miR-15a-5p (3/2)
miR-15b-5p (3/3)
miR-16-5p (3/2
miR-27a-3p (2/5)
miR-27b-3p (2/4)
miR-34a-5p (2/2)
miR-92a-3p (2/2)
miR-92b-3p (2/2)
111
53
D
RNA modification on DBI
Homo sapiens
Mus musculus
9
15
3
ALKBH5
FTO
METTL14
METTL3
WTAP
ZC3H13
YTHDC1
YTHDF1
YTHDF2
Modification
m1A
m6A
Type
writer
reader
eraser
B
Log2FC (T vs NT)
NA
* p < 0.05
** p < 0.01
C
Spearman's r with DBI
* p < 0.05
** p < 0.01
Pan-cancer
E
PTM sites
Phosphorylation
Acetylation
Ubiquitylation
Succinylation
log2 (Total number of references)
Residue number
ACBP/DBI Sequence
> Human
MsQAEFEkAAEEVRHLkTkPsDEEMLFIyGHyKQATVGDINTERPGMLDFtGkAkWDAWNELkGtskEDAMKAYINkVEELkKKYGI
> Mouse
MsQAEFDkAAEEVkRLkTQPTDEEMLFIySHFkQAtVGDVNTDRPGLLDLkGkAkWDsWNkLKGTSkESAMkTYVEkVDELKkKYGI

Fig. S10
