# Supplementary material for: Atlas of expression of acyl CoA binding protein/diazepam binding inhibitor (ACBP/DBI) in human and mouse
Source: Cell Death Dis. 2025 Feb 26;16(1):134. doi: 10.1038/s41419-025-07447-w (PMC11865319; doi:10.1038/s41419-025-07447-w)

Original data

Figure 1A

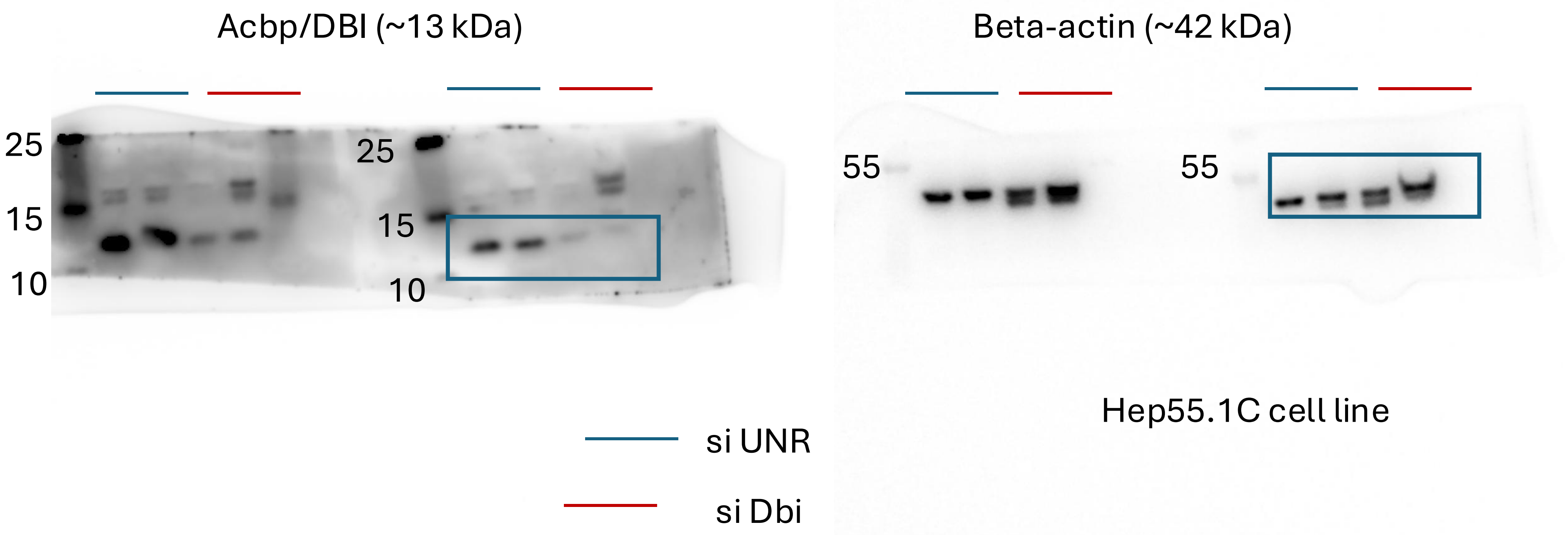

Figure 1B
Mice Livers

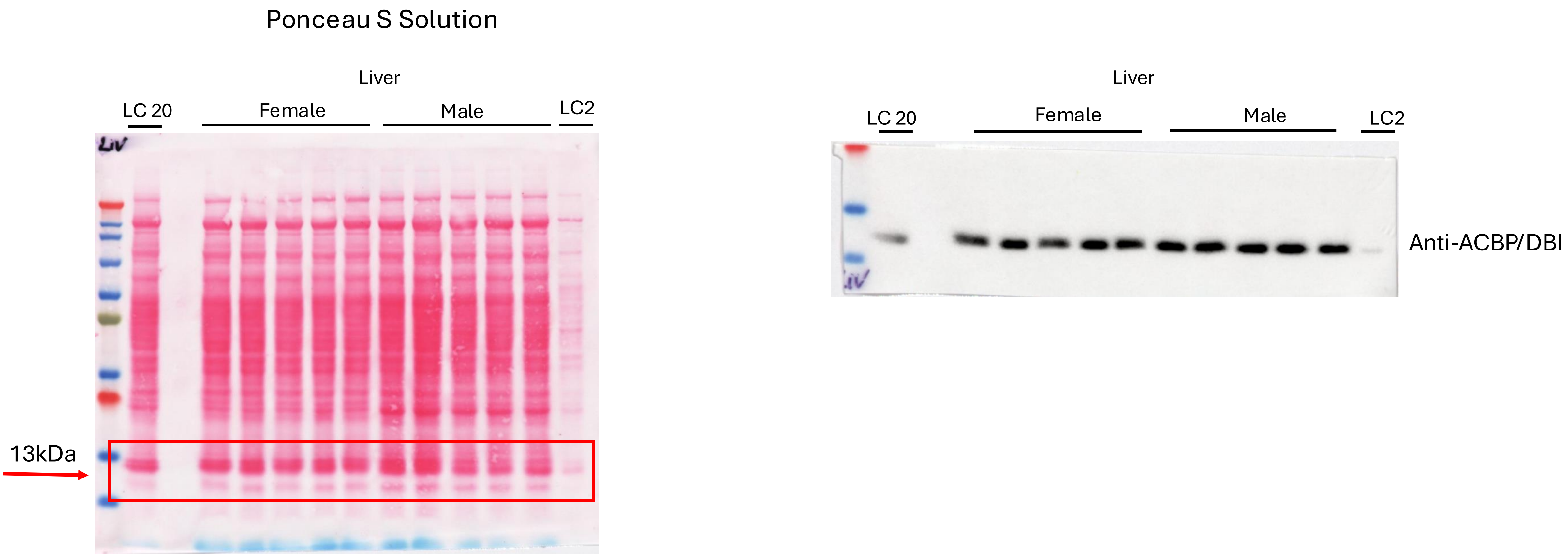

Figure S1

1. Mice Lungs

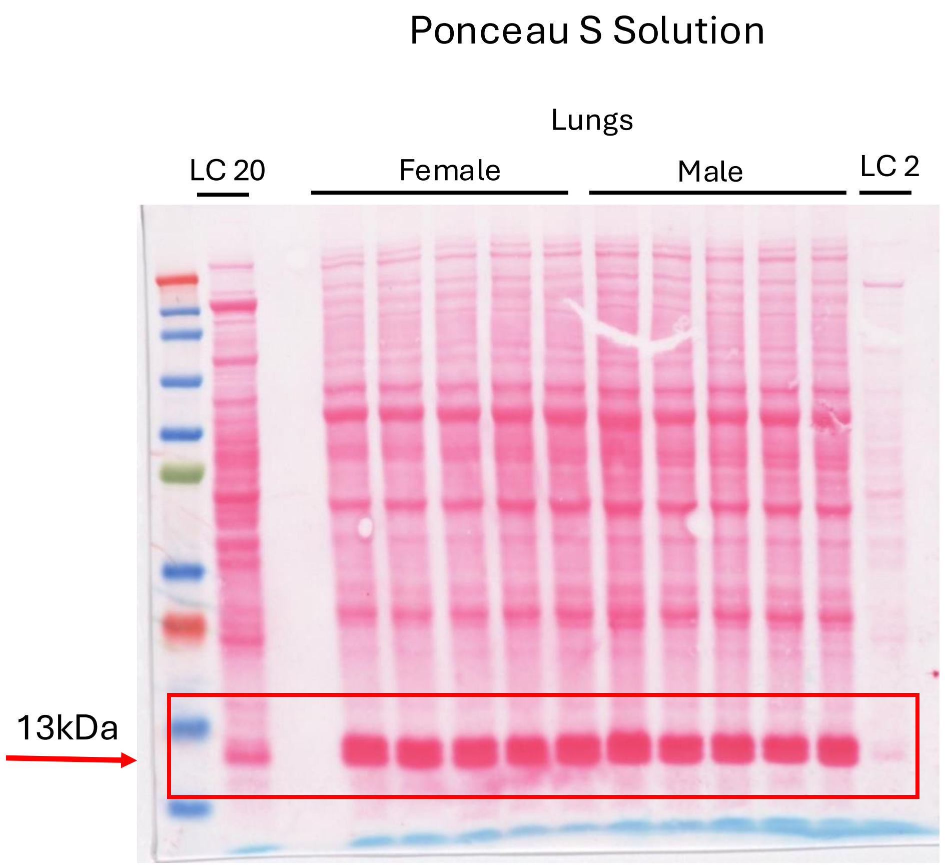

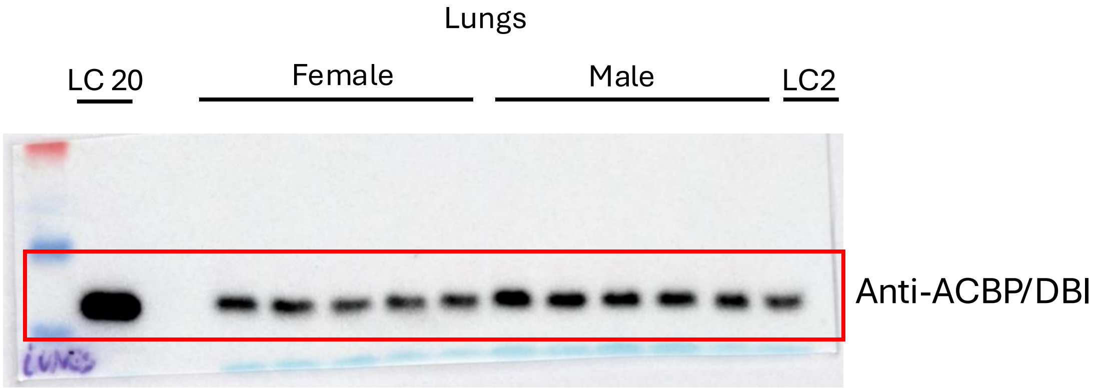

Figure S1

2. Mice Hearts

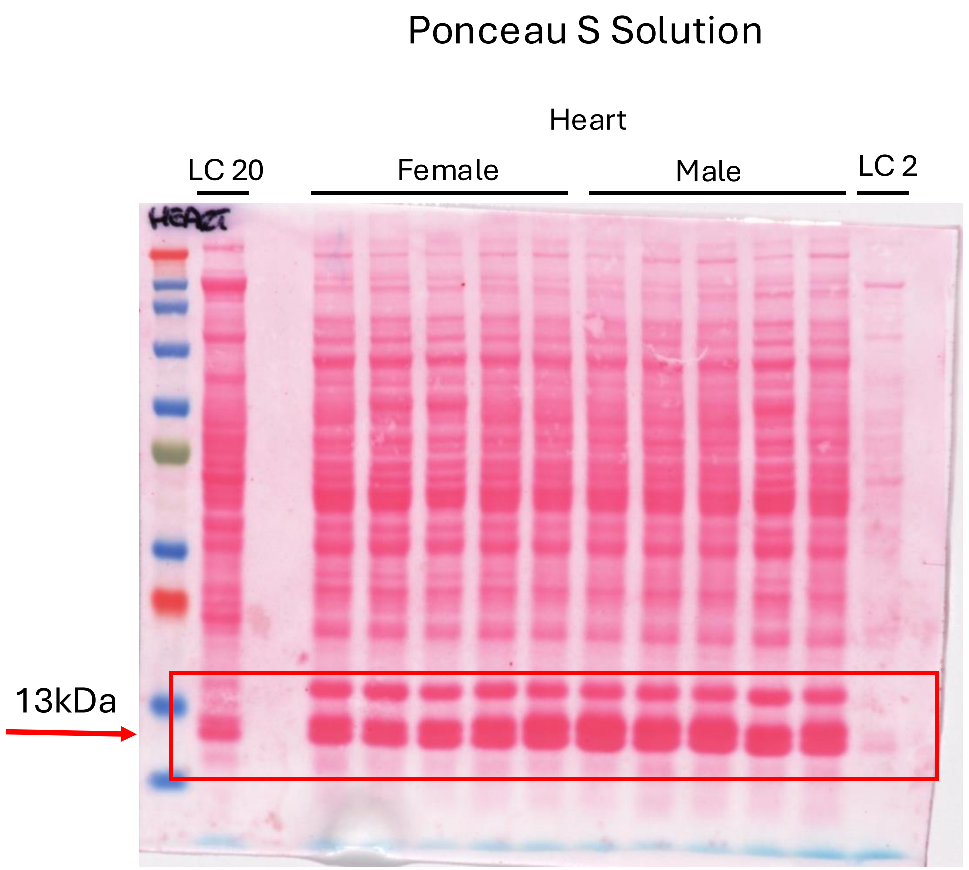

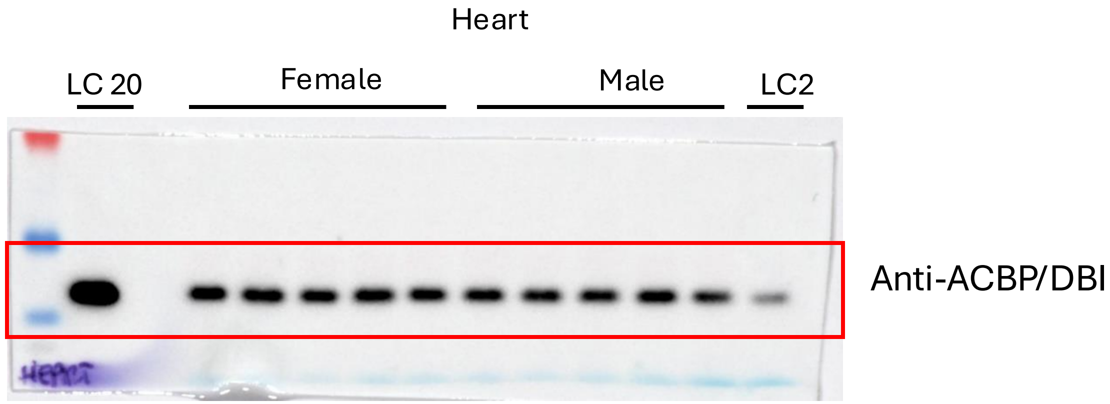

Figure S1

3. Mice Kidney

Ponceau S Solution

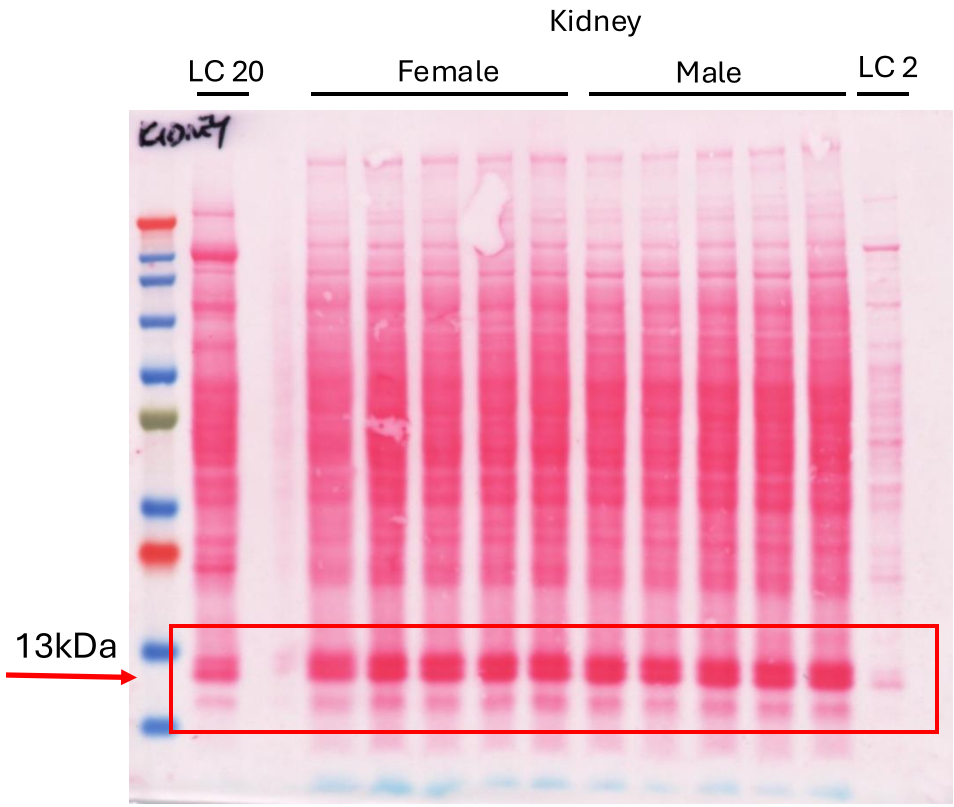

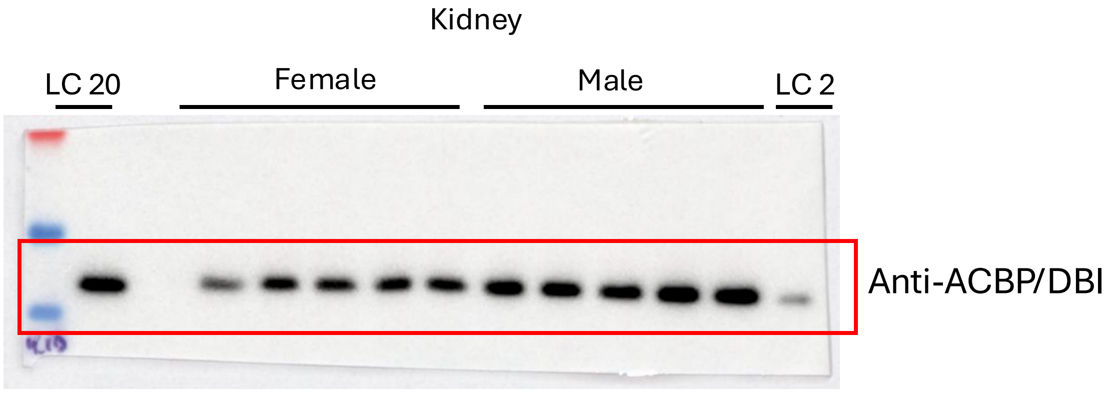

Figure S1

4. Mice Pancreas

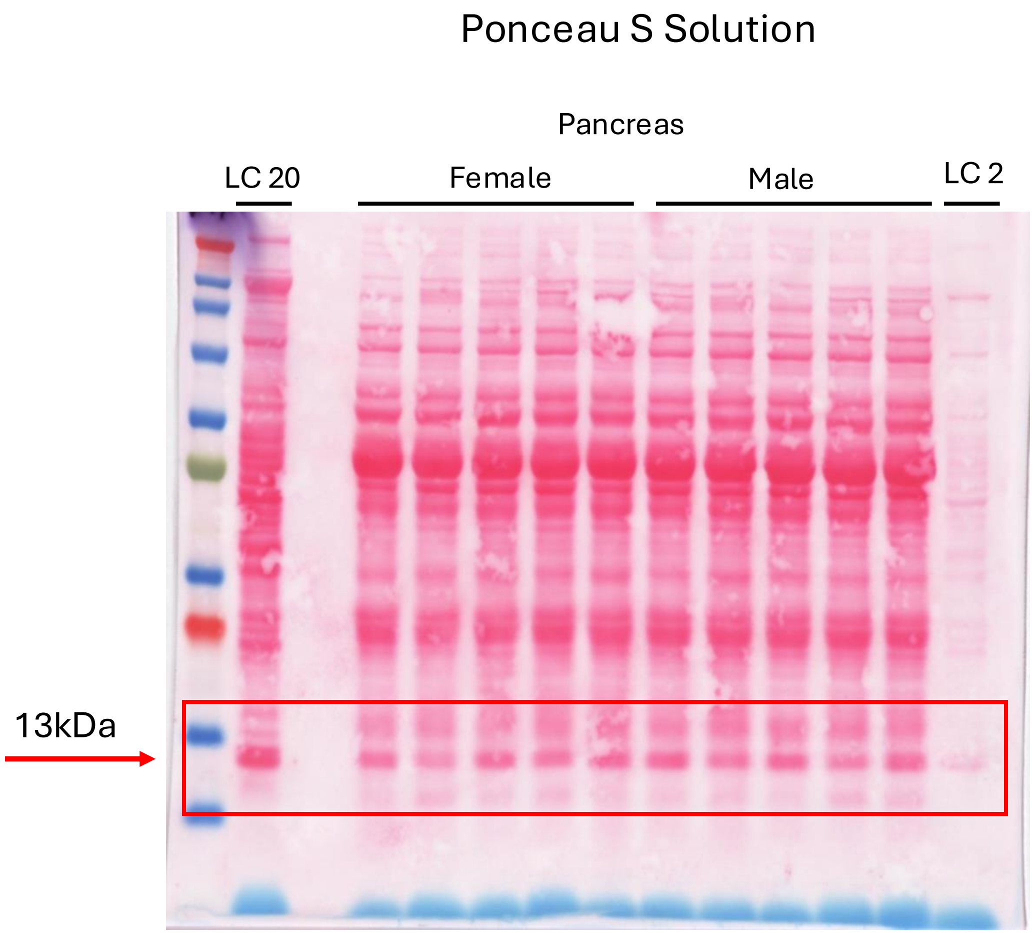

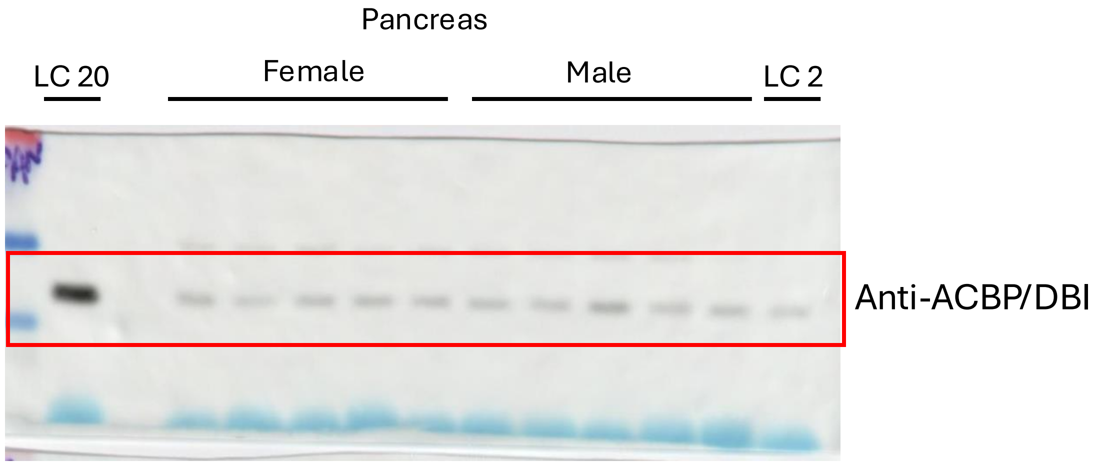

Figure S1

5. Mice Salivary Glands

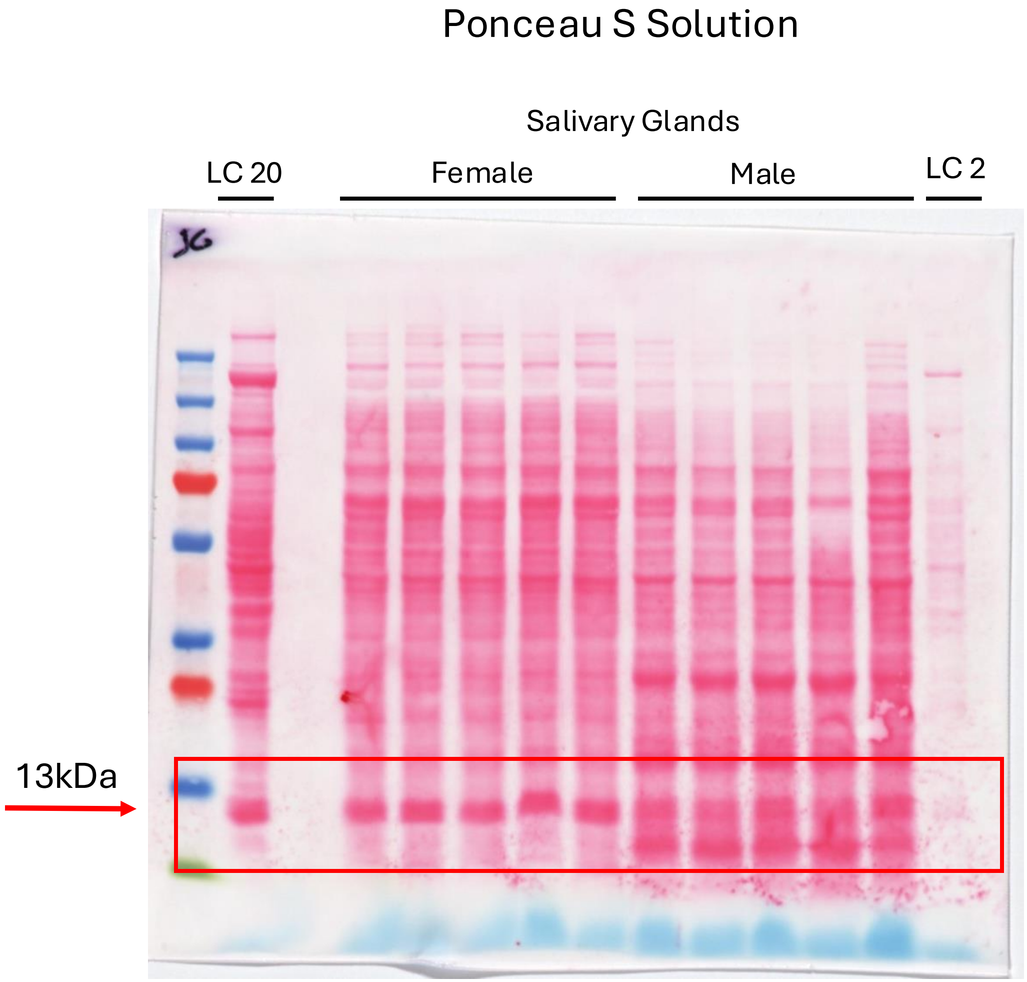

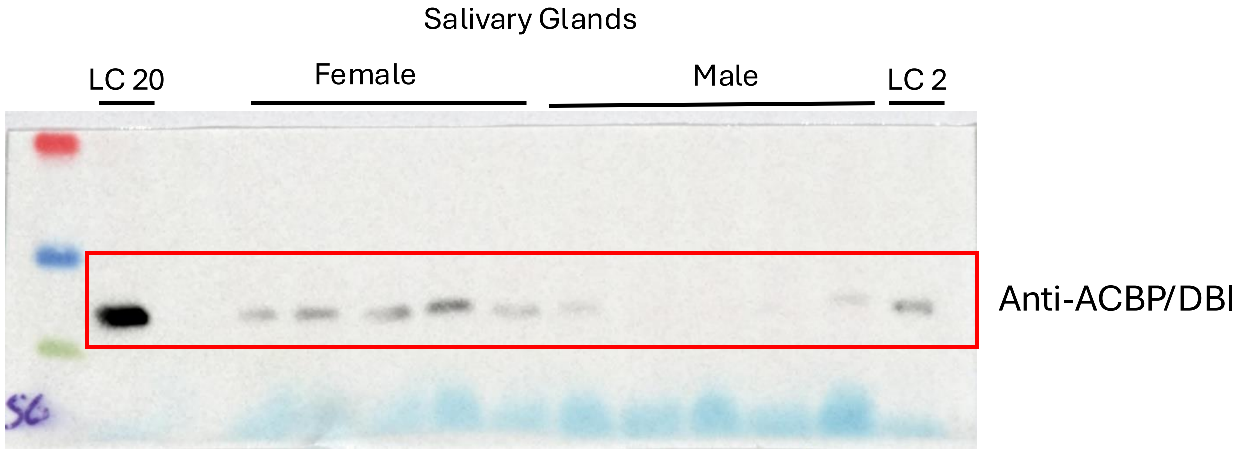

Figure S1

6. Mice Muscle

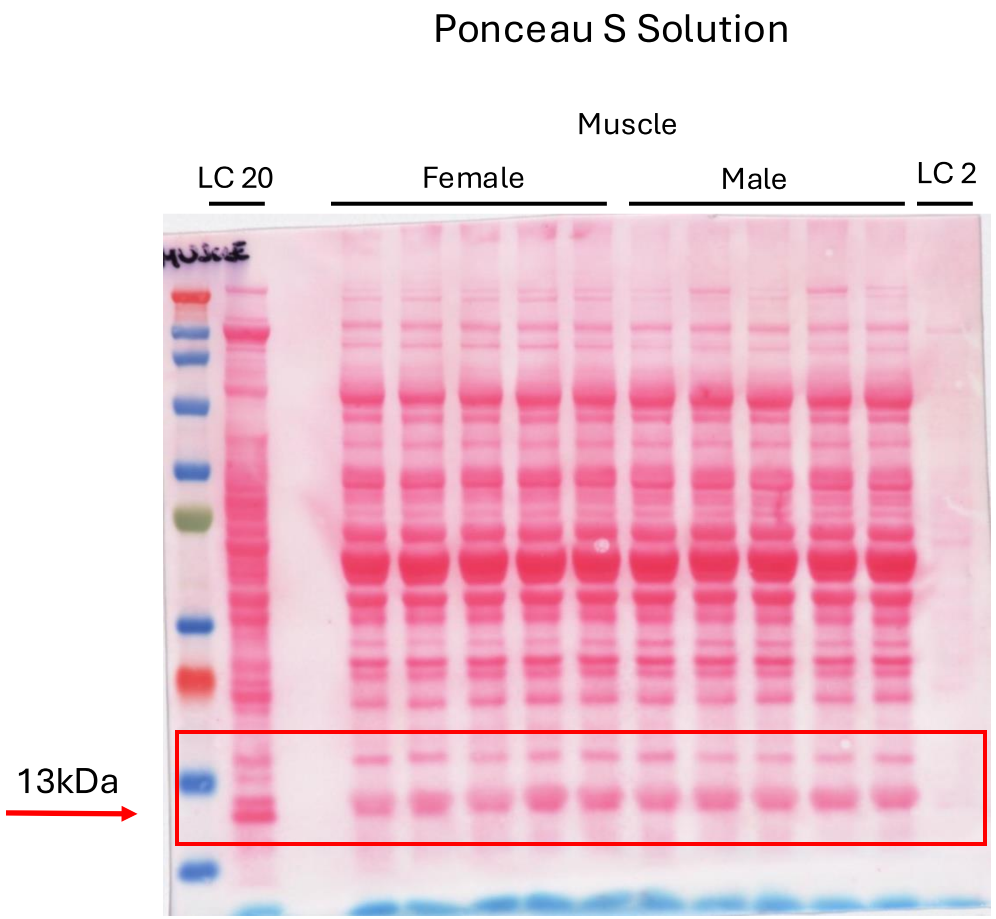

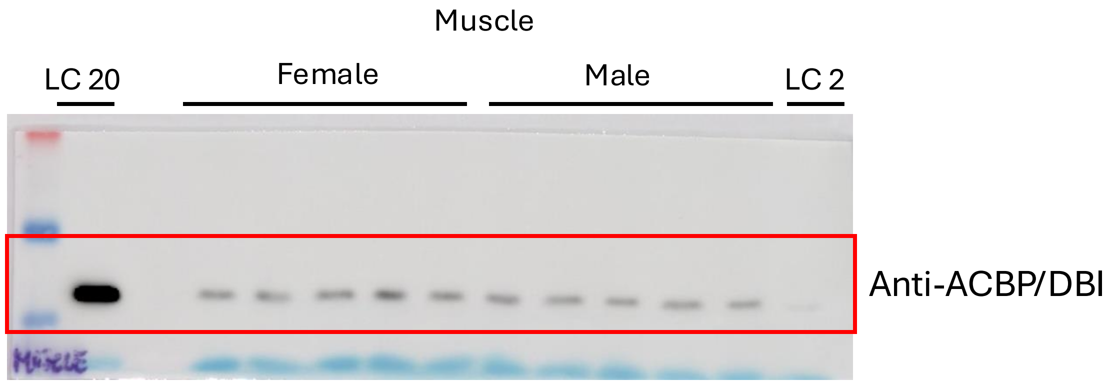

Figure S1
7. Mice iWAT

Ponceau S Solution

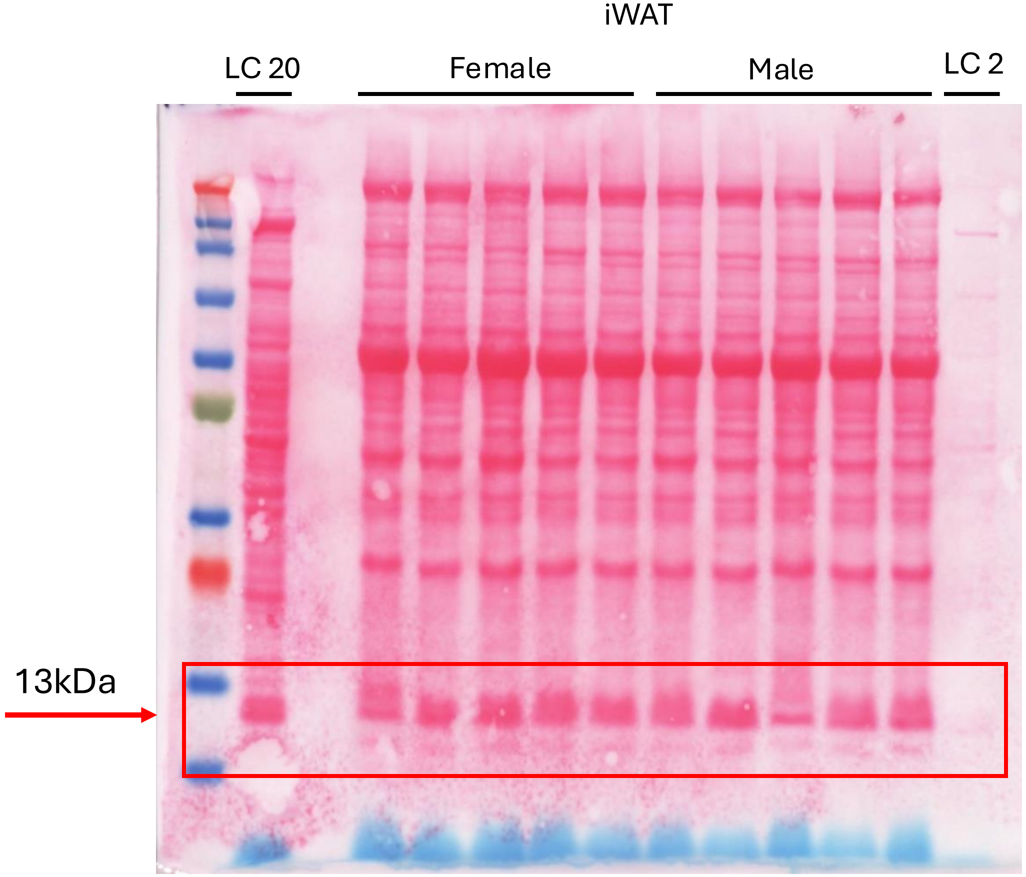

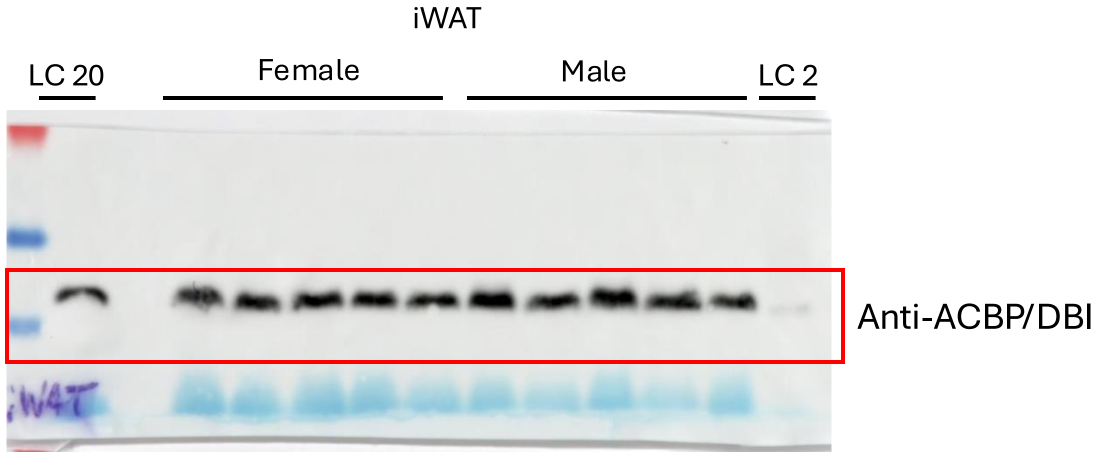

Figure S1

8. Mice BAT

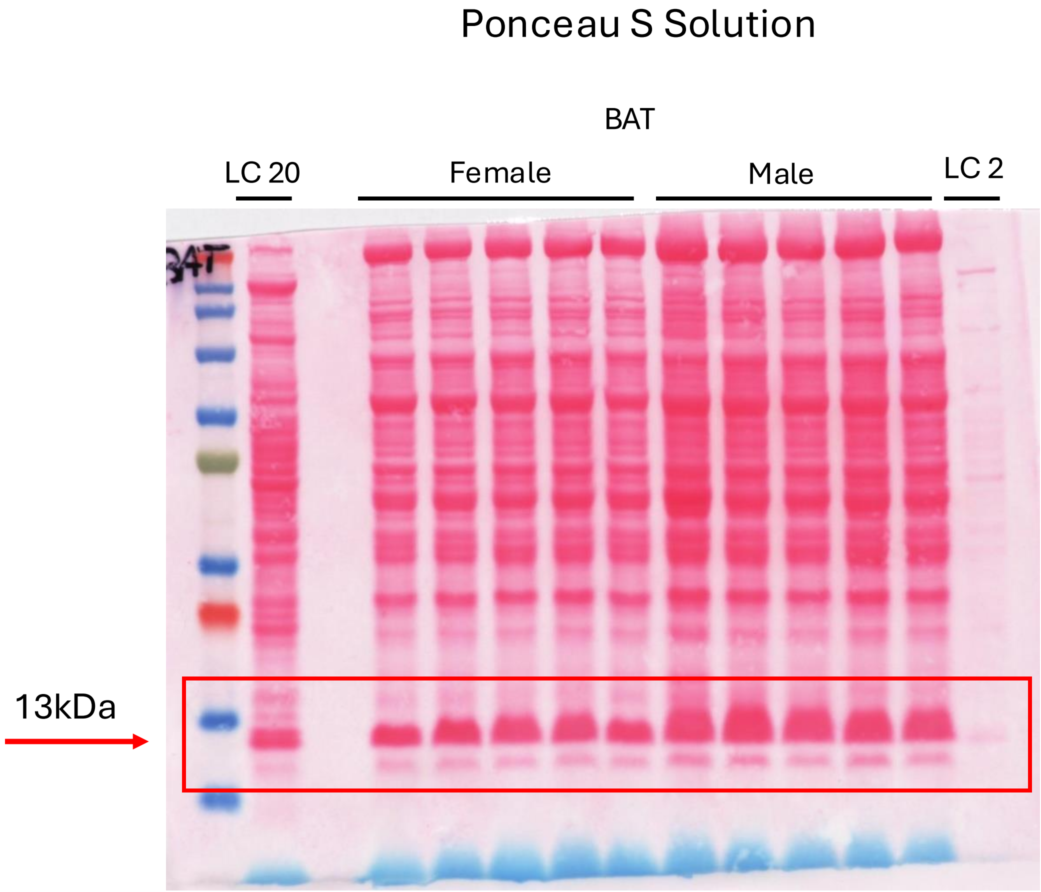

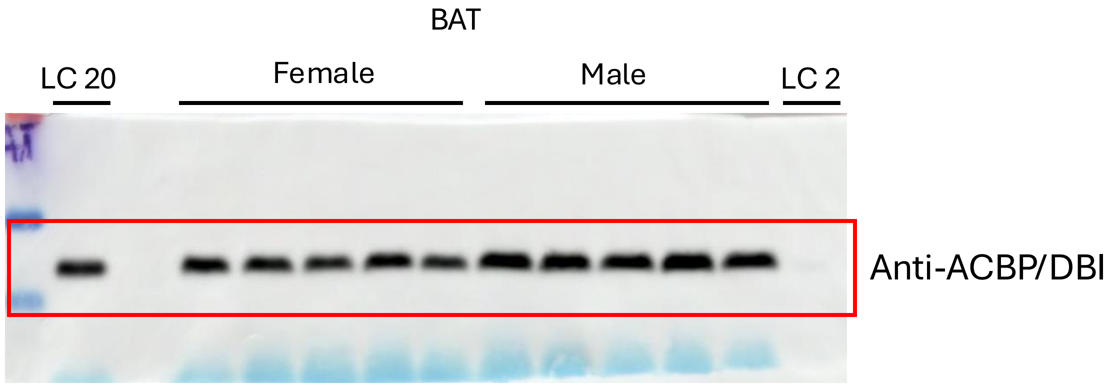

Figure S1

9. Mice Spleen

Ponceau S Solution

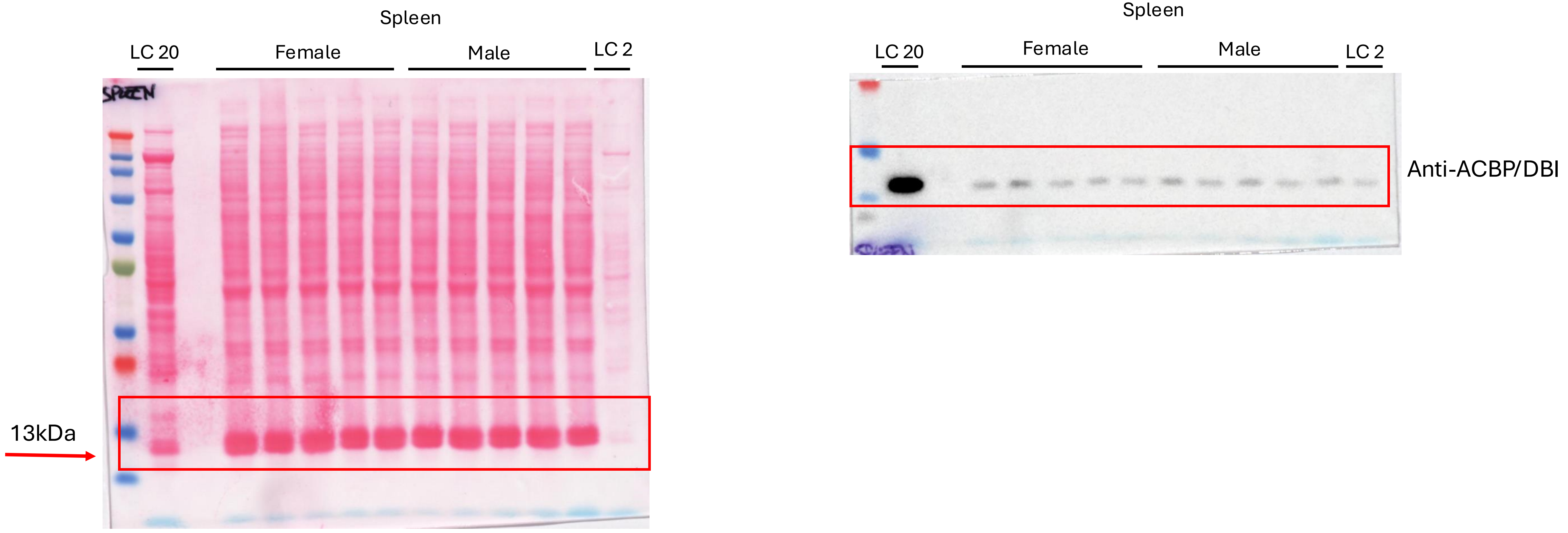

Figure S1
10. Mice Thymus

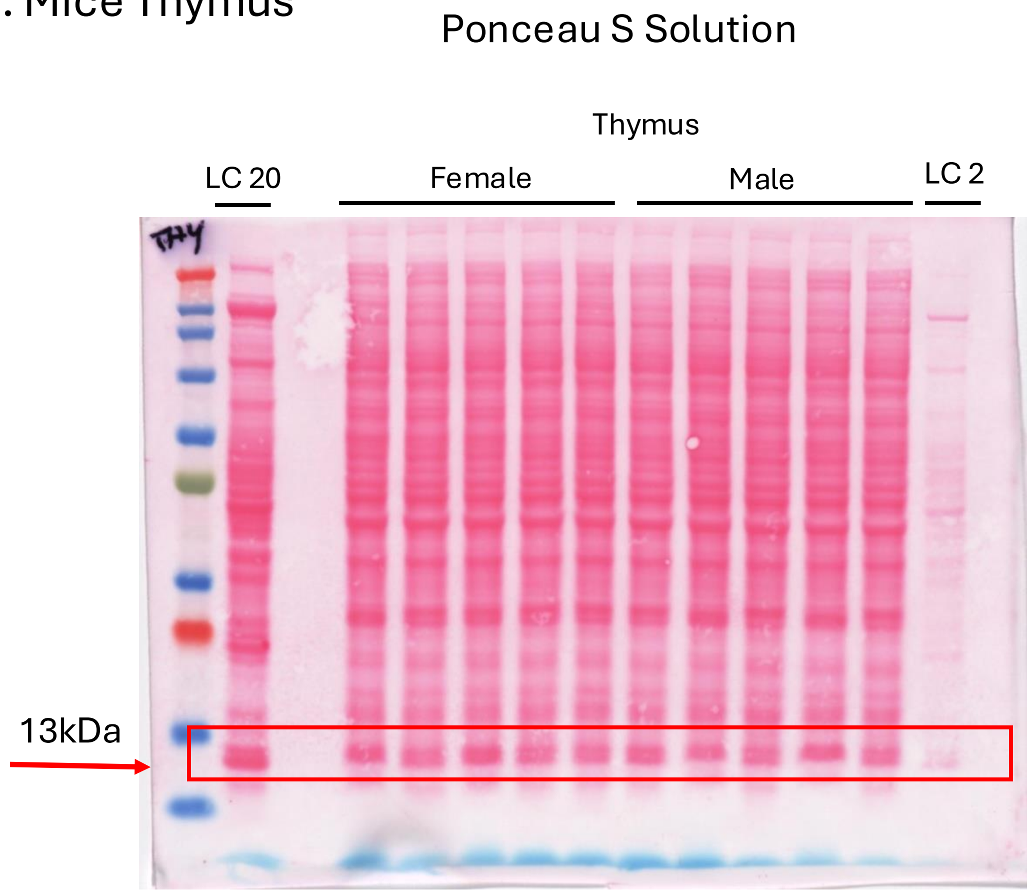

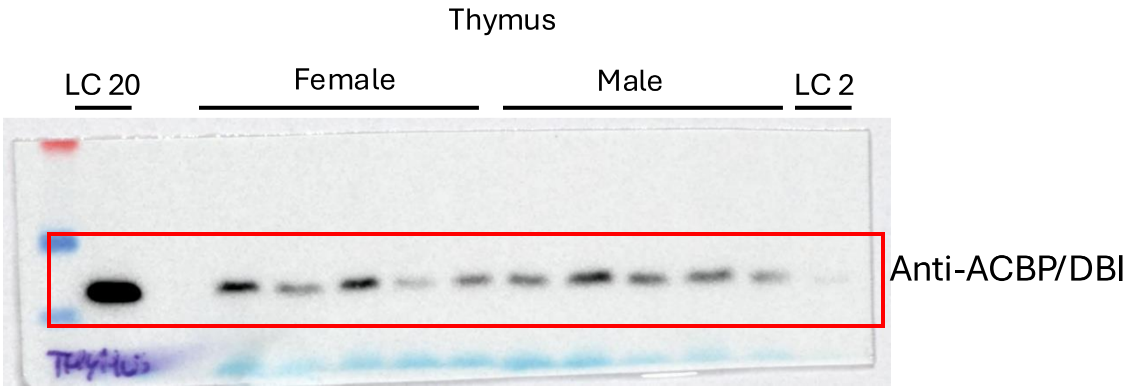

Figure S1

11. Mice Lymph nodes

Ponceau S Solution

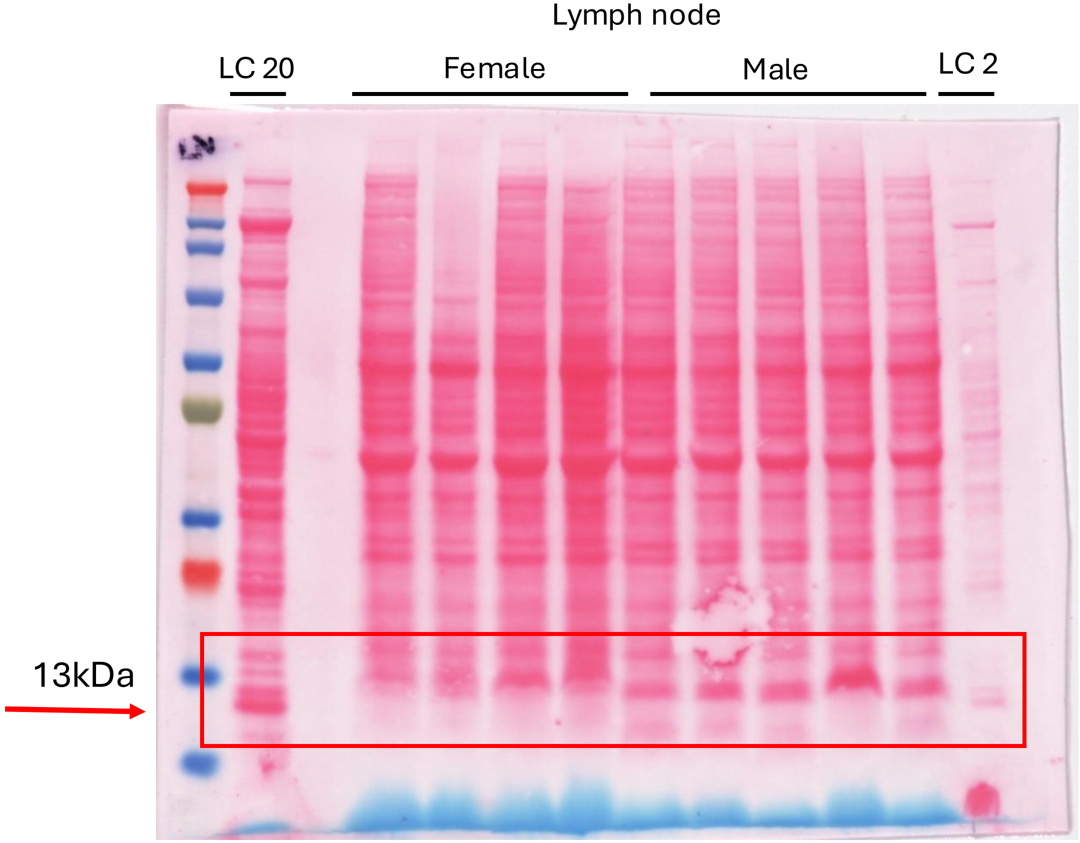

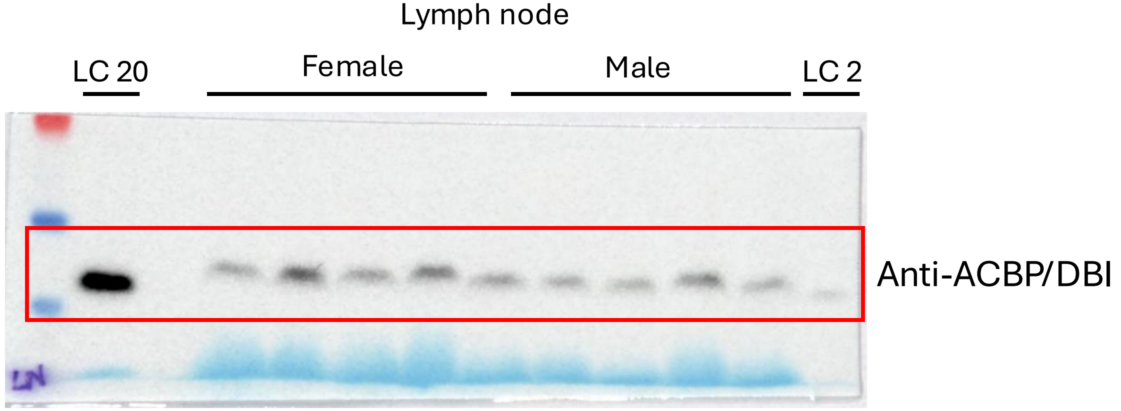

Figure S1

12. Mice Cerebellum

Ponceau S Solution

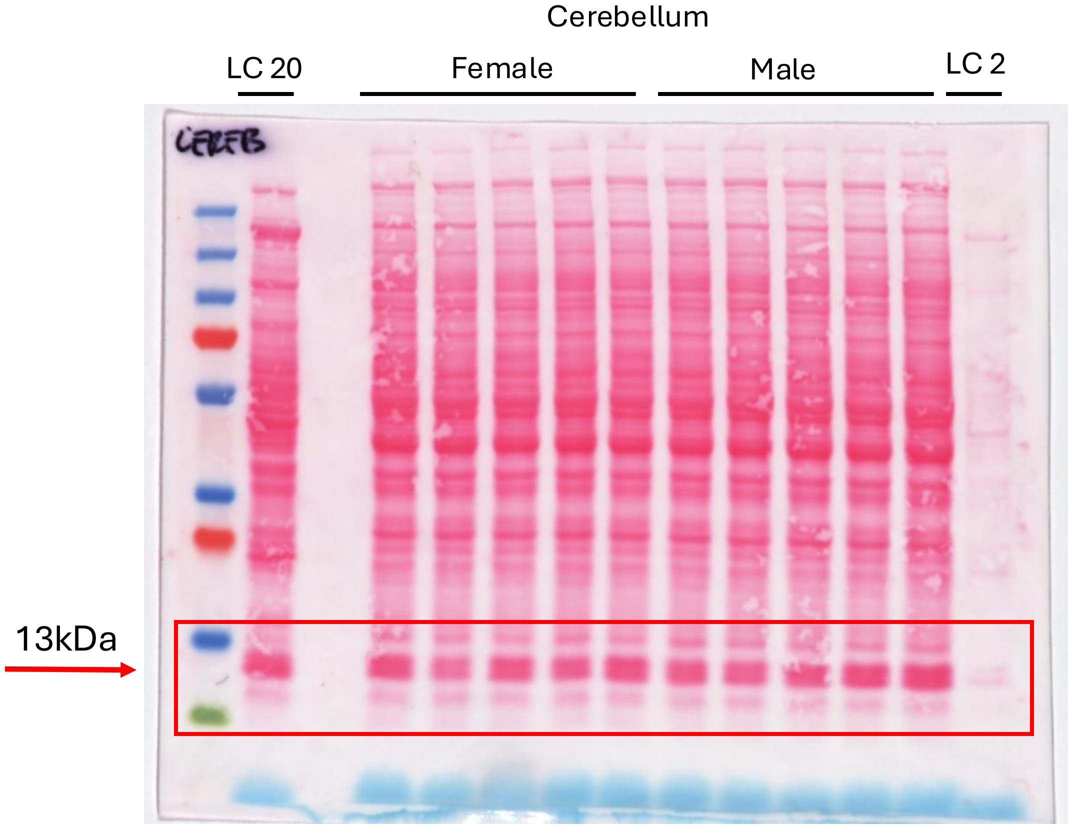

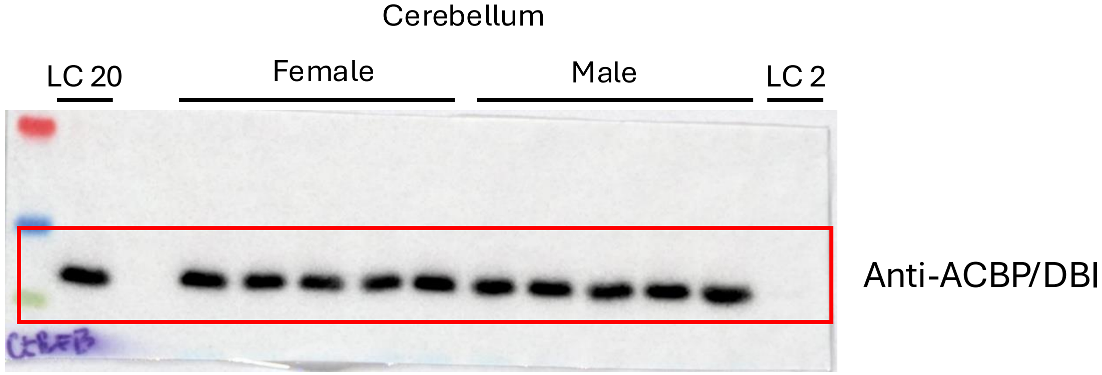

Figure S1
13. Mice Medulla

Ponceau S Solution

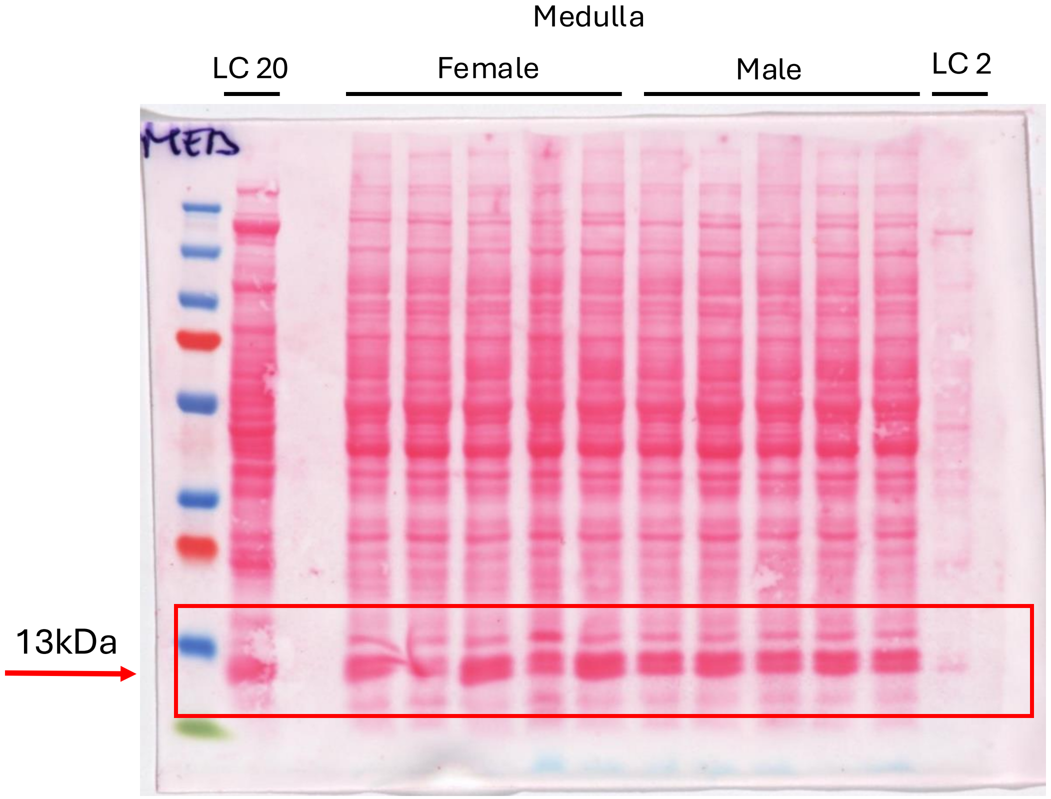

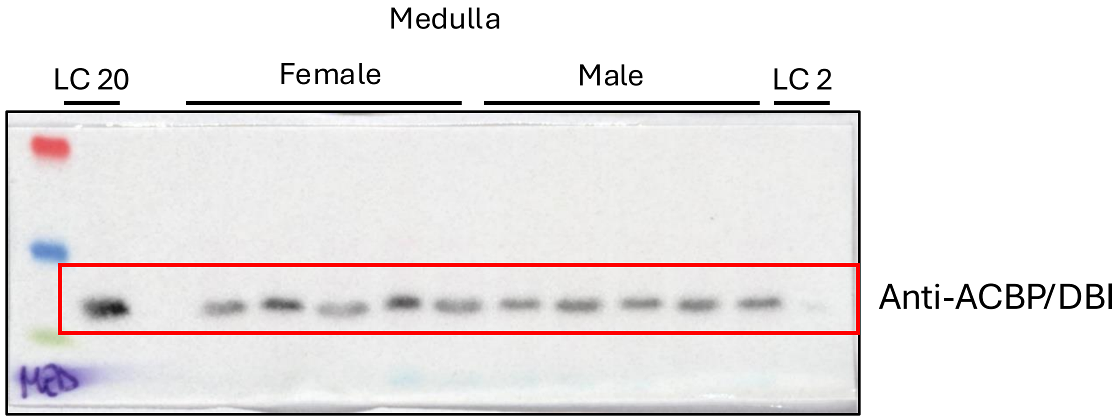

Figure S1

14. Mice Hippocampus

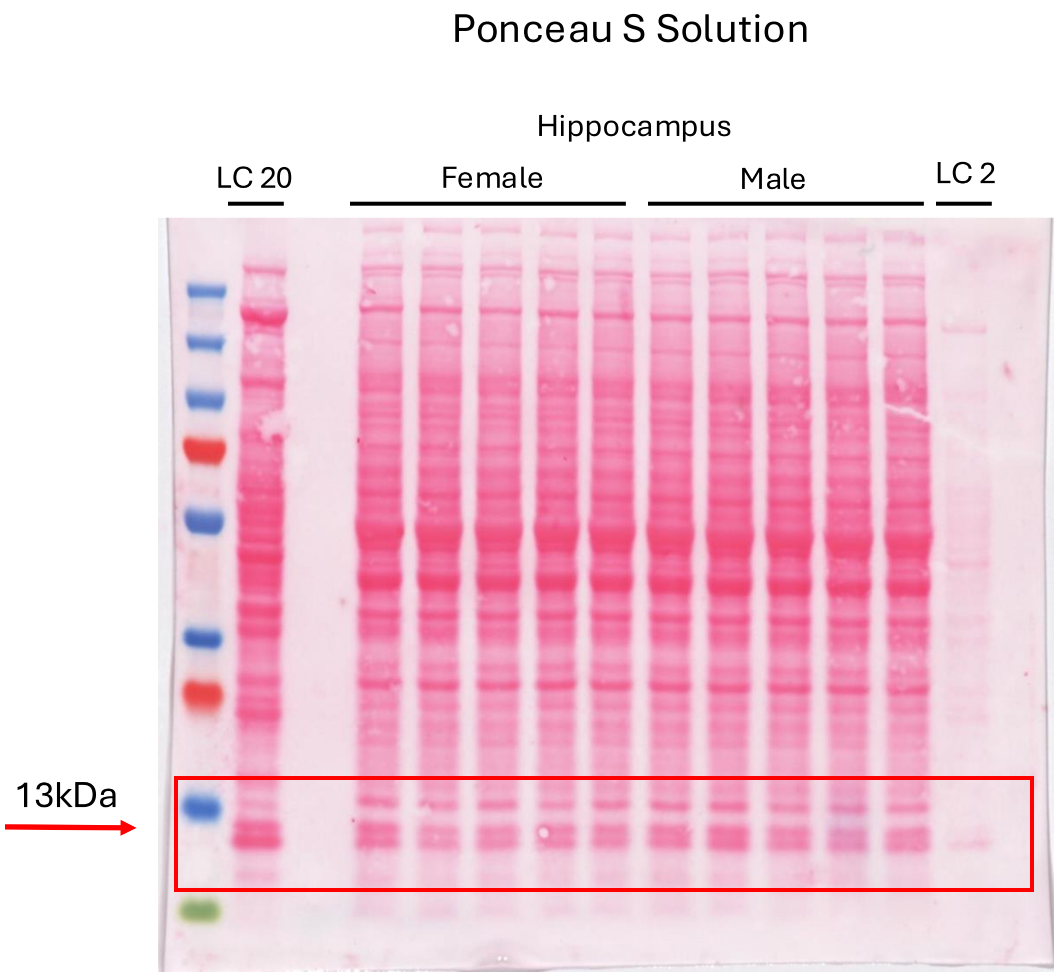

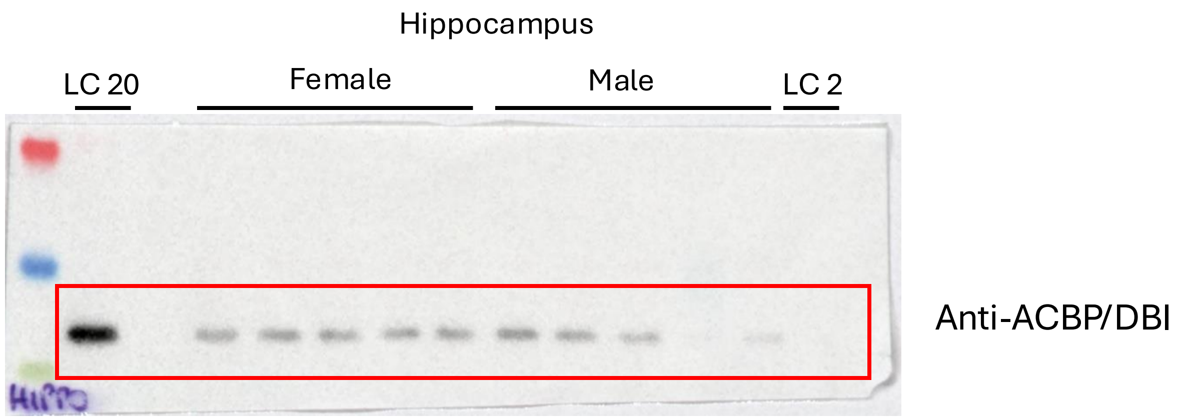

Figure S1

15. Mice Cortex

Ponceau S Solution

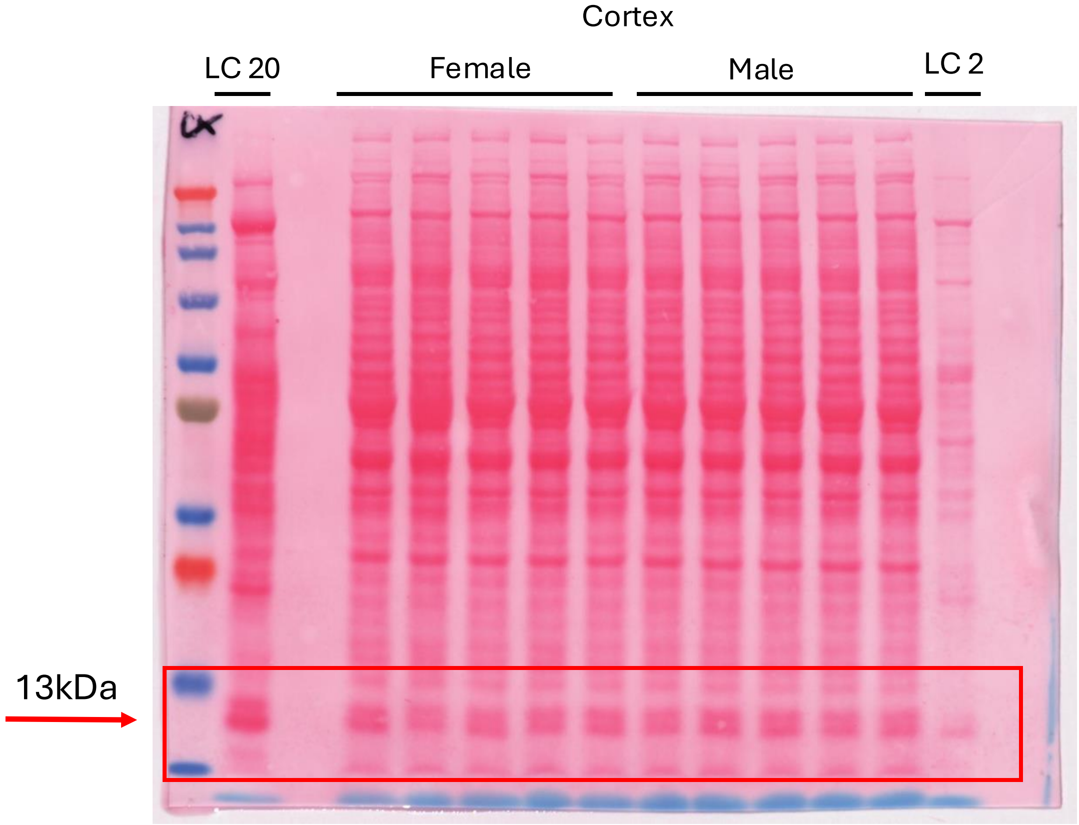

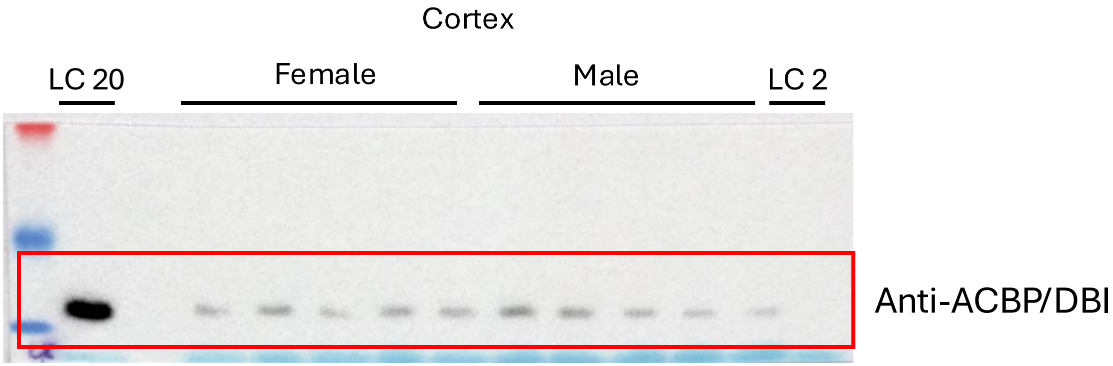

Figure S1
16. Mice Thalamus

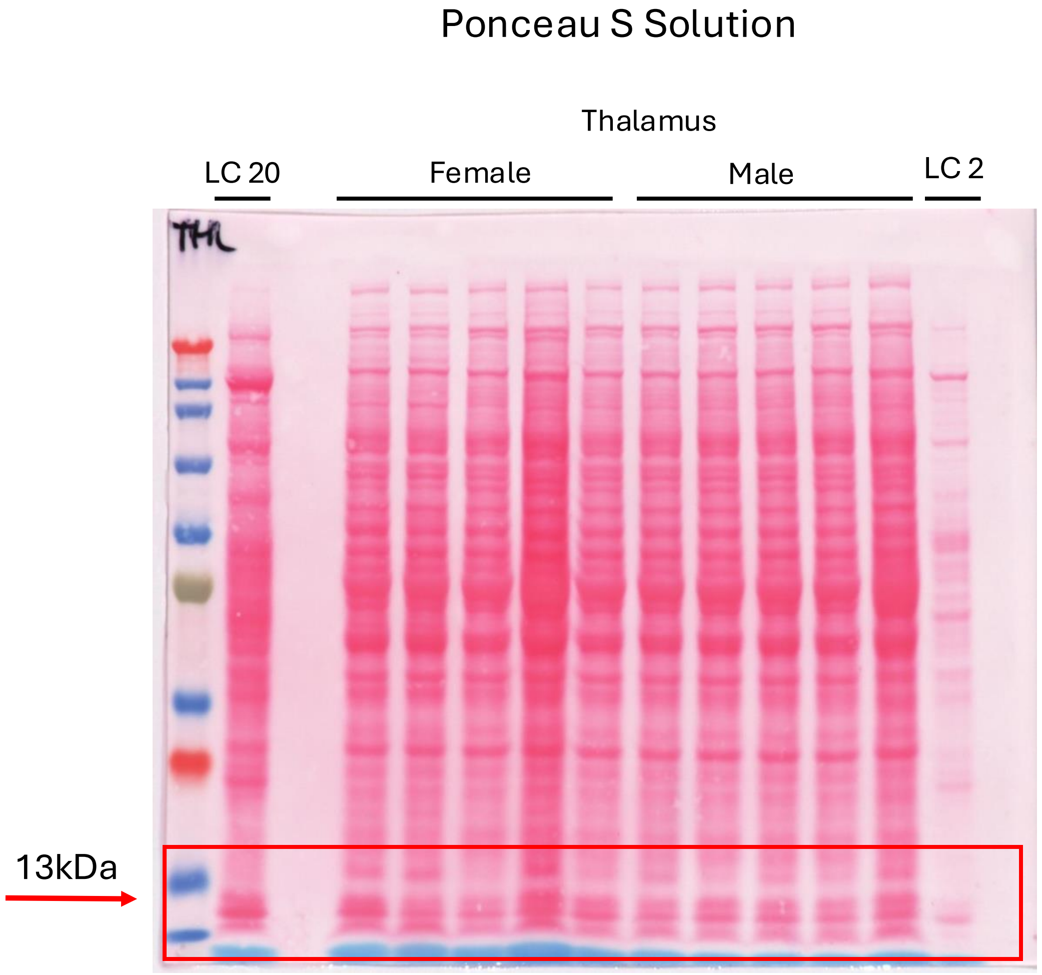

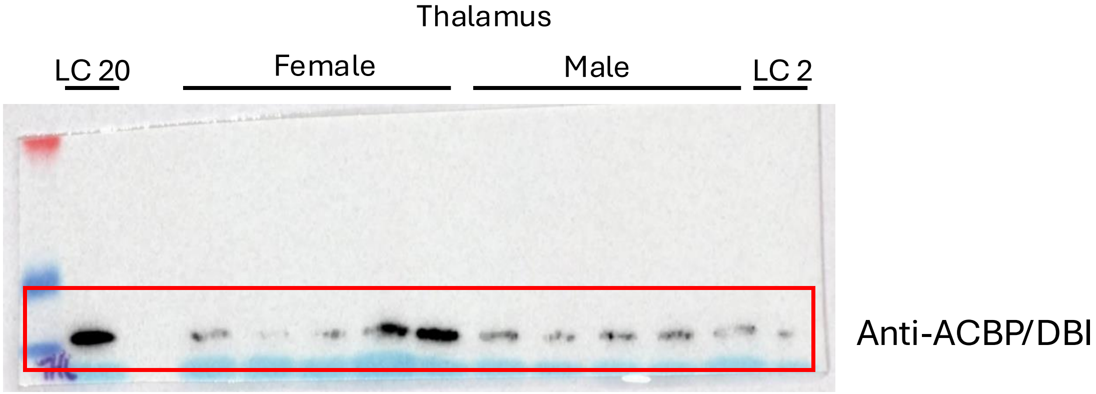

Figure S1

17. Mice Stomach

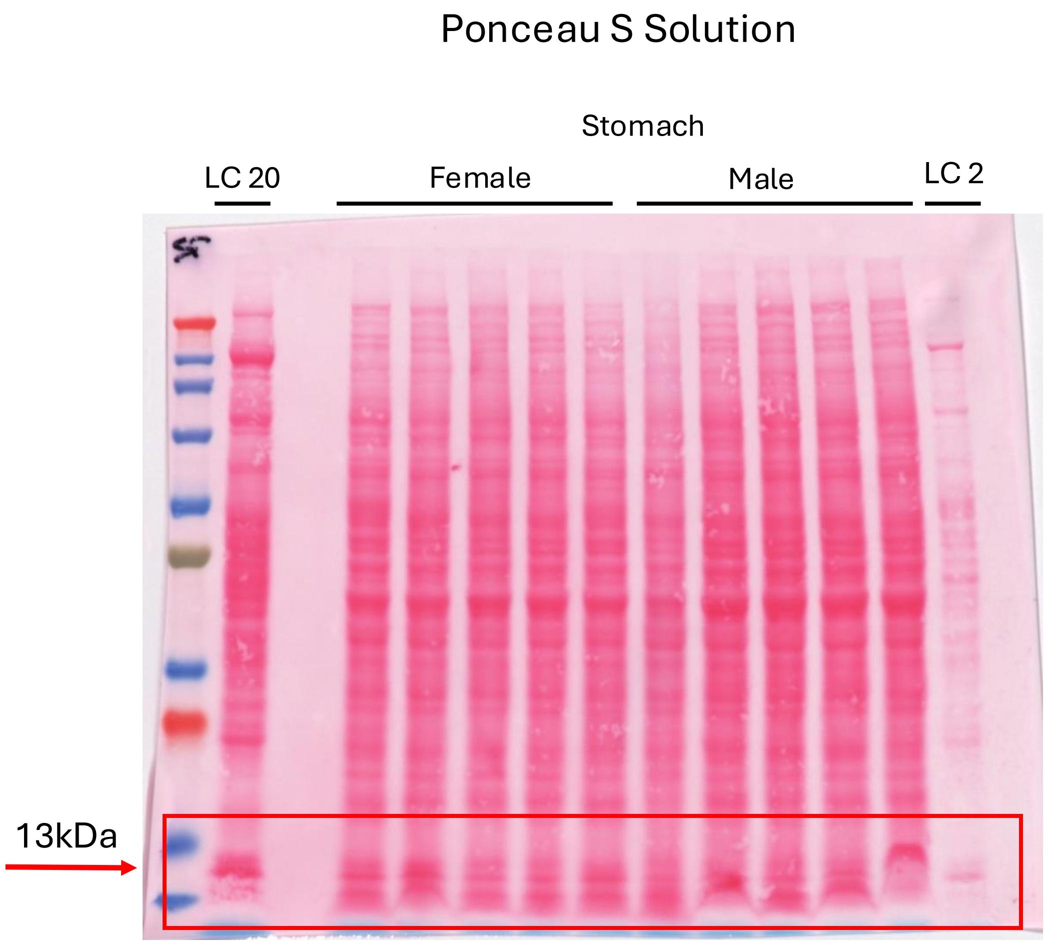

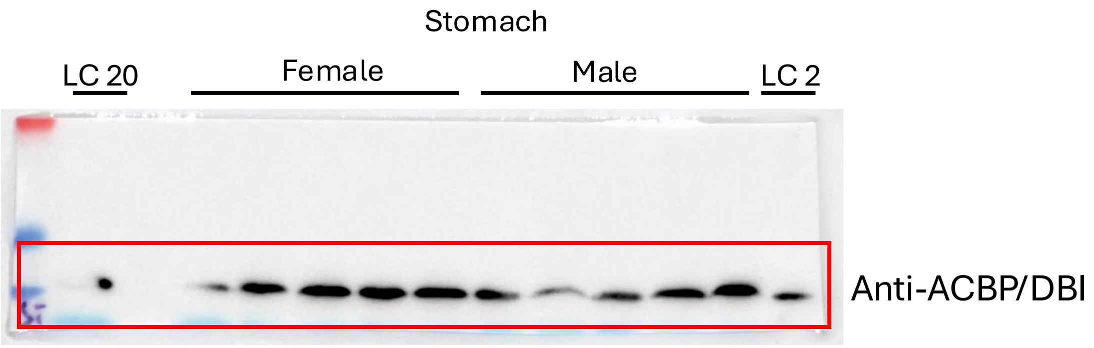

Figure S1
18. Mice Duodenum

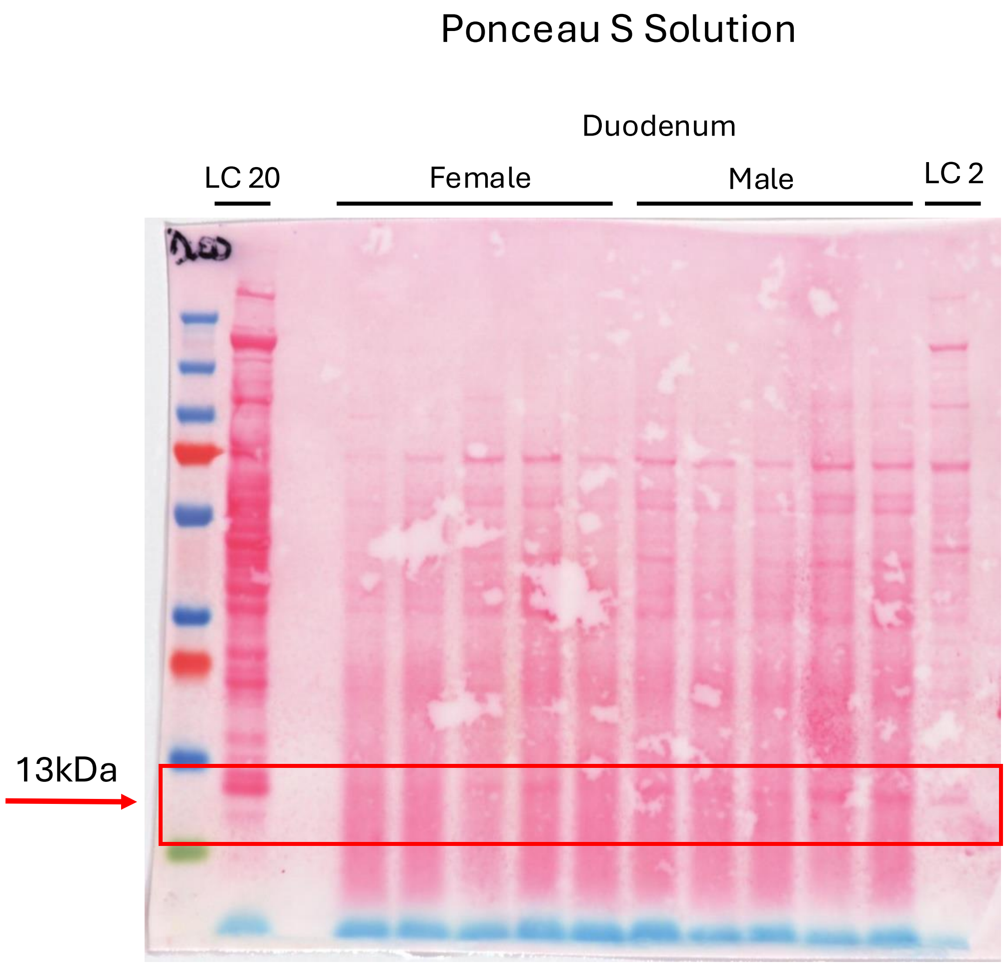

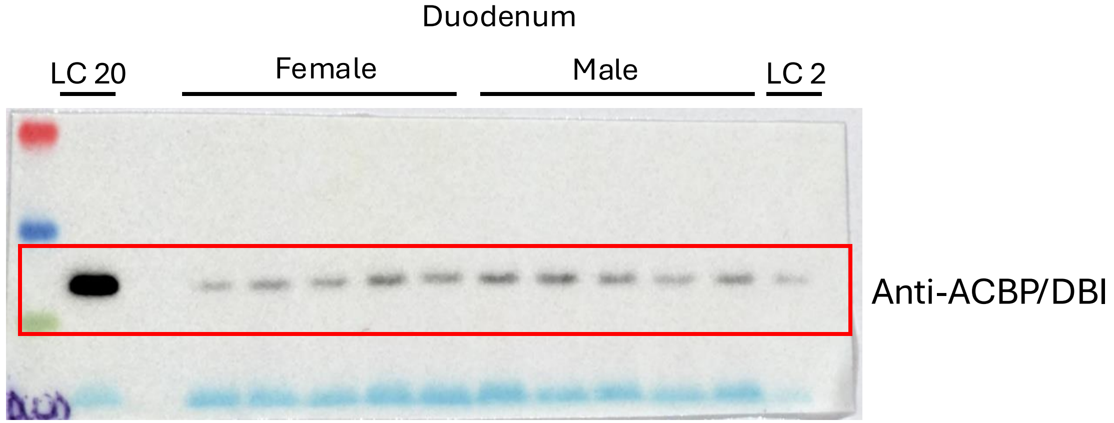

Figure S1
19. Mice Jejunum

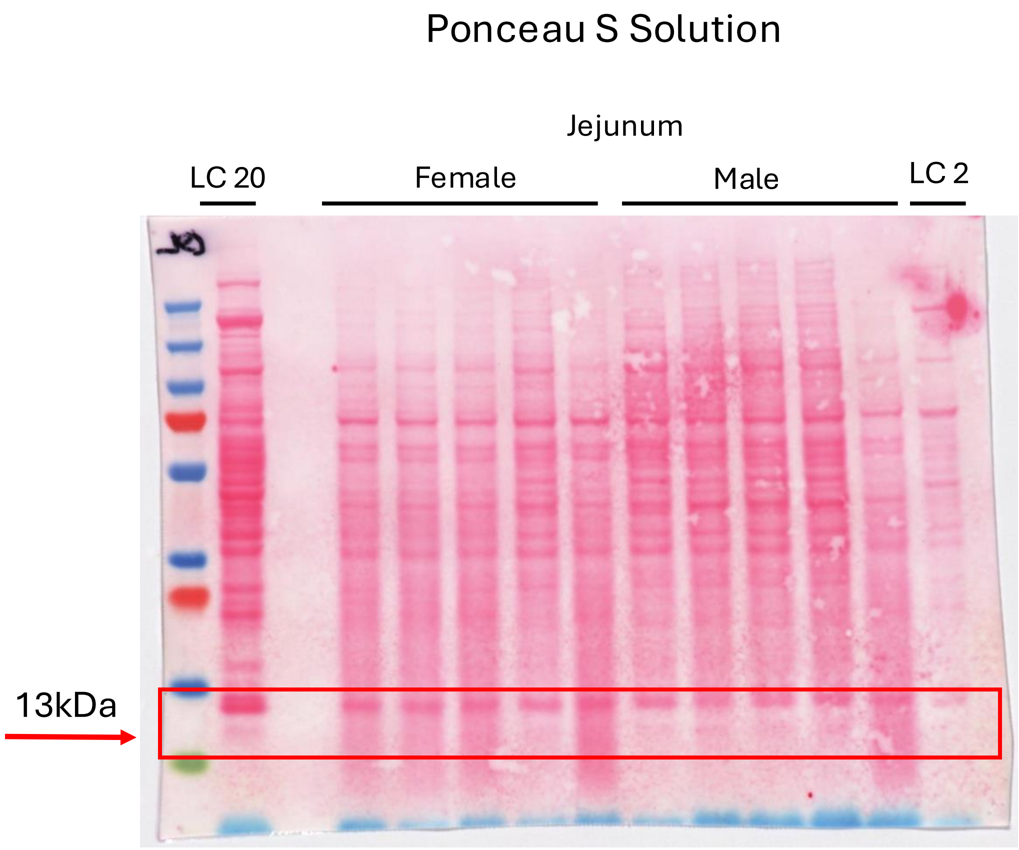

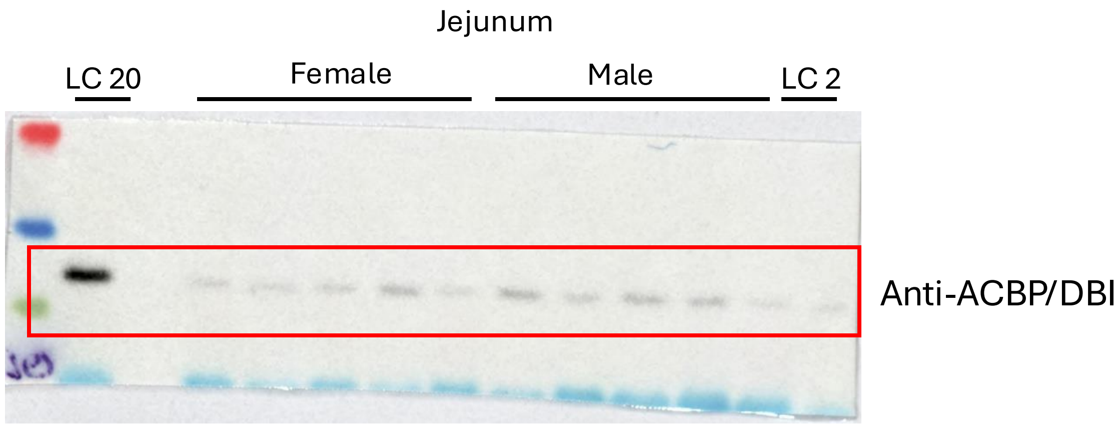

Figure S1

20. Mice Ileum

Ponceau S Solution

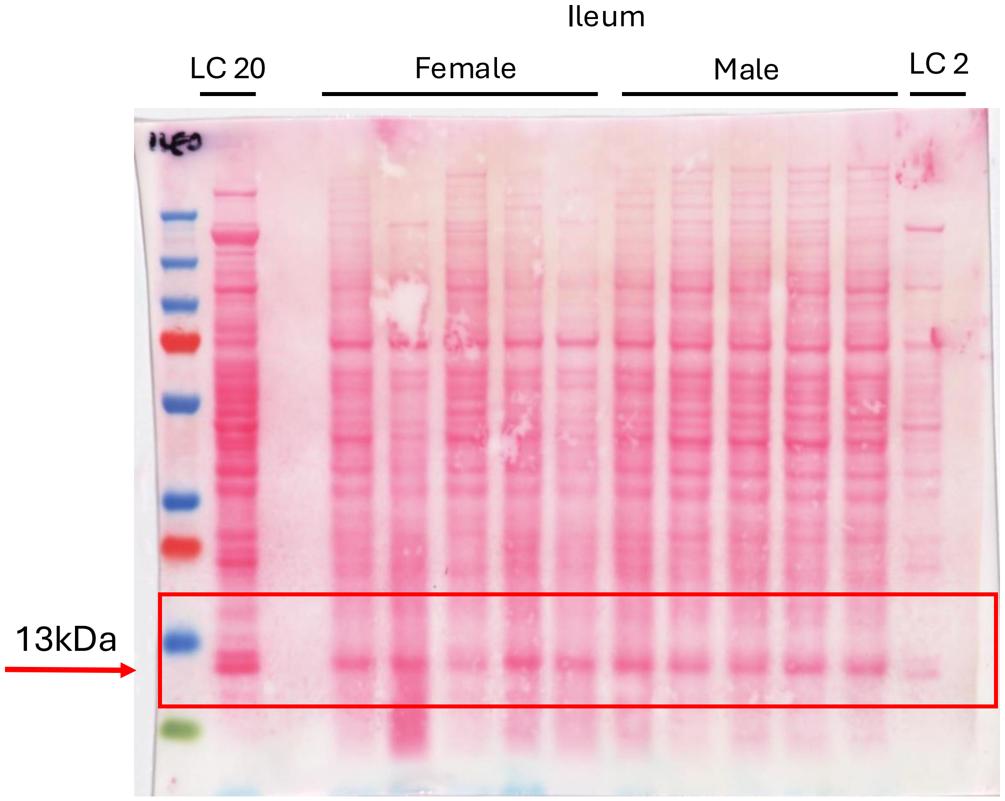

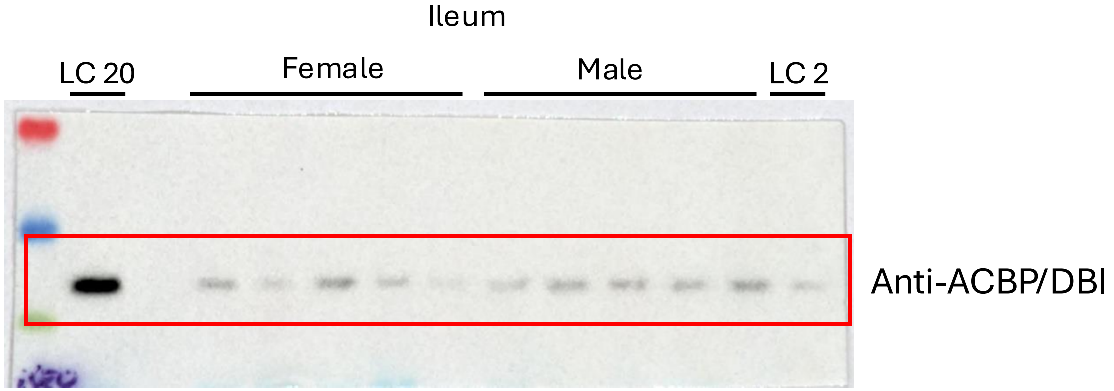

Figure S1

21. Mice Colon

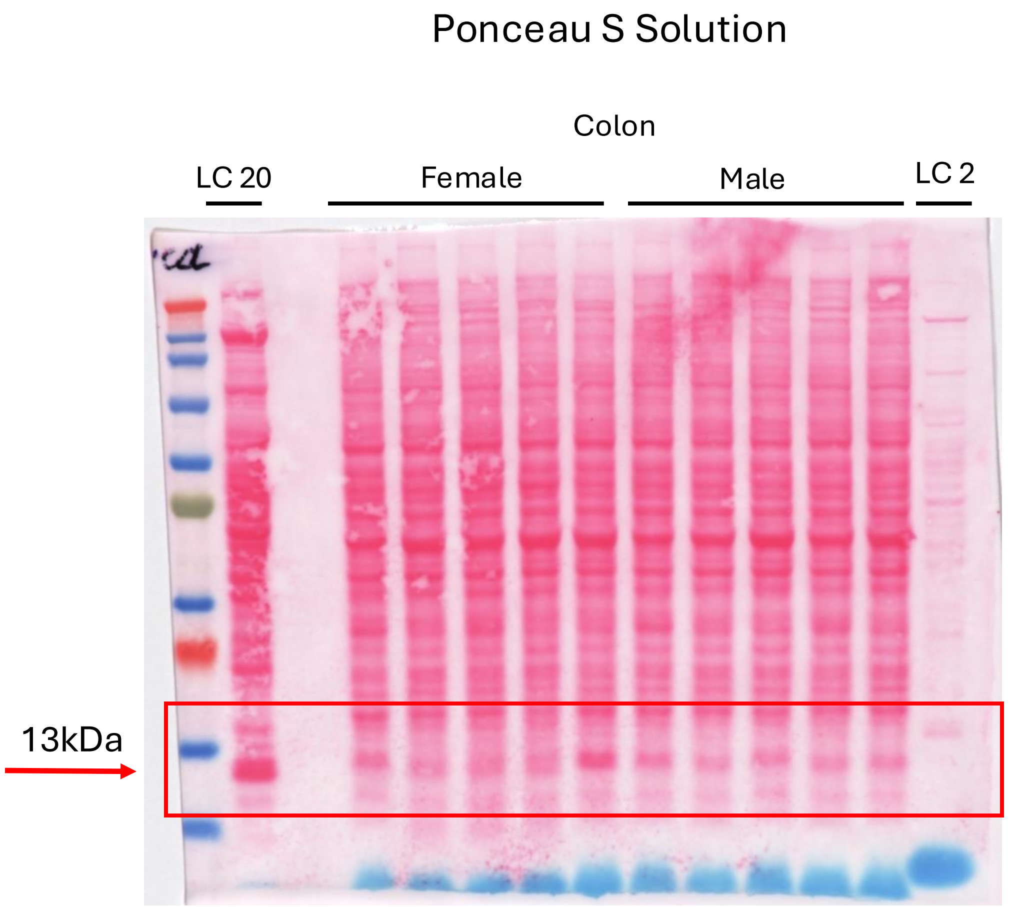

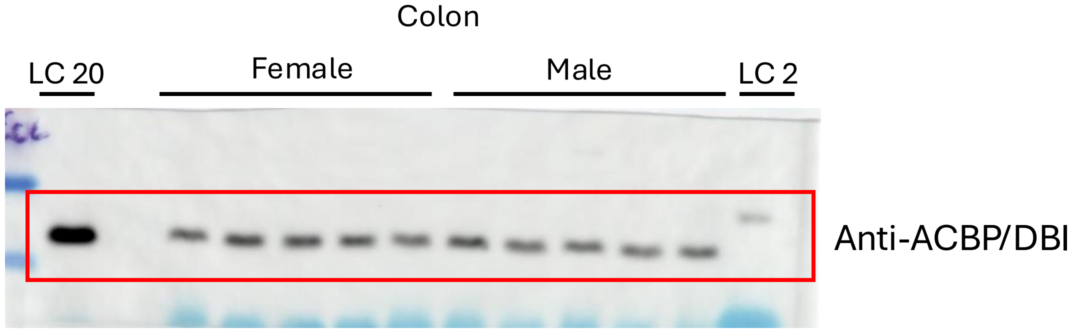

Figure S1

22. Mice Ovary and Testicles

Ponceau S Solution

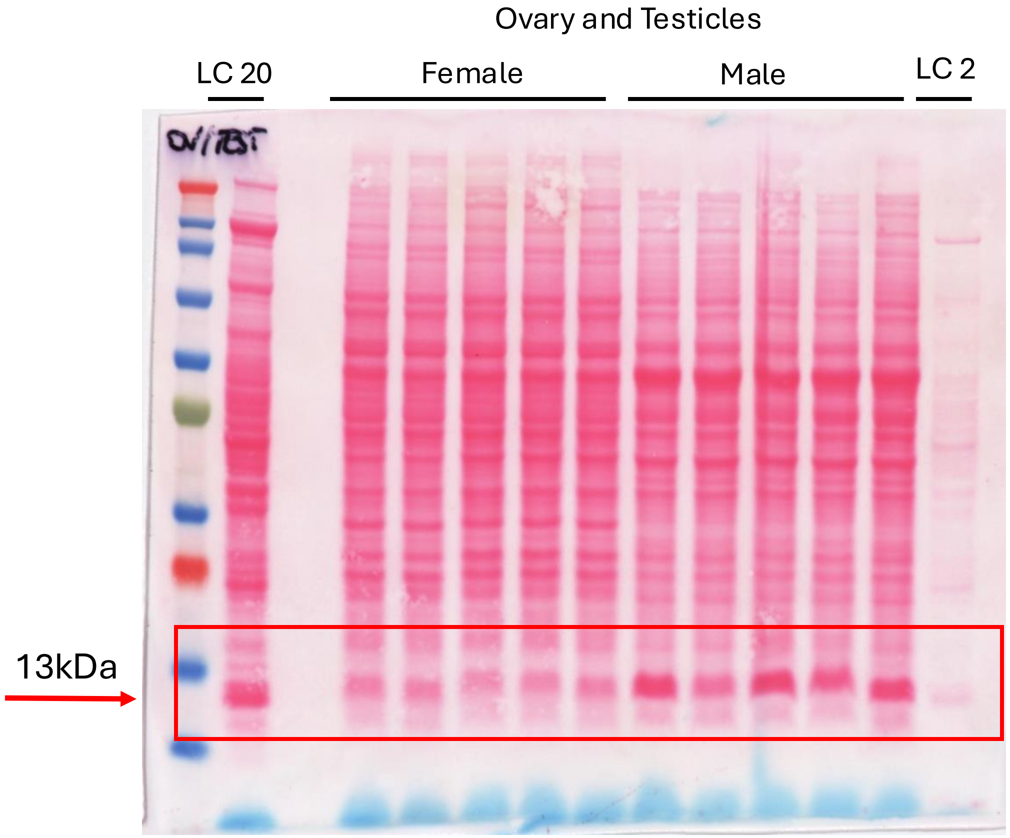

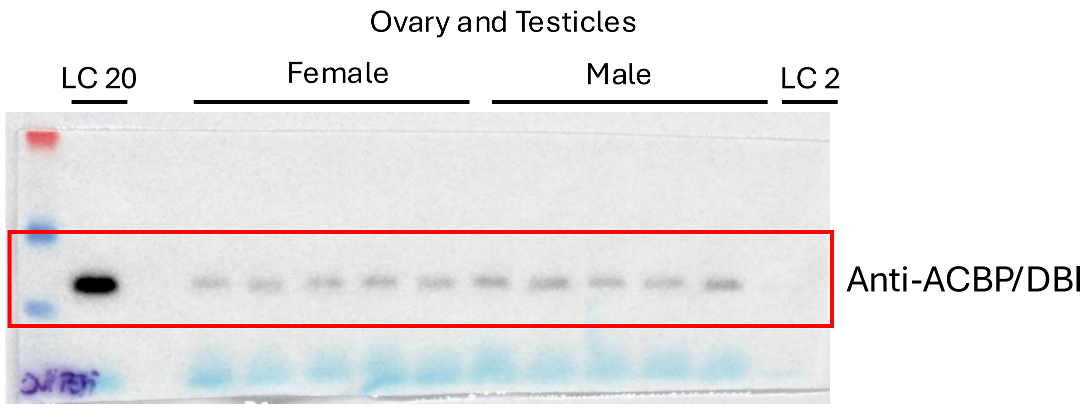

Figure S1

23. Mice Uterus and Seminal Vesicles

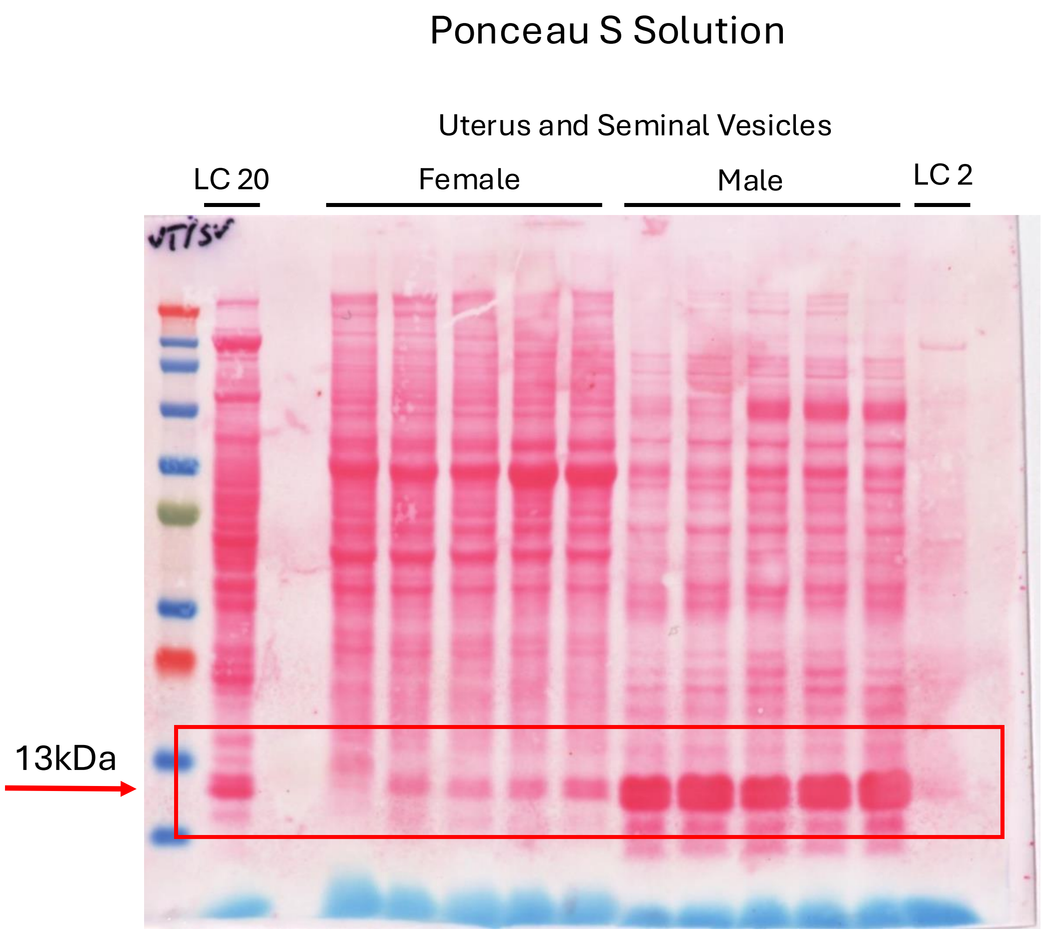

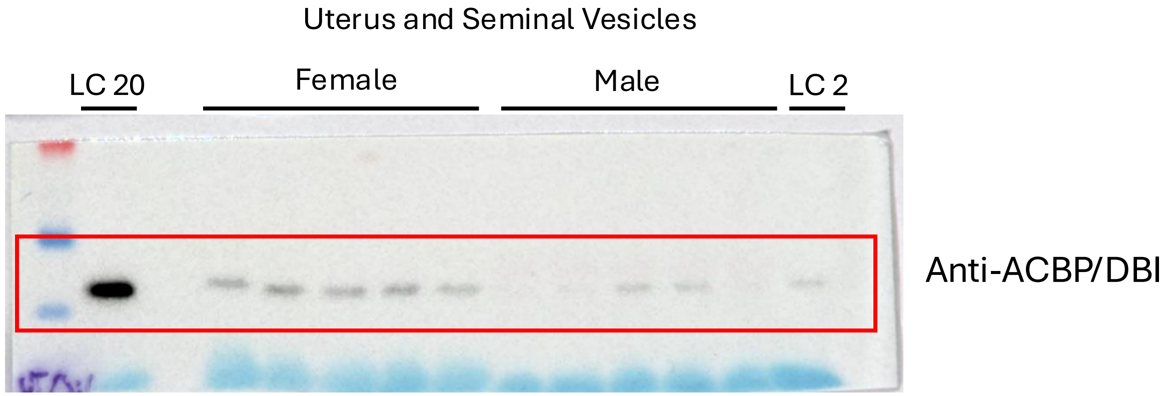

Supplement: Supplementary file 4 — Original Data [file 41419_2025_7447_MOESM4_ESM.pdf]
